# Enantioselective α-Chlorination Reactions of in Situ Generated C1 Ammonium Enolates under Base-Free Conditions

**DOI:** 10.1021/acs.orglett.1c02256

**Published:** 2021-07-28

**Authors:** Lotte Stockhammer, David Weinzierl, Thomas Bögl, Mario Waser

**Affiliations:** † Institute of Organic Chemistry, Johannes Kepler University Linz, Altenbergerstr. 69, 4040 Linz, Austria; ‡ Institute of Analytical Chemistry, Johannes Kepler University Linz, Altenbergerstr. 69, 4040 Linz, Austria

## Abstract

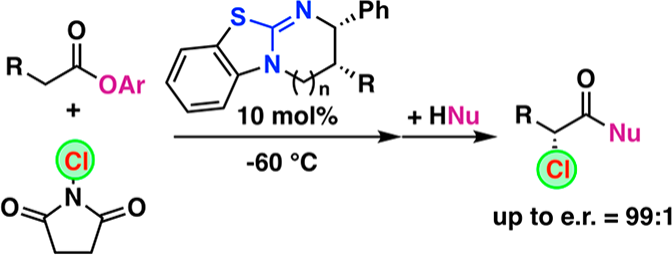

The asymmetric α-chlorination of activated aryl acetic acid esters can be carried out with high levels of enantioselectivities utilizing commercially available isothiourea catalysts under base-free conditions. The reaction, which proceeds via the in situ formation of chiral C1 ammonium enolates, is best carried out under cryogenic conditions combined with a direct trapping of the activated α-chlorinated ester derivative to prevent epimerization, thus allowing for enantioselectivities of up to e.r. 99:1.

Asymmetric α-heterofunctionalization reactions, especially α-halogenations, of prochiral enolate precursors and analogues represent important transformations to access valuable chiral building blocks suited for further manipulations as well as potentially biologically active target molecules.^1^ Over the last several decades, numerous asymmetric (organo)-catalysis-based α-halogenation approaches for, for example, amino acid derivatives,^2^ (cyclic) β-ketoesters,^3^ and oxindoles^4^ (to name a few), have been reported, and the introduction of new concepts and methods is still a topic of considerable interest. Whereas the asymmetric α-halogenation of cyclic pronucleophiles has been very extensively developed,^1−4^ the direct α-halogenation of acyclic enolate precursors, especially simple carboxylic acid (ester)-based precursors, has been a more challenging task. One conceptually very appealing strategy to employ acyclic carboxylic acid equivalents in asymmetric transformations relies on the use of chiral nucleophilic organocatalysts (i.e., chiral amines, pyridine derivatives, and isothioureas).^5,6^ These easily available catalysts can react with ketenes or activated carboxylic acid derivatives (esters, chlorides, or in situ generated anhydrides) to form well-defined chiral C1 ammonium enolates (Scheme 1A). These in situ generated species undergo various α-functionalization reactions with high levels of stereoinduction, followed by the release of the catalyst upon the addition of a nucleophile (Nu).^5−9^ This attack may happen either in an intramolecular fashion when the C1 ammonium enolate is reacted with a suitable dipolar partner (resulting in formal (2 + *n*) cyclizations)^5,6^ or in an intermolecular manner (e.g., the ester group that is cleaved off during the ammonium enolate formation is added again).^7^ This unique concept has over the last several years shown its potential for a variety of asymmetric C–C bond-forming reactions,^5−7^ even in a cooperative manner with transition-metal catalysis.^9^

**Scheme 1:**
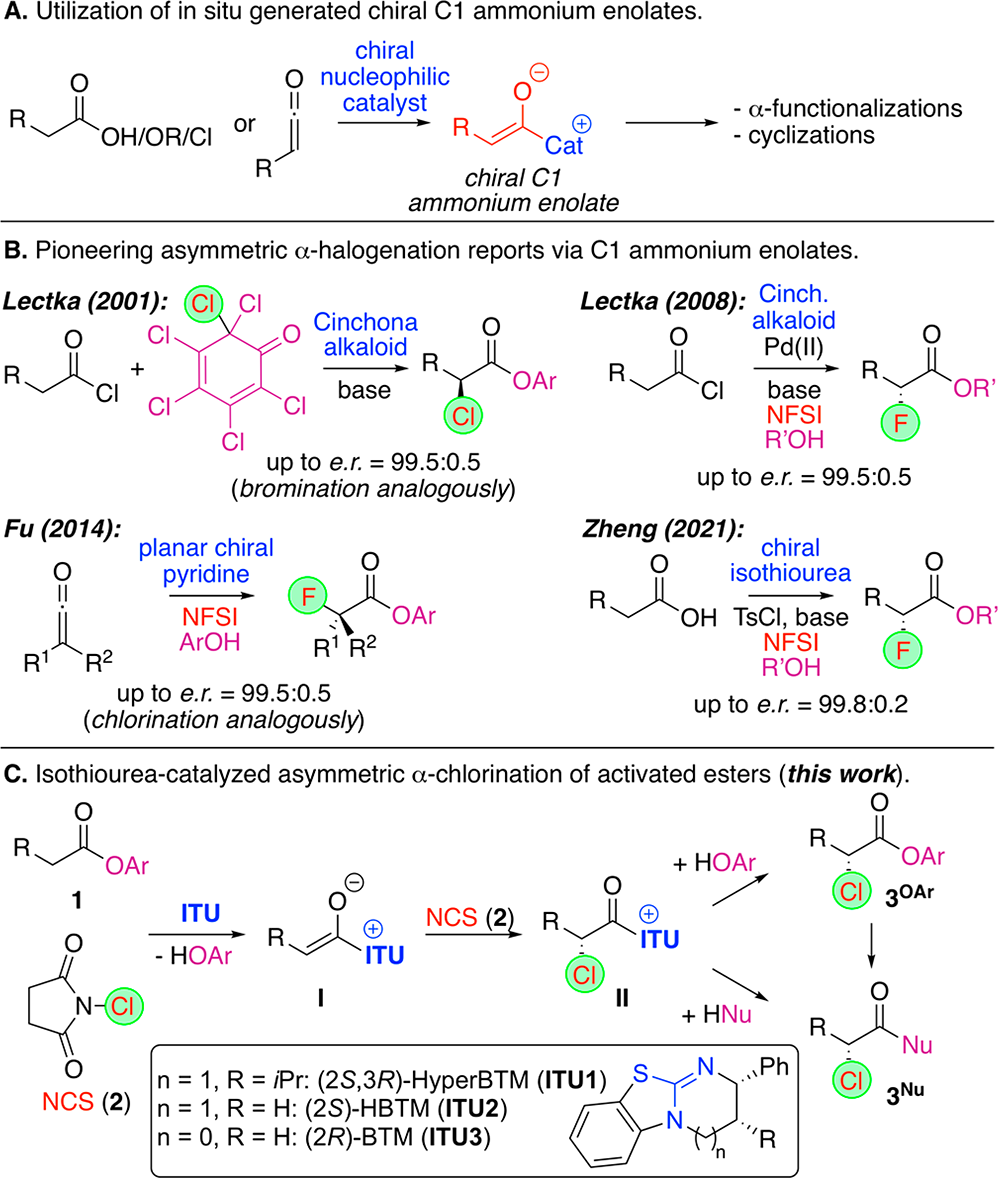
Use of in Situ Generated C1 Ammonium Enolates, Pioneering α-Halogenation Approaches, and the Herein Investigated α-Chlorination

Surprisingly, however, asymmetric α-halogenation approaches have received less attention so far (Scheme 1B), although early impressive reports by Lectka’s group demonstrated the potential of this concept to facilitate asymmetric α-halogenations of acylhalides using Cinchona alkaloid catalysis. (Turnover was achieved by the choice of the proper electrophilic halide-transfer reagent or the addition of an external nucleophile.)^10,11^ In addition, Smith’s and Fu’s groups reported α-halogenations of ketene precursors using either chiral N-heterocyclic carbenes (NHCs)^12^ or planar chiral pyridine catalysts,^13^ underscoring the potential of this concept to access valuable enantioenriched α-halogenated acyclic carboxylic acid derivatives.

Our group recently became interested in asymmetric α-chlorination reactions,^14^ and considering the value of α-chlorinated carbonyl compounds^15^ and the unique potential of C1 ammonium enolate chemistry to facilitate asymmetric α-functionalizations of simple carboxylic acid derivatives, we thought about developing the, to the best of our knowledge, unprecedented α-chlorination of simple activated esters **1** (Scheme 1C). We reasoned that the use of well-established isothioureas (i.e., the commercially available **ITU1–3**)^6,16,17^ in combination with *N*-chlorosuccinimide (NCS, **2**) would hereby provide an entry to the α-chlorinated derivatives **3**. This reaction may be first steered toward the aryloxide-rebound product **3**^**Oar**^, which can then be converted into other products by the addition of a nucleophile in a separate step, or it may also be possible to carry out the reaction in the presence of an external nucleophile, directly giving products **3**^**Nu**^. Furthermore, it should be possible to carry out the reaction either totally in the absence of an external base,^18^ or at least using only catalytic amounts of base, considering the fact that the released succinimide should be capable of serving as the base required for enolate formation. Encouragingly, during the finalization of this manuscript, Zheng and coworkers reported an elegant complementary approach for the α-fluorination of free carboxylic acids (which are activated in situ upon the addition of TsCl) in the presence of a newly designed [2.2]paracyclophane-based isothiourea catalyst (Scheme 1B),^19^ thus underscoring the potential of this catalysis concept.

We started by carrying out the α-chlorination of the pentafluorophenyl ester **1a**. (Table 1 gives an overview of the most significant screening results.) The first room-temperature experiments in tetrahydrofuran (THF) showed good conversion to the target **3a**^**OAr**^ in the absence of any external base, substantiating our initial proposal. Unfortunately, this product turned out to be rather unstable during the work up and purification. Thus we changed our strategy in such a way that we first carried out the ITU-catalyzed α-chlorination (at the given temperature for the indicated time) followed by the addition of MeOH to access the stable ester **3a**^**OMe**^ instead, which could easily be accessed and analyzed by high-performance liquid chromatography (HPLC) using a chiral stationary phase.^20^ Unfortunately, product **3a**^**OMe**^ was isolated only in a racemic manner, independent of the used ITU catalyst (entries 1–3). In addition, we also observed the formation of small quantities of the dichlorinated product **4a** (usually <5% when using 2 equiv of NCS), and the amount of **4a** significantly increased when a larger excess of NCS was used.^21^

**Table 1:**
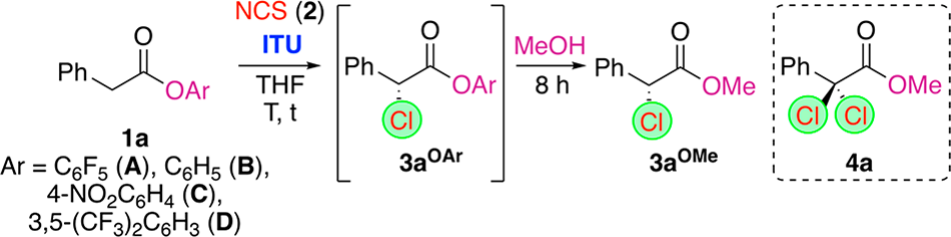
Optimization of Reaction Conditions^a^

entry	Ar	**ITU** (mol %)	*T* (°C)	*t* (h)	conv. (%)^b^	**3a**^**OMe**^ (%)^c^	e.r.^d^
1	**A**	**ITU1** (20%)	25	20	>95	73	50:50
2	**A**	**ITU2** (20%)	25	20	95	69	50:50
3	**A**	**ITU3** (20%)	25	20	90	67	50:50
4	**A**	**ITU1** (20%)	–40^e^	20	>95	69	50:50
5	**A**	**ITU1** (20%)	–40^f^	20	>95	67	75:25
6	**A**	**ITU1** (20%)	–60^f^	20	80	68	95:5
7	**A**	**ITU1** (20%)	–80^f^	20	45	n.d.	97:3
8	**A**	**ITU1** (10%)	–80^f^	20	40	n.d.	98:2
9	**A**	**ITU1** (20%)	–60^f^	40	>95	82	95:5
10	**B**	**ITU1** (20%)	–60^f^	40	0		
11	**C**	**ITU1** (20%)	–60^f^	40	85	66	93:7
12	**D**	**ITU1** (20%)	–60^f^	40	80	61	90:10
13	**A**	**ITU1** (10%)	–60^f^	40	>95	79	96:4
14	**A**	**ITU1** (5%)	–60^f^	40	45	n.d.	97:3
15	**A**	**ITU1** (40%)	–60^f^	40	>95	81	85:15
16	**A**	**Se-ITU1** (10%)^g^	–60^f^	40	80	68	99:1
17	**A**	**ITU2** (10%)	–60^f^	40	70	51	95:5
18	**A**	**ITU3** (10%)	–60^f^	40	75	67	99:1
19	**A**	**ITU3** (10%)	–60^f^	63	>95	91	99:1
20	**A**	**ITU3** (10%)	–60^h^	63	90	84	99:1

a All reactions were carried out using 0.1 mmol **1** and 0.2 mmol **2** in THF (0.1 M with respect to **1**), unless otherwise stated.

b Conversion of **1** judged by ^1^H NMR of the crude product.

c Isolated yields.

d Determined by HPLC using a chiral stationary phase. Absolute configuration of the major (R) enantiomer was assigned by the comparison of the retention time order and its (−) rotation with previous reports.^20^

e MeOH added after warming the reaction mixture to r.t.

f MeOH added at the cryogenic reaction temperature followed by a slow warm up to r.t. over 8 h.

g Se-HyperBTM analogue was recently introduced by Smith’s group.^23^

h MeOH (2 equiv) present during the whole reaction.

Because **ITU1** was found to be slightly more active compared with **ITU2** and **ITU3** (entries 1–3), further testing at lower temperatures was next done with **ITU1**. Interestingly, when the chlorination was carried out at −40 °C followed by a MeOH quench at room temperature (r.t.) (entry 4), product **3a**^**OMe**^ was still formed only as a racemate, whereas MeOH addition at low temperature (entry 5) resulted in notable levels of enantioenrichment. Studies concerning the configurational stability of product **3a**^**OMe**^ showed that this compound slowly epimerized in the presence of external bases (e.g., Et_3_N), whereas no loss of optical purity was observed after silica gel column chromatography and upon prolonged dissolution in nonbasic solvents. Thus the results reported in entries 4 and 5 can be rationalized by a rapid epimerization of **3a**^**OAr**^ in the presence of the catalyst, most likely via the formation of the α-chlorinated catalyst-bound intermediate **II**(22) (Scheme 1C), which shows increased acidity in the α-position (also rationalizing the dichlorination toward **4**). A quench at low temperature, on the contrary, allows this epimerization to be overcome by forming the more stable **3a**^**OMe**^, which no longer allows the formation of **II**. With these insights at hand, we further lowered the temperature, resulting in good enantioselectivities at −60 °C or lower (entries 6 and 7). Unfortunately, the reaction significantly slowed down at −80 °C (entries 7 and 8), and we thus next carried out further optimizations at −60 °C for 40 h (entries 9–19; all reactions were run in THF because toluene and CH_2_Cl_2_ did not allow for any product formation at all). Testing alternative esters **1a** (entries 10–12) proved the crucial nature of the aryloxide. Whereas, as expected, phenoxide (**B**) did not allow for any conversion, the electron-poor aryl groups **C** and **D** allowed for good conversion but with a slightly lower e.r. compared with the initially used **A**. Changing the catalyst loading lead to a surprising observation, as lower amounts of catalysts allowed for slightly improved enantioselectivities (entries 13 and 14), whereas a higher loading had a detrimental effect (entry 15).

Finally, we tested **ITU2** and **ITU3** as well as the recently introduced HyperBTM isoselenourea (Se instead of S) **Se-ITU1**(23) under the optimized conditions (entries 16–18). Remarkably, whereas the selectivity of HBTM (**ITU2**) was similar to that of **ITU1** (compare entries 13 and 17), isoselenourea **Se-ITU1** and BTM (**ITU3**) both allowed for significantly improved enantioselectivities of 99:1, albeit with slightly lower conversions after 40 h reaction times (entries 16 and 18). In addition, in both cases, the formation of the dichloro compound **4a** was almost completely suppressed. Gratifyingly, the slightly lower conversion with these catalysts compared with **ITU1** could easily be overcome by longer reaction times (entry 19). Furthermore, we also tested whether it may be possible to add MeOH right from the beginning, and the outcome was very similar (entry 20); however, because small amounts of methyl phenylacetate (formed via the transesterification of **1a**) were formed, too, we used the stepwise protocol (entry 19) with the commercially available **ITU3** to investigate the application scope (Scheme 2).

**Scheme 2:**
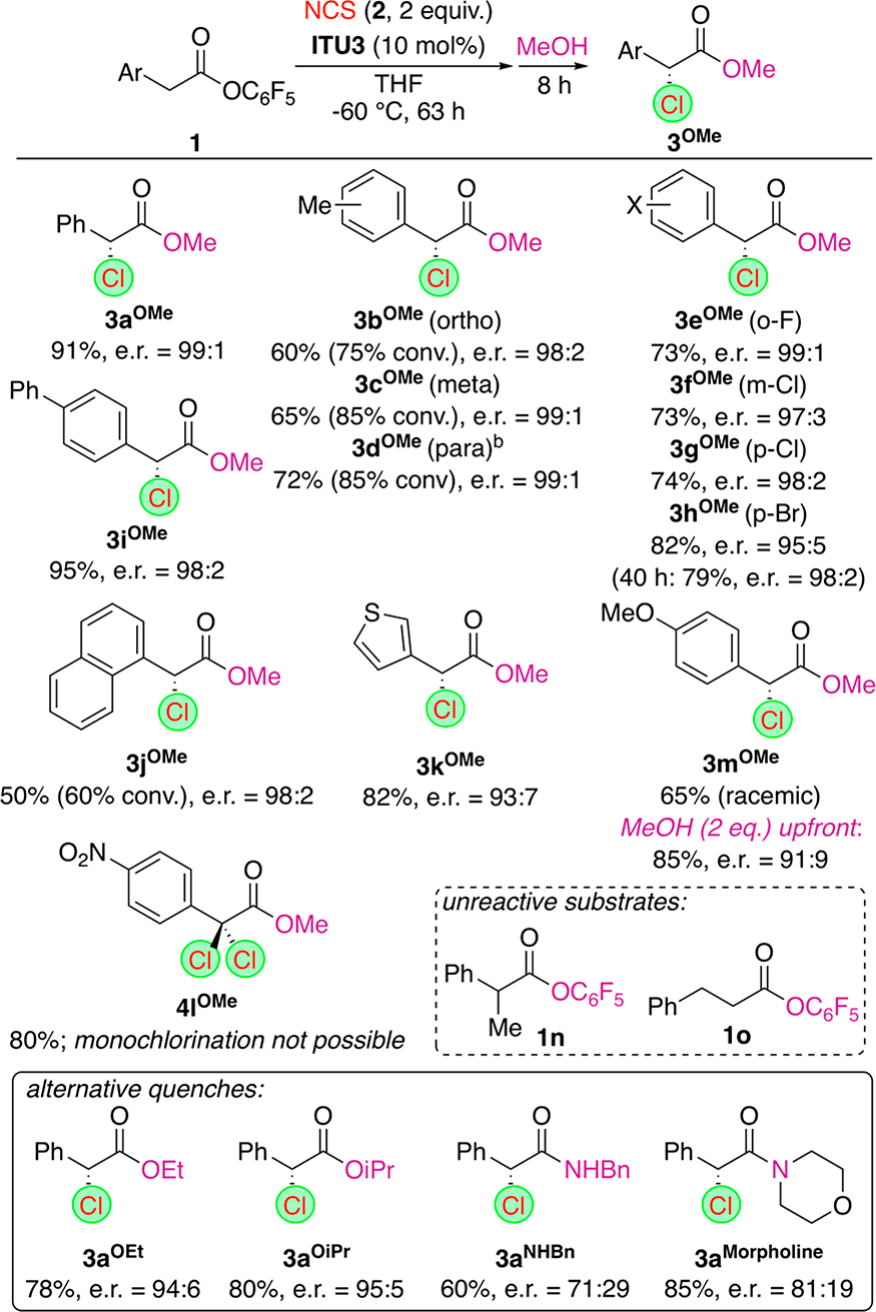
Application Scope All reactions were carried out using 0.1 mmol **1** and showed >95% conversion unless otherwise stated. Repeated on a 1 mmol scale, providing **3d**^**OMe**^ in 71% yield and with e.r. 99:1.

Interestingly, the presence of electron-neutral organic substituents and halides in different positions as well as alternative aryl moieties primarily affected the conversion rate but allowed for reasonable enantioselectivities in all cases (**3a**^**OMe**^–**3k**^**OMe**^). Interesting observations were made when the syntheses of the Br-substituted **3h**^**OMe**^, the methoxy-containing **3m**^**OMe**^ and the nitro-derivative **3l**^**OMe**^ were attempted. Target **3h**^**OMe**^ was obtained with e.r. 98:2 after 40 h (complete conversion) but with a slightly reduced selectivity of 95:5 after 63 h only,^24^ whereas the presence of the more electron-donating methoxy group para to the reaction center resulted in the formation of only racemic **3m**^**OMe**^. These results may primarily be attributed to the epimerization of the catalyst-bound α-Cl intermediates **II** under the reaction conditions. Whereas this process is not that strongly pronounced for electron-neutral aryl substituents, the presence of more electron-donating substituents (i.e., a methoxy group) para to the benzylic chlorination site significantly increases the epimerization rate. (The *p*-MeO group most likely triggers the in situ cleavage of the Cl group via the formation of a quinone-methide-type-stabilized benzylic carbocation followed by an unselective readdition of the Cl.) It is noteworthy, this epimerization could be mainly suppressed by the addition of MeOH (2 equiv) right from the beginning (compare with entry 20, Table 1), which allowed for a reasonably selective direct synthesis of **3m**^**OMe**^. In sharp contrast, the presence of the NO_2_ group resulted in the quantitative formation of only the dichloro product **4l**^**OMe**^ (the same was observed when MeOH was added up front), which can be hereby rationalized by the increased reactivity of the corresponding intermediate **II**. (Using just 1 equiv of NCS gave **4l**^**OMe**^ in addition to starting material **1l** only!) Unfortunately, the α-alkylated ester **1n** and the homologous phenylpropionic acid ester **1o** were not found to be reactive, which is in accordance with previous observations for other C1 ammonium enolate applications.^5−7,9,19^

Finally, we also carried out quenches with a few other aliphatic alcohols and amines. The use of ethanol and i-PrOH (products **3a**^**OEt**^ and **3a**^**OiPr**^) resulted in slightly lower enantioselectivities compared with the use of MeOH, which can be rationalized by a slightly slower esterification with these alcohols, thus allowing for some epimerization of intermediates **II**. Unfortunately, the use of amines turned out to be much more difficult, resulting in a significant loss of enantiopurity (products **3a**^**NHBn**^ and **3a**^**Morpholine**^). Considering the already observed configurational lability of intermediates **II**, especially under basic conditions, these results came as no big surprise, and they underscore the need for the carefully optimized reaction conditions developed herein.

In conclusion, it was possible to introduce a highly enantioselective α-chlorination protocol of activated esters **1** under chiral isothiourea catalysis, but the reaction as such requires carefully fine-tuned conditions to suppress the epimerization of the reactive catalyst-bound intermediates.

## Supporting Information Available

Experimental and analytic details (including copies of HPLC traces) (PDF)

## References

[ref1] aOestreich M. Strategies for Catalytic Asymmetric Electrophilic α Halogenation of Carbonyl Compounds. Angew. Chem., Int. Ed. 2005, 44, 2324–2327. 10.1002/anie.200500478. 15827954

[ref2] Eder I.; Haider V.; Zebrowski P.; Waser M. Recent Progress in the Asymmetric Syntheses of α-Heterofunctionalized (Masked) α- and β-Amino Acid Derivatives. Eur. J. Org. Chem. 2021, 2021, 202–219. 10.1002/ejoc.202001077.

[ref3] Govender T.; Arvidsson P. I.; Maguire G. E. M.; Kruger H. G.; Naicker T. Enantioselective Organocatalyzed Transformations of β-Ketoesters. Chem. Rev. 2016, 116, 9375–9437. 10.1021/acs.chemrev.6b00156. 27463615

[ref4] Freckleton M.; Baeza A.; Benavent L.; Chinchilla R. Asymmetric Organocatalytic Electrophilic Heterofunctionalization of Oxindoles. Asian J. Org. Chem. 2018, 7, 1006–1014. 10.1002/ajoc.201800146.

[ref5] aFu G. C. Asymmetric Catalysis with “Planar-Chiral” Derivatives of 4-(Dimethylamino)pyridine. Acc. Chem. Res. 2004, 37, 542–547. 10.1021/ar030051b. 15311953

[ref6] aTaylor J. E.; Bull S. D.; Williams J. M. J. Amidines, isothioureas, and guanidines as nucleophilic catalysts. Chem. Soc. Rev. 2012, 41, 2109–2121. 10.1039/c2cs15288f. 22234578

[ref7] Hartley W. C.; O’Riordan T. J. C.; Smith A. D. Aryloxide-Promoted Catalyst Turnover in Lewis Base Organocatalysis. Synthesis 2017, 49, 3303–3310. 10.1055/s-0036-1589047.

[ref8] West T. H.; Daniels D. S. B.; Slawin A. M. Z.; Smith A. D. An Isothiourea-Catalyzed Asymmetric [2,3]-Rearrangement of Allylic Ammonium Ylides. J. Am. Chem. Soc. 2014, 136, 4476–4479. 10.1021/ja500758n. 24588738

[ref9] aSchwarz K. J.; Amos J. L.; Klein J. C.; Do D. T.; Snaddon T. N. Uniting C1-Ammonium Enolates and Transition Metal Electrophiles via Cooperative Catalysis: The Direct Asymmetric α-Allylation of Aryl Acetic Acid Esters. J. Am. Chem. Soc. 2016, 138, 5214–5217. 10.1021/jacs.6b01694. 27028057

[ref10] aWack H.; Taggi A. E.; Hafez A. M.; Drury W. J.; Lectka T. Catalytic, Asymmetric α-Halogenation. J. Am. Chem. Soc. 2001, 123, 1531–1532. 10.1021/ja005791j. 11456741

[ref11] aPaull D. H.; Scerba M. T.; Alden-Danforth E.; Widger L. R.; Lectka T. Catalytic, Asymmetric a-Fluorination of Acid Chlorides: Dual Metal-Ketene Enolate Activation. J. Am. Chem. Soc. 2008, 130, 17260–17261. 10.1021/ja807792c. 19049284 PMC2651145

[ref12] Douglas J.; Ling K. B.; Concellon C.; Churchill G.; Slawin A. M. Z.; Smith A. D. NHC-Mediated Chlorination of Unsymmetrical Ketenes: Catalysis and Asymmetry. Eur. J. Org. Chem. 2010, 2010, 5863–5869. 10.1002/ejoc.201000864.

[ref13] aLee E. C.; McCauley K. M.; Fu G. C. Catalytic Asymmetric Synthesis of Tertiary Alkyl Chlorides. Angew. Chem., Int. Ed. 2007, 46, 977–979. 10.1002/anie.200604312. 17211906

[ref14] Stockhammer L.; Schörgenhumer J.; Mairhofer C.; Waser M. Asymmetric α-chlorination of β-ketoesters using hypervalent iodine-based Cl-transfer reagents in combination with Cinchona alkaloid catalysts. Eur. J. Org. Chem. 2021, 2021, 82–86. 10.1002/ejoc.202001217. PMC7821243 33519300

[ref15] aShibatomi K.; Narayama A. Catalytic Enantioselective α-Chlorination of Carbonyl Compounds. Asian J. Org. Chem. 2013, 2, 812–823. 10.1002/ajoc.201300058.

[ref16] aBirman V. B.; Li X. Benzotetramisole: A Remarkably Enantioselective Acyl Transfer Catalyst. Org. Lett. 2006, 8, 1351–1354. 10.1021/ol060065s. 16562889

[ref17] Maji B.; Joannesse C.; Nigst T. A.; Smith A. D.; Mayr H. Nucleophilicities and Lewis Basicities of Isothiourea Derivatives. J. Org. Chem. 2011, 76, 5104–5112. 10.1021/jo200803x. 21568333

[ref18] aYoung C. M.; Stark D. G.; West T. H.; Taylor J. E.; Smith A. D. Exploiting the Imidazolium Effect in Base-free Ammonium Enolate Generation: Synthetic and Mechanistic Studies. Angew. Chem., Int. Ed. 2016, 55, 14394–14399. 10.1002/anie.201608046. 27762045

[ref19] Yuan S.; Liao C.; Zheng W.-H. [2.2]Paracyclophane-Based Isothiourea-Catalyzed Highly Enantioselective α-Fluorination of Carboxylic Acids. Org. Lett. 2021, 23, 4142–4146. 10.1021/acs.orglett.1c01046. 33988375

[ref20] aHaughton L.; Williams J. M. J. Enzymatic Hydrolysis and Selective Racemisation Reactions of α-Chloro Esters. Synthesis 2001, 2001, 943–946. 10.1055/s-2001-13395.

[ref21] Tao J.; Tran R.; Murphy G. K. Dihaloiodoarenes: α,α-Dihalogenation of Phenylacetate Derivatives. J. Am. Chem. Soc. 2013, 135, 16312–16315. 10.1021/ja408678p. 24152071

[ref22] Catalyst-bound intermediates **I** and **II** could be detected by ^1^H NMR and HRMS, as outlined in the Supporting Information (section 2).

[ref23] Young C. M.; Elmi A.; Pascoe D. J.; Morris R. K.; McLaughlin C.; Woods A. M.; Frost A. B.; Houpliere A.; Ling K. B.; Smith T. K.; Slawin A. M. Z.; Willoughby P. H.; Cockroft S. L.; Smith A. D. The Importance of 1,5-Oxygen···Chalcogen Interactions in Enantioselective Isochalcogenourea Catalysis. Angew. Chem., Int. Ed. 2020, 59, 3705–3710. 10.1002/anie.201914421. 31856373

[ref24] This, together with the lower e.r. with higher catalyst loadings, points against a process where a dynamic kinetic resolution (DKR) of intermediate **II** may be dominant.

