# Supplementary material for: Enantioselective α-Chlorination Reactions of in Situ Generated C1 Ammonium Enolates under Base-Free Conditions
Source: Org Lett. 2021 Jul 28;23(15):6143–7. doi: 10.1021/acs.orglett.1c02256 (PMC8353620; doi:10.1021/acs.orglett.1c02256)
Supplement: Supplementary file 1 — ol1c02256_si_001.pdf [file ol1c02256_si_001.pdf]

SUPPORTING INFORMATION

Enantioselective α-Chlorination Reactions of in situ Generated C1 Ammonium Enolates under Base-Free Conditions

Lotte Stockhammer[a], David Weinzierl[a,], ThomasBögl[b] and Mario Waser[a*]

a) Institute of Organic Chemistry, Johannes Kepler University Linz, Altenbergerstraße 69, 4040 Linz, Austria.

Fax: +43 732 2468 5411; Tel: +43 732 2468 5402;

E-mail: mario.waser@jku.at

b) Institute of Analytical Chemistry, Johannes Kepler University Linz, Altenbergerstraße 69, 4040 Linz, Austria.

1. General Information: 1
2. NMR and HRMS Monitoring of the Chlorination Reaction: 2
3. Synthesis and Characterisation data of *pfp* ester 3c 4
4. α-Chlorination of Activated Aryl Esters 1: 5
5. Copies of NMR Spectra of Chlorination Products: 12
6. Copies of HPLC Chromatograms: 30

1. General Information:

NMR spectra were recorded on a Bruker Avance III 300 MHz spectrometer with a broad band observe probe and a sample changer for 16 samples, on a Bruker Avance DRX 500 MHz spectrometer, and on a Bruker Avance III 700 MHz spectrometer with an Ascend magnet and TCI cryoprobe which are property to the Austro Czech NMR Research Center "RERI uasb". All NMR spectra were referenced on the solvent residual peak ($CDCl_3$: δ 7.26 ppm for ^{1}H NMR and δ 77.16 ppm for ^{13}C NMR). NMR data are reported as follows: chemical shift (δ ppm), multiplicity (s = singlet, d = doublet, t = triplet, q = quartet, m = multiplet, br = broad), coupling constants (Hz) and integrals. High resolution mass spectra were obtained using an Thermo Fisher Scientific LTQ Orbitrap XL hybrid FT mass spectrometer with an ESI source and an Agilent G1607A coaxial sprayer and an Agilent QTOF 6520 with ESI source. EI MS analysis was done with a Shimadzu GC-MS QP-2020 using He as carrier gas. Optical rotations were measured on a Schmidt+Haensch Unipol L 100 polarimeter ($[\alpha]_D$ values are listed in $deg\cdot cm^3\cdot g^{-1}\cdot dm^{-1}$; concentration *c* is given in g/100 mL). Preparative column chromatography was carried out using Davisil LC 60A 70–200 MICRON silica gel. Thin layer chromatography was performed on Macherey-Nagel pre-coated TLC plates (silica gel, 60 F_{254}, 0.20 mm, ALUGRAM® Xtra SIL). TLC plates were visualized under 254 nm UV lamp. Enantiomeric ratios (*e.r.*) were determined by HPLC analysis using a Dionex Summit HPLC system with a CHIRALCEL OJ-H (4.6 × 250 mm, 5 µm), CHIRAL ART Amylose-SA (4.6 mm × 250 mm, 5 µm) and CHIRAL ART Cellulose-SB (4.6 mm × 250 mm, 5 µm) chiral stationary phase. All chemicals were purchased from commercial suppliers and used without further purification unless otherwise stated. All aryl ester starting materials **1** and **Se-ITU1** were synthesized according to recently reported protocols.[1, 2] NMR data of all esters matched those reported in literature (**1a**, **1b**, **1d**, **1g** and **1m**[3]; **1e**, **1f** and **1h**[4]; **1i**[5]; **1k**[6]; **1j** and **1l**[7]). Ester **1c** has been used before[8], but to the best of our knowledge no ^{1}H- and ^{13}C-NMR spectra thereof have been reported yet (these details can be found in section 3).

As it was observed previously[9], ionization of chlorinated esters **3** proofed to be problematic using an ESI source. Hence, (low resolution) EI ionization was performed to confirm product formation of the methyl esters via mass spectrometry. HRMS data given for products **3** in the following section refer to the corresponding morpholine amides **3**$^{\mathbf{Morpholine}}$ (obtained by quenching the reaction mixture with morpholine), as those compounds were detectable with electrospray ionization.

---

[1] McLaughlin, C.; Slawin, A. M. Z.; Smith, A. D. *Angew. Chem. Int. Ed.* **2019**, *58*, 15111–15119.
[2] Young, C. M.; Elmi, A.; Pascoe, D. J.; Morris, R. K.; McLaughlin, C.; Woods, A. M.; Frost, A. B.; La Houpliere, A. de; Ling, K. B.; Smith, T. K.; Slawin, A. M. Z.; Willoughby, P. H.; Cockroft, S. L.; Smith, A. D. *Angew. Chem. Int. Ed.* **2020**, *59*, 3705–3710.
[3] Schwarz, K. J.; Amos, J. L.; Klein, J. C.; Do, D. T.; Snaddon, T. N. *Journal of the American Chemical Society* **2016**, *138* (*16*), 5214–5217
[4] Jiang, X.; Beiger, J. J.; Hartwig, J. F. *Journal of the American Chemical Society* **2017**, *139* (*1*), 87–90
[5] Zhang, W.; Ready, J. M. *Angewandte Chemie (International ed. in English)* **2014**, *53* (*34*), 8980–8984
[6] Gui Ning, L.; Wang, S.; Feng Hu, X.; Ming Li, C.; Qun Xu, L. *Journal of materials chemistry. B* **2017**, *5* (*44*), 8814–8820
[7] Zhao, F.; Shu, C.; Young, C. M.; Carpenter-Warren, C.; Slawin, A. M. Z.; Smith, A. D. *Angewandte Chemie (International ed. in English)* **2021**, *60* (*21*), 11892–11900
[8] Hutchings-Goetz, L.; Yang, C.; Snaddon, T. N. *ACS catalysis* **2018**, *8* (*11*), 10537–10544
[9] Ventura, S. P. M.; Gurbisz, M.; Ghavre, M.; Ferreira, F. M. M.; Gonçalves, F.; Beadham, I.; Quilty, B.; Coutinho, J. A. P.; Gathergood, N. *ACS Sustainable Chem. Eng.* **2013**, *1*, 393–402.

2. NMR and HRMS Monitoring of the Chlorination Reaction:

HRMS monitoring

In step 1, the achiral catalyst **ITU4** (20 mol%) was added to a solution of aryl ester **1a^{pNP}** (0.1 mmol) in 1 mL THF. After this, a small aliquot was removed and analysed by HRMS. The formation of intermediate **I^{ITU4}** was visible while also free **ITU4** was present. Next, NCS (0.2 mmol) was added and again a small aliquot was removed and analysed. Satisfyingly, the formation of chlorinated intermediate **II^{ITU4}** could be observed.

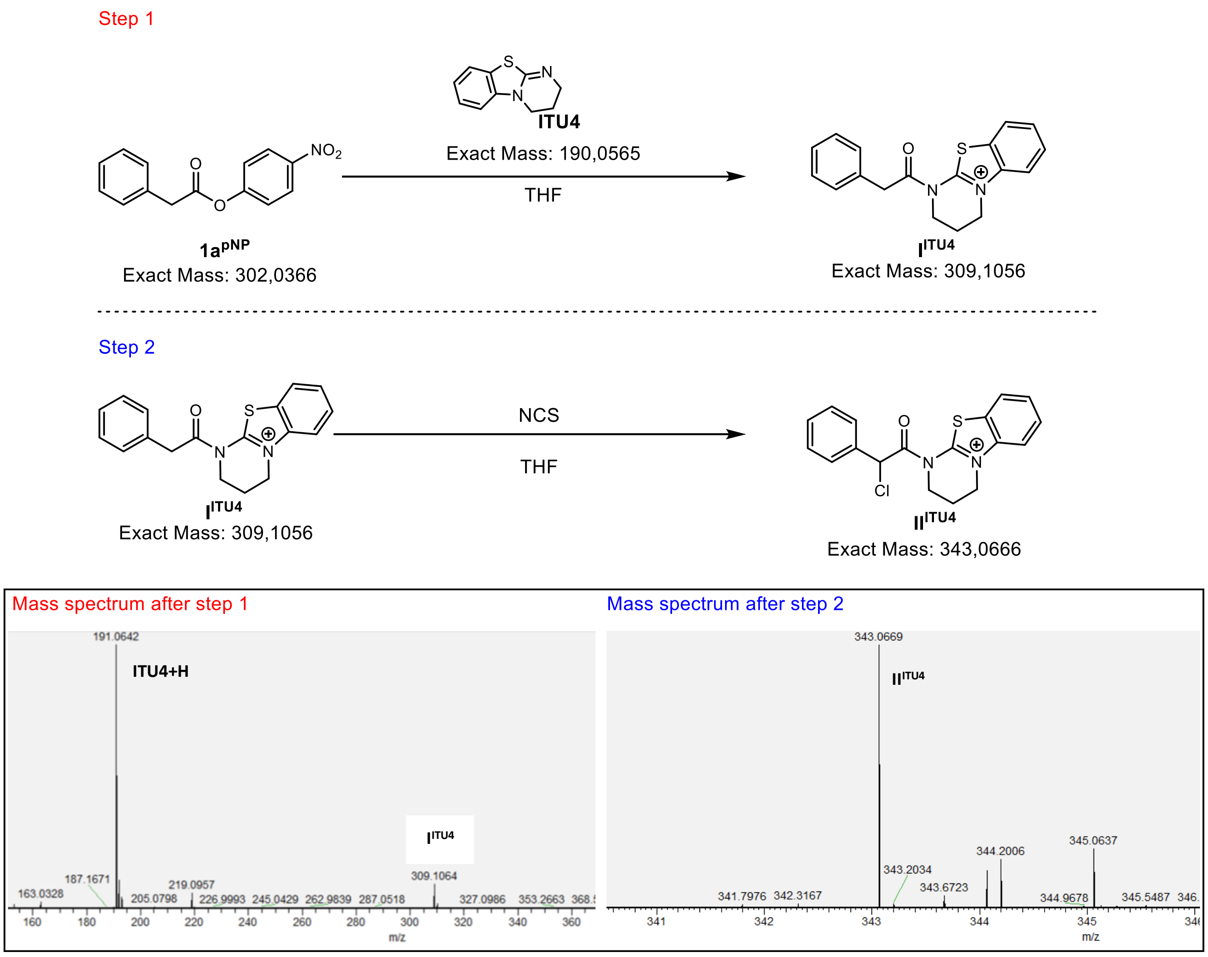

Figure 1: HRMS monitoring of the chlorination reaction.

NMR monitoring

The same reaction was also analysed with ^{1}H-NMR spectroscopy. First, an ^{1}H-NMR spectrum of an equimolar mixture of ester **1a^{pNP}** together with **ITU4** in $CDCl_3$ was recorded. Here, not too much of a difference to the spectra of the pure compounds was visible (also see figure 3, blue, red and green spectra). However, upon closer examination the formation of some free *p*-NO_2 phenol was observable (two doublets with J = 9.1 Hz at 8.1 and 6.9 ppm, figure 2).

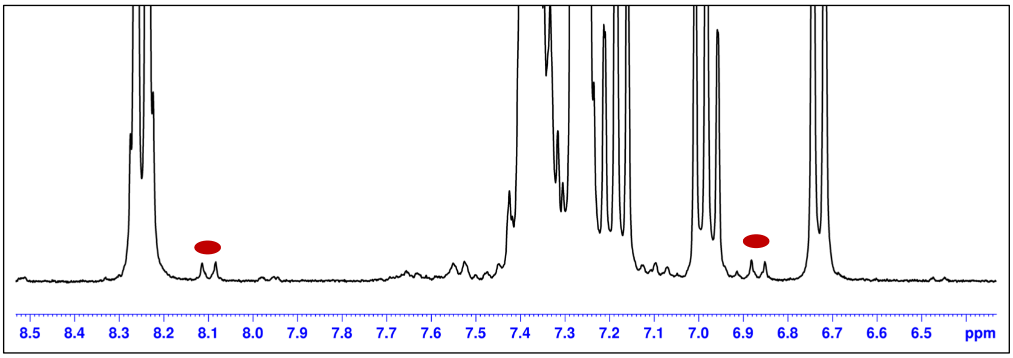

Figure 2: Aromatic region of the spectrum of ester **1a**^{pNP} together with **ITU4**. The two doublets at 8.1 und 6.9 ppm correspond to free nitrophenol formed by addition of **ITU4** to **1a**^{pNP}.

In a next step, 1 eq. of NCS was added to the NMR tube and another ^{1}H-spectrum was recorded (figure 3, purple spectrum). First, a clear low field shift of the **ITU4** signals is visible, indicating N-acylation of the catalyst (addition of **ITU4** to **1a**). In addition, when compared to the spectrum of pure chlorinated ester **1a**^{pNP} (figure 3, black spectrum), immediate α-chlorination could be detected. Characteristic therefore is the disappearance of the -CH_2 signal at around 3.9 ppm and the appearance of the -CH signal at around 5.6 ppm.

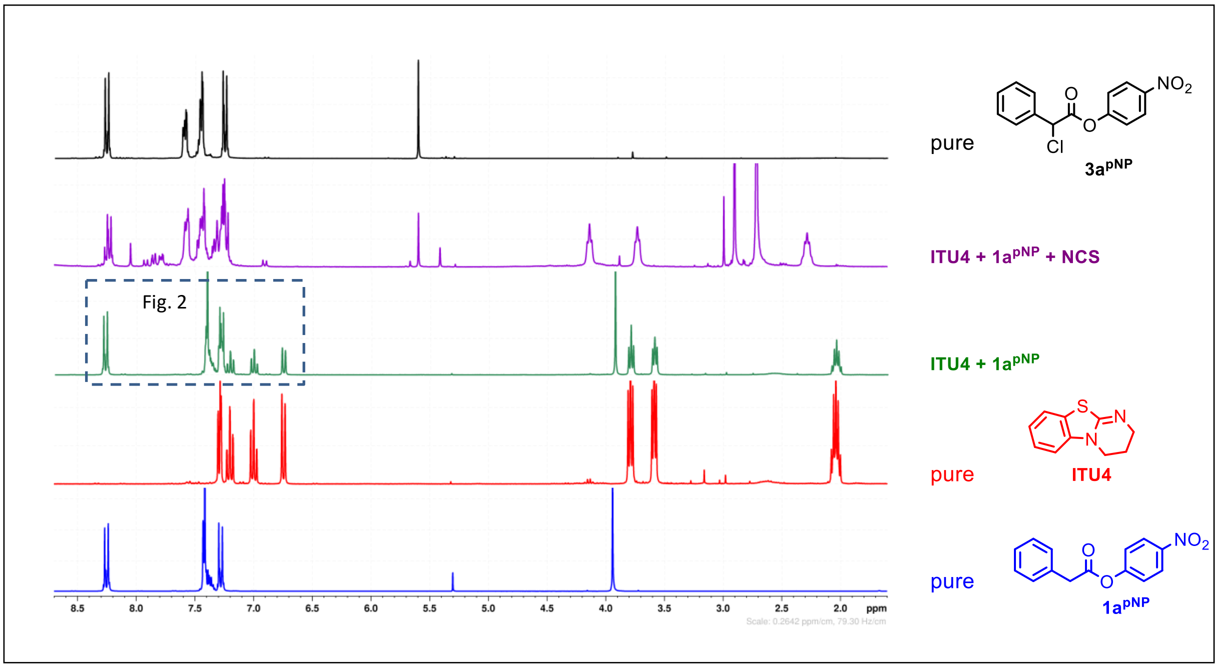

Figure 3: NMR spectra of the pure compounds **1a**^{pNP} (blue) and **ITU4** (red), the mixture of both (green), the mixture with additional NCS (purple) and the pure product **3a**^{pNP} (black).

3. Synthesis and Characterisation data of *pfp* ester 1c:

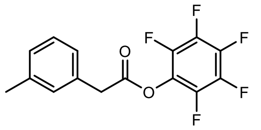

1c

Ester **3c** was prepared according to literature.[1] 2-(*m*-tolyl)acetic acid (1.00 g, 6.7 mmol) and EDC · HCl (1.67 g, 8.7 mmol) were dissolved in anhydrous DCM and stirred for 10 min at r.t. Then, pentafluoro phenol (1.86 g, 10.1 mmol) was added and the mixture was further stirred for 16 h at r.t. Then, water was added, the phases were separated, and the aqueous phase was washed with DCM trice. The combined organic layers were dried over Na_2SO_4, filtered, and concentrated on the rotary evaporator. Purification by column chromatography on silica using DCM as eluent gave ester **1c** in a yield of 1.52 g (74%).

^{1}H-NMR (300 MHz, $CDCl_3$, 298 K) δ / ppm = 7.30-7.25 (m, 1 H), 7.17-7.14 (m, 3 H), 3.94 (s, 2 H), 2.38 (s, 3 H). **^{13}C-NMR** (75 MHz, $CDCl_3$, 298 K) δ / ppm = 167.4, 138.6, 131.8, 129.9, 128.7, 128.5, 126.2, 40.4, 21.7 **^{19}F-NMR** (282 MHz, $CDCl_3$, 298 K) δ / ppm = -152.6 (2 F), -157.9, -162.4 (2 F).

4. α-Chlorination of Activated Aryl Esters 1:

General procedure for the α-chlorination of aryl esters followed by an immediate alcohol quench (0.1 mmol scale)

The respective *pfp*-Ester **1** (0.1 mmol) and benzotetramisole (BTM, **ITU3**; 2.5 mg, 10 mol%) were dissolved in 1 mL THF in a cooling Schlenk flask and cooled to -60 °C. Then, NCS (**2**; 26.7 mg, 0.2 mmol) was added, and the mixture was stirred at -60 °C for 63 h. After this period, 2 mL of the respective alcohol (MeOH, EtOH or *i*PrOH) were added at -60 °C. Then, the circulation chiller was turned off and the mixture was allowed to slowly warm to r.t. and stirred for a total of 8 h. It was filtered over Na_2SO_4, washed with DCM and evaporated to dryness. The crude products were purified by column chromatography on silica (heptanes - heptanes/DCM 1/3).

1 mmol scale procedure for the synthesis of 3d^{OMe}

Ester **1d** (316 mg, 1.0 mmol) and BTM (**ITU3**; 25.0 mg, 10 mol%) were dissolved in 10 mL THF in a cooling Schlenk flask and cooled to -60 °C. Then, NCS (**2**; 267 mg, 2.0 mmol) was added, and the mixture was stirred at -60 °C for 63 h. After this, 20 mL pre-cooled MeOH (at -60 °C, using an acetone/N_2 bath) were slowly added with syringe. The mixture was further stirred at -60 °C for 1 h, then the circulation chiller was turned off, the mixture was slowly warmed to r.t. and further stirred at r.t. for 8 h. The mixture was filtered over Na_2SO_4, washed with DCM and evaporated to dryness. The crude product was purified by column chromatography on silica (heptanes - heptanes/DCM 1/3). Product ester **3d^{OMe}** was obtained in a yield of 142 mg (71%).

General procedure for the α-chlorination of aryl esters followed by an amine quench (0.1 mmol scale)

Pfp ester **1a** (30.2 mg, 0.1 mmol) and BTM (**ITU3**; 2.5 mg, 10 mol%) were dissolved in 2 mL THF in a cooling Schlenk flask and cooled to -60 °C. Then, NCS (**2**; 26.7 mg, 0.2 mmol) was added, and the mixture was stirred for 63 h. After this period, the amine (benzylamine or morpholine, 10 eq) was added at -60 °C. After this, the circulation chiller was turned off and the mixture further stirred for 8 h at r.t. 1 mL $NaHCO_3$ was added, the phases separated, and the aqueous phase extracted with Et_2O trice. The combined organic layers were washed with brine, dried over Na_2SO_4, and evaporated to dryness. The crude products were purified by column chromatography on silica using heptanes/EtOAc 2/1.

Racemic samples of the chlorinated methyl esters for HPLC analysis were prepared using achiral **ITU4** instead of BTM. Racemic samples of the other esters and amides were obtained from commercial α-chloro phenylacetyl chloride following reported procedures.[10,11,12]

Characterization data for α-chlorinated methyl esters

Methyl 2-chloro-2-phenylacetate 3a^{OMe}

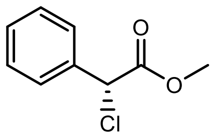

3a^{OMe}

Chlorinated ester *(R)*-**3a**^{OMe} was obtained as a colourless oil in a yield of 16.8 mg (91%). ***er*** = 99:1. TLC (DCM/Heptanes 3/1): R_f= 0.52 (UV), $[\alpha]_D^{23}$ = -68.4 (*c* 1.0, $CHCl_3$, 99:1 *e.r.*), Analytical data are in accordance with literature.[10] **^{1}H-NMR** (300 MHz, $CDCl_3$, 298 K) δ / ppm = 7.51-7.48 (m, 2 H), 7.39-7.37 (m, 3 H), 5.37 (s, 1 H), 3.78 (s, 1 H). **^{13}C-NMR** (75 MHz, $CDCl_3$, 298 K) δ / ppm = 169.0, 135.9, 129.5, 129.0 (2 C), 128.1 (2 C), 59.1, 53.5. **EI-MS**: *m/z* Calcd for $C_9H_9ClO_2^{+\cdot}$ 184; Found 184 (15, $M^{+\cdot}$), 125 (100, $[M – COOMe]^+$). **HRMS** (ESI-TOF) of the corresponding morpholine amide **3a**^{Morpholine} *m/z*: $[M+H]^+$ Calcd for $C_{12}H_{15}ClNO_2^+$ 240.0786; Found 240.0784. **HPLC** (YMC-SB, *n*-hexane/IPA = 250/1, flow rate = 1.0 mL min^{-1}, *I* = 220 nm): t_r = 10.1 min *(major)*, 11.6 min *(minor)*.

Methyl 2-chloro-2-(o-tolyl)acetate 3b^{OMe}

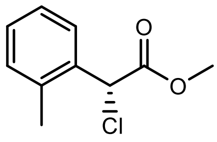

3b^{OMe}

Chlorinated ester *(R)*-**3b**^{OMe} was obtained as a yellow oil in a yield of 11.9 mg (60%). ***er*** = 98:2. TLC (DCM/Heptanes 3/1): R_f= 0.58 (UV), $[\alpha]_D^{23}$ = -29.0 (*c* 1.0, $CHCl_3$, 98:2 *e.r.*), **^{1}H-NMR** (300 MHz, $CDCl_3$, 298 K) δ / ppm = 7.29-7.17 (m, 4 H), 5.33 (s, 1 H), 3.78 (s, 3 H), 2.37 (s, 3 H), **^{13}C-NMR** (75 MHz, $CDCl_3$, 298 K) δ / ppm = 169.1, 138.9, 135.8, 130.3, 128.9, 128.6, 125.2, 59.2, 53.5, 21.5. **EI-MS**: *m/z* Calcd for $C_{10}H_{11}ClO_2^{+\cdot}$ 198; Found 198 (11, $M^{+\cdot}$), 139 (100, $[M – COOMe]^+$). **HRMS** (ESI-TOF) of the corresponding morpholine amide **3b**^{Morpholine} *m/z:* $[M+H]^+$ Calcd for $C_{13}H_{17}ClNO_2^+$ 254.0942; Found 254.0942. **HPLC** (YMC-SB *n*-hexane/IPA = 250/1, flow rate = 0.5 mL min^{-1}, *I* = 220 nm): t_r = 24.0 min *(major),* 25.7 min *(minor).*

Methyl 2-chloro-2-(m-tolyl)acetate 3c^{OMe}

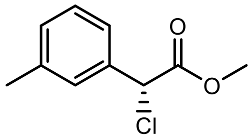

3c^{OMe}

Chlorinated ester *(R)*-**3c**^{OMe} was obtained as a yellow oil in a yield of 12.9 mg (65%). ***er*** = 99:1. TLC (DCM/Heptanes 3/1): R_f= 0.58 (UV), $[\alpha]_D^{23}$ = -71.8 (*c* 1.0, $CHCl_3$, 99:1 *e.r.*), **^{1}H-NMR** (300 MHz, $CDCl_3$, 298 K) δ / ppm = 7.29-7.26 (m, 3 H), 7.19-7.17 (m, 1 H), 5.33 (s, 1 H), 3.78 (s, 3 H), 2.37 (s, 3 H), **^{13}C-NMR** (75 MHz, $CDCl_3$, 298 K) δ / ppm = 169.1, 138.9, 135.8, 130.3, 128.9, 128.6, 125.1, 59.2, 53.5, 21.5. **EI-MS**: *m/z* Calcd for $C_{10}H_{11}ClO_2^{+\cdot}$ 198; Found: 198 (16, $M^{+\cdot}$), 139 (100, $[M – COOMe]^+$). **HRMS** (ESI-TOF) of the corresponding morpholine amide

[10] Haughton, L.; Williams, J. M. J. *Synthesis* **2001**, 943–946.
[11] Peczkowski, G. R.; Craven, P. G. E.; Stead, D.; Simpkins, N. S. *Chem. Commun.* **2019**, *55*, 4214–4217.
[12] Otevrel, J.; Svestka, D.; Bobal, P. *RSC Adv.* **2020**, *10*, 25029–25045.

3c$^{\mathbf{Morpholine}}$ *m/z*: $[M+H]^+$ Calcd. for $C_{13}H_{17}ClNO_2^+$ 254.0942; Found 254.0945. **HPLC** (YMC-SB, *n*-hexane/IPA = 250/1, flow rate = 1.0 mL min^{-1}, I = 220 nm): t_r = 9.4 min *(major)*, 11.3 min *(minor).*

Methyl 2-chloro-2-(p-tolyl)acetate 3d$^{\mathbf{OMe}}$

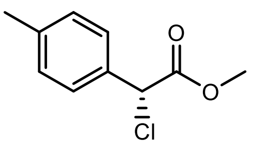

3d$^{\mathbf{OMe}}$

Chlorinated ester *(R)*-**3d**$^{\mathbf{OMe}}$ was obtained as a yellow oil in yields of 14.3 mg (72%, 0.1 mmol scale) and 142 mg (71%, 1 mmol scale) with ***er*** = 99:1 in both cases. TLC (DCM/Heptanes 3/1): R_f= 0.54 (UV), $[\alpha]_D^{23}$ = -55.2 (*c* 1.0, $CHCl_3$, 99:1 *e.r.*), **^{1}H-NMR** (300 MHz, $CDCl_3$, 298 K) δ / ppm = 7.38 (d, J = 8.2 Hz, 2 H), 7.19 (d, J = 8.2 Hz, 2 H), 5.34 (s, 1 H), 3.77 (s, 3 H), 2.36 (s, 3 H). **^{13}C-NMR** (75 MHz, $CDCl_3$, 298 K) δ / ppm = 169.1, 139.5, 132.9, 129.7 (2 C), 127.9 (2 C), 59.0, 53.4, 21.3. **EI-MS**: *m/z* Calcd for $C_{10}H_{11}ClO_2^{+\cdot}$ 198; Found 198 (14, $M^{+\cdot}$), 139 (100, $[M - COOMe]^+$). **HRMS** (ESI-TOF) of the corresponding morpholine amide **3d**$^{\mathbf{Morpholine}}$ *m/z*: $[M+H]^+$ Calcd. for $C_{13}H_{17}ClNO_2^+$ 254.0942; Found: 254.0940. **HPLC** (YMC-SB, *n*-hexane/IPA = 250/1, flow rate = 1.0 mL min^{-1}, I = 240 nm): t_r = 9.6 min *(major)*, 10.8 min *(minor).*

Methyl 2-chloro-2-(2-fluorophenyl)acetate 3e$^{\mathbf{OMe}}$

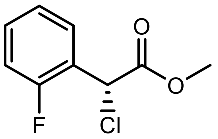

3e$^{\mathbf{OMe}}$

Chlorinated ester *(R)*-**3e**$^{\mathbf{OMe}}$ was obtained as a colourless oil in a yield of 14.8 mg (73%). ***er*** = 99:1. TLC (DCM/Heptanes 3/1): R_f= 0.48 (UV), $[\alpha]_D^{23}$ = -28.7 (*c* 1.0, $CHCl_3$, 99:1 *e.r.*), **^{1}H-NMR** (300 MHz, $CDCl_3$, 298 K) δ / ppm =7.58 (td, J_1 = 7.6 Hz, J_2 = 1.7 Hz, 1 H), 7.37-7.32 (m, 1H), 7.22-7.06 (m, 2 H), 5.72 (s, 1 H), 3.80 (s, 3 H). **^{13}C-NMR** (75 MHz, $CDCl_3$, 298 K) δ / ppm = 168.3, 159.8 (d, J = 249.7 Hz, 1 C), 131.2 (d, J = 131.2, 1 C), 129.7 (d, J = 2.3 Hz, 1 C), 124.8 (d, J = 3.4 Hz, 1 C), 123.6 (d, J = 13.8 Hz, 1 C), 115.8 (d, J = 21.5 Hz, 1 C), 53.6, 51.8 (d, J = 4.6 Hz, 1 C). **EI-MS**: *m/z* Calcd for $C_9H_8ClFO_2^{+\cdot}$ 202; Found 202 (13, $M^{+\cdot}$), 143 (100, $[M - COOMe]^+$). **HRMS** (ESI-TOF) of the corresponding morpholine amide **3e**$^{\mathbf{Morpholine}}$ *m/z:* $[M+H]^+$ Calcd for $C_{12}H_{14}ClFNO_2^+$ 258.0692; Found 258.0690. **HPLC** (YMC-SA, *n*-hexane/IPA = 250/1, flow rate = 1.0 mL min^{-1}, I = 220 nm): t_r 5.8 min *(minor)*, 7.25 min *(major).*

Methyl 2-chloro-2-(3-chlorophenyl)acetate 3f$^{\mathbf{OMe}}$

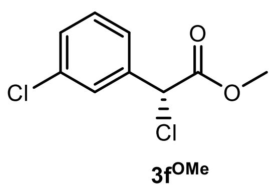

3f$^{\mathbf{OMe}}$

Chlorinated ester *(R)*-**3f**$^{\mathbf{OMe}}$ was obtained as a colourless oil in a yield of 16.0 mg (73%). ***er*** = 97:3. TLC (DCM/Heptanes 3/1): R_f= 0.56 (UV), $[\alpha]_D^{23}$ = -19.7 (*c* 1.0, $CHCl_3$, 97:3 *e.r.*), **^{1}H-NMR** (300 MHz, $CDCl_3$, 298 K) δ / ppm = 7.50 (br s, 1 H), 7.49-7.31 (m, 3 H), 5.31 (s, 1 H), 3.79 (s, 3 H).). **^{13}C-NMR** (75 MHz, $CDCl_3$, 298 K) δ / ppm = 168.4, 137.5, 134.8, 130.2, 129.6, 128.2, 126.2, 58.1, 53.6. **EI-MS** *m/z:* Calcd for $C_9H_8Cl_2O_2^{+\cdot}$ 218; Found 218 (18, $M^{+\cdot}$), 159 (100, $[M - COOMe]^+$). **HRMS** (ESI-TOF) of the corresponding morpholine amide **3f**$^{\mathbf{Morpholine}}$ *m/z:* $[M+H]^+$: Calcd for $C_{12}H_{14}Cl_2NO_2^+$ 274.0396; Found 274.0395. **HPLC** (YMC-SB, *n*-hexane/IPA = 250/1, flow rate = 0.5 mL min^{-1}, I = 220 nm): t_r = 22.2 min *(major),* 23.1 min *(minor).*

Methyl 2-chloro-2-(4-chlorophenyl)acetate 3g^{OMe}

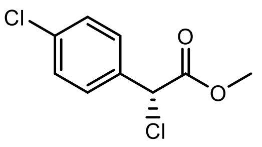

3g^{OMe}

Chlorinated ester *(R)*-**3g^{OMe}** was obtained as a yellow oil in a yield of 16.2 mg (74%). ***er*** = 98:2. TLC (DCM/Heptanes 3/1): R_f= 0.54 (UV), $[\alpha]_D^{23}$ = -38.4 (*c* 1.0, $CHCl_3$, 98:2 *e.r.*), **^{1}H-NMR** (300 MHz, $CDCl_3$, 298 K) δ / ppm = 7.44 (d, J = 8.6 Hz, 2 H), 7.36 (d, J = 8.6 Hz, 2 H), 5.32 (s, 1 H), 3.78 (s, 1 H). **^{13}C-NMR** (75 MHz, $CDCl_3$, 298 K) δ / ppm = 168.6, 135.5, 134.3, 129.4 (2 C), 129.2 (2 C), 58.2, 53.6. **EI-MS** *m/z:* Calcd for $C_9H_8Cl_2O_2^{+\cdot}$ 218; found 218 (15, $M^{+\cdot}$), 159 (100, $[M - COOMe]^+$). **HRMS** (ESI-TOF) of the corresponding morpholine amide **3g^{Morpholine}** *m/z:* $[M+H]^+$ Calcd. for $C_{12}H_{14}Cl_2NO_2^+$ 274.0396; Found 274.0395. **HPLC** (CHIRALCEL OJ-H, *n*-hexane/IPA = 80/1, flow rate = 1.0 mL min^{-1}, I = 240 nm): t_r = 21.9 min *(major)*, 24.1 min *(minor)*.

Methyl 2-chloro-2-(4-bromophenyl)acetate 3h^{OMe}

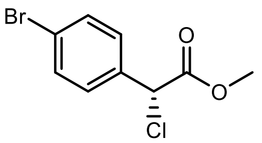

3h^{OMe}

Chlorinated ester *(R)*-**3h^{OMe}** was obtained as a yellow oil in a yield of 21.6 mg (79%) (after 40 h). ***er*** = 98:2. TLC (DCM/Heptanes 3/1): R_f= 0.55 (UV), $[\alpha]_D^{23}$ = -75.9 (*c* 1.0, $CHCl_3$, 98:2 *e.r.*), **^{1}H-NMR** (300 MHz, $CDCl_3$, 298 K) δ / ppm = 7.52 (d, J = 8.5 Hz, 2 H), 7.37 (d, J = 8.5 Hz, 2 H), 5.31 (s, 1 H), 3.78 (s, 3 H). **^{13}C-NMR** (75 MHz, $CDCl_3$, 298 K) δ / ppm = 168.6, 134.9, 132.2 (2 C), 129.8 (2 C), 123.8, 58.3, 53.6. **EI-MS** *m/z:* Calcd for $C_9H_8ClBrO_2^{+\cdot}$ 262; Found 262 (10, $M^{+\cdot}$), 203 (100, $[M - COOMe]^+$). **HRMS** (ESI-TOF) of the corresponding morpholine amide **3h^{Morpholine}** *m/z*: $[M+H]^+$ Calcd for $C_{12}H_{14}ClBrNO_2^+$ 317.9891; Found 317.9901. **HPLC** (CHIRALCEL OJ-H, *n*-hexane/IPA = 80/1, flow rate = 1.0 mL min^{-1}, I = 240 nm): t_r = 21.4 min *(major)*, 24.4 min *(minor)*.

Methyl -2-([1,1'-biphenyl]-4-yl)-2-chloroacetate 3i^{OMe}

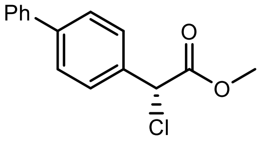

3i^{OMe}

Chlorinated ester *(R)*-**3i^{OMe}** was obtained as a yellow solid in a yield of 23.8 mg (95%). ***er*** = 98:2. TLC (DCM/Heptanes 3/1): R_f= 0.44 (UV), $[\alpha]_D^{23}$ = 63.7 (*c* 1.0, $CHCl_3$, 98:2 *e.r.*), **^{1}H-NMR** (300 MHz, $CDCl_3$, 298 K) δ / ppm = 7.63-7.55 (m, 6 H), 7.48-7.43 (m, 3 H), 5.42 (s, 1 H), 3.81 (s, 1 H). **^{13}C-NMR** (75 MHz, $CDCl_3$, 298 K) δ / ppm = 169.0, 142.5, 140.3, 134.7, 129.0 (2 C), 128.5 (2 C), 127.9, 127.8 (2 C), 127.3 (2 C), 58.9, 53.5. **EI-MS** *m/z:* Calcd for $C_{15}H_{13}ClO_2^{+\cdot}$ 260; Found 260 (30, $M^{+\cdot}$), 201 (100, $[M - COOMe]^+$). **HRMS** (ESI-TOF) of the corresponding morpholine amide **3i^{Morpholine}** *m/z*: $[M+H]^+$ Calcd for $C_{18}H_{19}ClNO_2^+$ 316.1099; Found 316.1095. **HPLC** (CHIRALCEL OJ-H, *n*-hexane/IPA = 20/1, flow rate = 1.0 mL min^{-1}, I = 220 nm): t_r= 33.6 min *(major)*, 43.6 min *(minor)*.

Methyl 2-chloro-2-(naphthalen-1-yl)acetate 3j^{OMe}

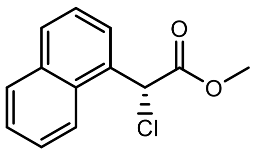

3j^{OMe}

Chlorinated ester *(R)*-**3i^{OMe}** was obtained as a colourless oil in a yield of 11.8 mg (50%). ***er*** = 98:2. TLC (DCM/Heptanes 3/1): R_f= 0.62 (UV), $[\alpha]_D^{23}$ = -95.2 (*c* 1.0, $CHCl_3$, 98:2 *e.r.*), **^{1}H-NMR** (300 MHz, $CDCl_3$, 298 K) δ / ppm = 8.13 (d, *J* = 8.5 Hz, 1 H), 7.92-7.87 (m, 2 H), 7.70-7.45 (m, 4 H), 6.12 (s, 1 H), 3.78 (s, 3 H).). **^{13}C-NMR** (75 MHz, $CDCl_3$, 298 K) δ / ppm = 169.3, 134.1, 131.7, 130.5, 130.4, 129.2, 127.2, 127.1, 126.3, 125.5, 123.1, 57.3, 53.6. **EI-MS** *m/z:* Calcd for $C_{13}H_{11}ClO_2^{+\cdot}$ 234; Found 234 (23, $M^{+\cdot}$), 175 (100, $[M-COOMe]^+$). **HRMS** (ESI-TOF) of the corresponding morpholine amide **3j^{Morpholine}** *m/z*: $[M+H]^+$ Calcd for $C_{16}H_{17}ClNO_2^+$ 290.0942; Found 316.0937. **HPLC** (CHIRALCEL OJ-H, *n*-hexane/IPA = 4/1, flow rate = 1.0 mL min^{-1}, *I* = 220 nm): t_r = 24.6 min *(major)*, 33.2 min *(minor)*.

Methyl 2-chloro-2-(thiophen-3-yl)acetate 3k^{OMe}

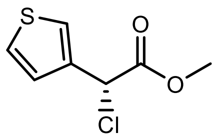

3k^{OMe}

Chlorinated ester *(R)*-**3k^{OMe}** was obtained as a colourless oil in a yield of 15.7 mg (82%). ***er*** = 93:7. TLC (DCM/Heptanes 3/1): R_f= 0.50 (UV), $[\alpha]_D^{23}$ = -39.6 (*c* 1.0, $CHCl_3$, 93:7 *e.r.*), **^{1}H-NMR** (300 MHz, $CDCl_3$, 298 K) δ / ppm = 7.43 (dd, J_1 = 3.0 Hz, J_2 = 1.2 Hz, 1H), 7.33 (dd, J_1 = 5.1 Hz, J_2 = 3.0 Hz, 1 H), 7.22 (dd, J_1 = 5.1 Hz, J_2 = 1.2 Hz), 5.46 (s, 1 H), 3.80 (s, 3 H). **^{13}C-NMR** (75 MHz, $CDCl_3$, 298 K) δ / ppm = 168.7, 135.9, 127.1, 127.0, 125.1, 54.2, 53.5. **EI-MS** *m/z:* Calcd for $C_7H_7ClO_2S^{+\cdot}$: 190; Found 190 (16, $M^{+\cdot}$), 131 (100, $[M-COOMe]^+$). **HPLC** (CHIRALCEL OJ-H, *n*-hexane/IPA = 20/1, flow rate = 1.0 mL min^{-1}, *I* = 220 nm): t_r = 29.6 min *(major)*, 34.4 min *(minor)*.

Methyl 2-chloro-2-(4-methoxyphenyl)acetate 3m^{OMe}

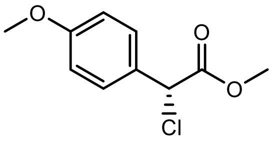

3m^{OMe}

Pfp-Ester **1m** (33.3 mg, 0.1 mmol) and BTM (**ITU3**; 2.5 mg, 10 mol%) were dissolved in 1 mL THF in a cooling Schlenk flask and cooled to -60 °C. Then, NCS (**2**; 26.7 mg, 0.2 mmol) and 8 µL MeOH (0.2 mmol) were added, and the mixture was stirred at -60 °C for 63 h. After this period, the circulation chiller was turned off and the mixture was warmed to r.t. slowly. It was filtered over Na_2SO_4, washed with some DCM, and purified by column chromatography (DCM/heptanes 1/1). Chlorinated ester *(R)*-**3m^{OMe}** was obtained in a yield of 18.2 mg (85%). ***er*** = 91:9. $[\alpha]_D^{23}$ = 41.9 (*c* 1.0, $CHCl_3$, 91:9 *e.r.*). TLC (DCM/Heptanes 3/1): R_f= 0.20 (UV), **^{1}H-NMR** (300 MHz, $CDCl_3$, 298 K) δ / ppm = 7.42 (d, *J* = 8.8 Hz, 2 H), 6.90 (d, *J* = 8.8 Hz, 2 H), 5.33 (s, 1 H), 3.81 (s, 3 H), 3.77 (s, 3 H). **^{13}C-NMR** (75 MHz, $CDCl_3$, 298 K) δ / ppm = 169.2, 160.5, 129.5 (2 C), 127.9, 114.4 (2 C), 58.8, 55.5, 53.4. **HPLC** (CHIRALCEL OJ-H, *n*-hexane/IPA = 20/1, flow rate = 0.5 mL min^{-1}, *I* = 220 nm): t_r = 17.3 min *(major)*, 17.9 min *(minor)*.

*MS measurements in MeOH solutions only showed the product **5m^{OMe}** where the α-Cl was replaced by α-OMe:* **EI-MS** *m/z:* Calcd for $C_{11}H_{14}ClO_4^{+\cdot}$ 210; Found 210 (2, $M^{+\cdot}$), 151 (100, $[M-COOMe]^+$). **HRMS** (ESI-TOF) of the corresponding α-MeO-morpholine amide *m/z*: $[M+H]^+$ Calcd for $C_{14}H_{20}ClNO_4^+$ 266.1387; Found 266.1382.

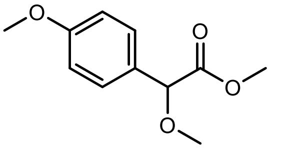

5m^{OMe}

Characterization of other products

Methyl 2,2-dichloro-2-(4-nitrophenyl)acetate 4l^{OMe}

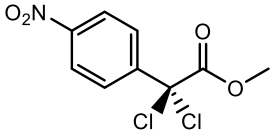

4l^{OMe}

This compound was the main product when attempting the enantioselective α-chlorination of *pfp*-(4-nitrophenyl)acetate **1l**. Dichlorinated ester **4l^{OMe}** was obtained as a colourless oil in a yield of 21.1 mg (80%). Spectral data are in accordance with literature.[13] ^{1}**H-NMR** (300 MHz, $CDCl_3$, 298 K) δ / ppm = 8.27 (d, J = 9.1 Hz, 2 H), 7.91 (d, J = 9.1 Hz, 2 H), 3.89 (s, 3 H). ^{13}**C-NMR** (75 MHz, $CDCl_3$, 298 K) δ / ppm = 165.7, 148.6, 144.9, 128.2, 123.7, 82.9, 55.4.

Perfluorophenyl 2-chloro-2-phenylacetate 3a^{pfp}

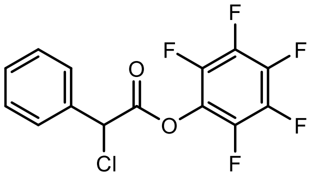

3a^{pfp}

By omitting the described alcohol/amine quench (*vide supra*) and using water followed by extractive workup instead, also chlorinated *pfp* ester **3a^{pfp}** could be obtained. However, this compound proofed to be difficult to isolate via column chromatography and rather unstable. The compound was not accessible via HRMS (ESI). Product formation was confirmed by the synthesis of **3a^{pfp}** from commercial α-chloro phenylacetyl chloride and comparison of NMR spectra.

^{1}**H-NMR** (300 MHz, $CDCl_3$, 298 K) δ / ppm = 7.59-7.56 (m, 2 H), 7.47-7.44 (m, 3 H), 5.70 (s, 1 H). ^{13}**C-NMR** (75 MHz, $CDCl_3$, 298 K) δ / ppm = 164.9, 134.4, 130.1, 129.4 (2 H), 128.1 (2 H), 58.2.

Characterization data for the targets obtained after alternative quenches:

Ethyl 2-chloro-2-phenylacetate 3a^{OEt}

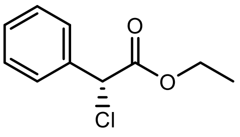

3a^{OEt}

Chlorinated ester *(R)*-**3a^{OEt}** was obtained as a slightly yellow oil in a yield of 15.4 mg (78%). ***er*** = 94:6. TLC (DCM/Heptanes 1/3): R_f = 0.62 (UV), $[\alpha]_D^{23}$ = -47.1 (*c* 1.0, $CHCl_3$, 94:6 *e.r.*). Spectral data were in accordance with literature.[14] ^{1}**H-NMR** (300 MHz, $CDCl_3$, 298 K) δ / ppm = 7.50-7.48 (m, 2 H), 7.39-7.36 (m, 3 H), 5.34 (s, 1 H), 4.24 (q, J = 7.1 Hz, 1 H), 4.21 (q, J = 7.1 Hz, 1 H), 1.26 (t, J = 7.1 Hz, 3 H). ^{13}**C-NMR** (75 MHz, $CDCl_3$, 298 K) δ / ppm = 168.5, 136.0, 129.4, 129.0, 128.0, 62.6, 59.2, 14.1. **EI-MS** *m/z:* Calcd for $C_{10}H_{11}ClO_2^{+\cdot}$: 198; Found 198 (5, $M^{+\cdot}$), 125 (100, $[M - COOEt]^+$). *As discussed for the methyl esters (vide supra), these compounds were not directly detectable by ESI-TOF HRMS. The morpholine quench would lead to morpholineamide 3a^{Morpholine} with the HRMS data outlined above.* **HPLC** (YMC-SB, *n*-hexane/IPA = 250/1, flow rate = 1.0 mL min^{-1}, I = 220 nm): t_r = 7.2 min *(major)*, 8.1 min *(minor).*

[13] Tao, J.; Tran, R.; Murphy, G. K. *Journal of the American Chemical Society* **2013**, *135* (*44*), 16312–16315
[14] Guieysse, D.; Cortés, J.; Puech-Guenot, S.; Barbe, S.; Lafaquière, V.; Monsan, P.; Siméon, T.; André, I.; Remaud-Siméon, M. *Chembiochem* **2008**, *9*, 1308–1317

Isopropyl 2-chloro-2-phenylacetate 3a^{OiPr}

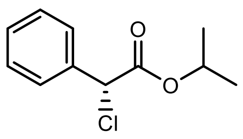

3a^{OiPr}

Chlorinated ester *(R)*-**3a**^{OiPr} was obtained as a slightly yellow oil in a yield of 17.0 mg (80%). ***er*** = 95:5, TLC (DCM/Heptanes 1/3): R_f= 0.80 (UV), $[\alpha]_D^{23}$ = -32.1 (*c* 1.0, $CHCl_3$, 95:5 *e.r.*). **^{1}H-NMR (**300 MHz, $CDCl_3$, 298 K) *δ* / ppm = 7.49-7.47 (m, 2 H), 7.38-7.36 (m, 3 H), 5.31 (s, 1 H), 5.06 (sept, *J* = 6.3 Hz, 1 H), 1.27 (d, *J* = 6.3 Hz, 3 H), 1.18 (d, *J* = 6.3 Hz). **^{13}C-NMR** (75 MHz, $CDCl_3$, 298 K) *δ* / ppm = 168.0, 136.1, 129.3, 128.9 (2 C), 128.0 (2 C), 70.5, 59.5, 21.7, 21.5. **EI-MS** *m/z:* Calcd for $C_{11}H_{13}ClO_2^{+\cdot}$: 212; Found 212 (0.5, $M^{+\cdot}$), 125 (50, $[M - COO\textit{i}Pr]^+$), 43 (100). *As discussed for the methyl esters (vide supra), these compounds were not directly detectable by ESI-TOF HRMS. The morpholine quench would lead to morpholineamide **3a**^{Morpholine} with the HRMS data outlined above.* **HPLC** (YMC-SB, *n*-hexane/IPA = 500/1, flow rate = 1.0 mL min^{-1}, *I* = 220 nm): t_r = 9.2 min *(major)*, 10.2 min *(minor)*.

N-Benzyl-2-chloro-2-phenylacetamide 3a^{NHBn}

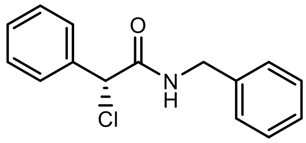

3a^{NHBn}

Chlorinated amide *(R)*-**3a**^{NHBn} was obtained as a yellow solid in a yield of 15.6 mg (60%). ***er*** = 71:29. TLC (DCM/Heptanes 1/3): R_f= 0.52 (UV), $[\alpha]_D^{23}$ = 14.8 (*c* 1.0, $CHCl_3$, 29:71 *e.r.*). Spectral data were in accordance with literature.[15] **^{1}H-NMR (**300 MHz, $CDCl_3$, 298 K) *δ* / ppm = 7.45-7.32 (m, 10 H), 7.04 (br. S, 1 H), 5.44 (s, 1 H), 4.51 (d, *J* = 5.8 Hz, 2 H). **^{13}C-NMR** (75 MHz, $CDCl_3$, 298 K) *δ* / ppm = 167.7, 137.4, 137.1, 129.3, 129.1 (2 C), 129.0 (2 C), 127.93, 127.91 (2C), 127.87 (2 C), 61.8, 44.3. **HRMS** (ESI-TOF) *m/z*: $[M+H]^+$ Calcd for $C_{15}H_{15}ClNO^+$ 260.0837; Found 260.0838. **HPLC** (CHIRALCEL OJ-H, *n*-hexane/IPA = 7/3, flow rate = 1.0 mL min^{-1}, *I* = 220 nm): t_r = 29.9 min, *t_r(minor)* = 32.3 min *(major)*.

2-Chloro-1-morpholino-2-phenylethan-1-one 3a^{Morpholine}

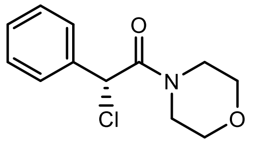

3a^{Morpholine}

Chlorinated amide *(R)*-**3a**^{Morpholine} was obtained as a yellow solid in a yield of 20.4 mg (85%). ***er*** = 81:19. TLC (EtOAC): R_f= 0.77 (UV), $[\alpha]_D^{23}$ = 13.3 (*c* 1.0, $CHCl_3$, 79:21 *e.r.*). **^{1}H-NMR** (300 MHz, $CDCl_3$, 298 K) *δ* / ppm = 7.48-7.44 (m, 2 H), 7.42-7.34 (m, 3 H), 5.67 (s, 1 H), 3.66-3.42 (br m, 8 H). **^{13}C-NMR** (75 MHz, $CDCl_3$, 298 K) *δ* / ppm = 165.9, 135.8, 129.0, 128.9, 127.7, 66.6, 66.1, 58.4, 46.5, 43.0. **HRMS** (ESI-TOF): *m/z*: $[M+H]^+$ Calcd for $C_{12}H_{15}ClNO_2^+$ 240.0786; Found 240.0786. **HPLC** (YMC-SB, *n*-hexane/IPA = 4/1, flow rate = 1.0 mL min^{-1}, *I* = 240 nm): t_r = 13.4 min *(major)*, 16.4 min *(minor)*.

[15] Peczkowski, G. R.; Craven, P. G. E.; Stead, D.; Simpkins, N. S. *Chem. Commun.* **2019**, *55*, 4214–4217

5. Copies of NMR Spectra of Chlorination Products:

^{1}H-NMR of **1c** (300 MHz, $CDCl_3$, 298 K):

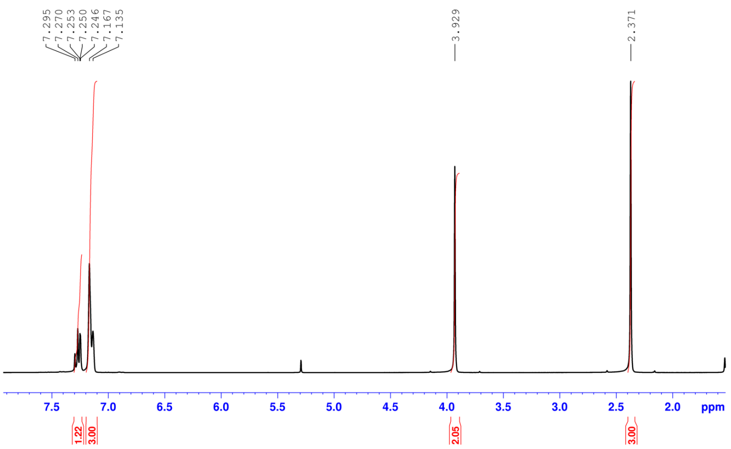

^{13}C-NMR of **1c** (75 MHz, $CDCl_3$, 298 K):

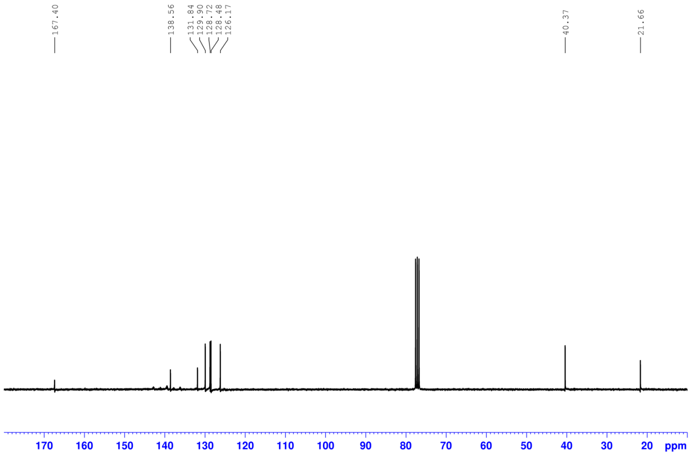

^{19}F-NMR of **1c** (282 MHz, $CDCl_3$, 298 K):

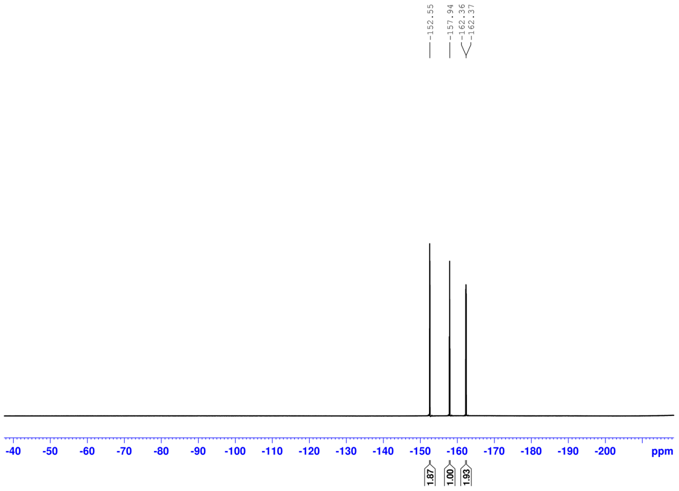

^{1}H-NMR of **3a**$^{\mathbf{OMe}}$ (300 MHz, $CDCl_3$, 298 K):

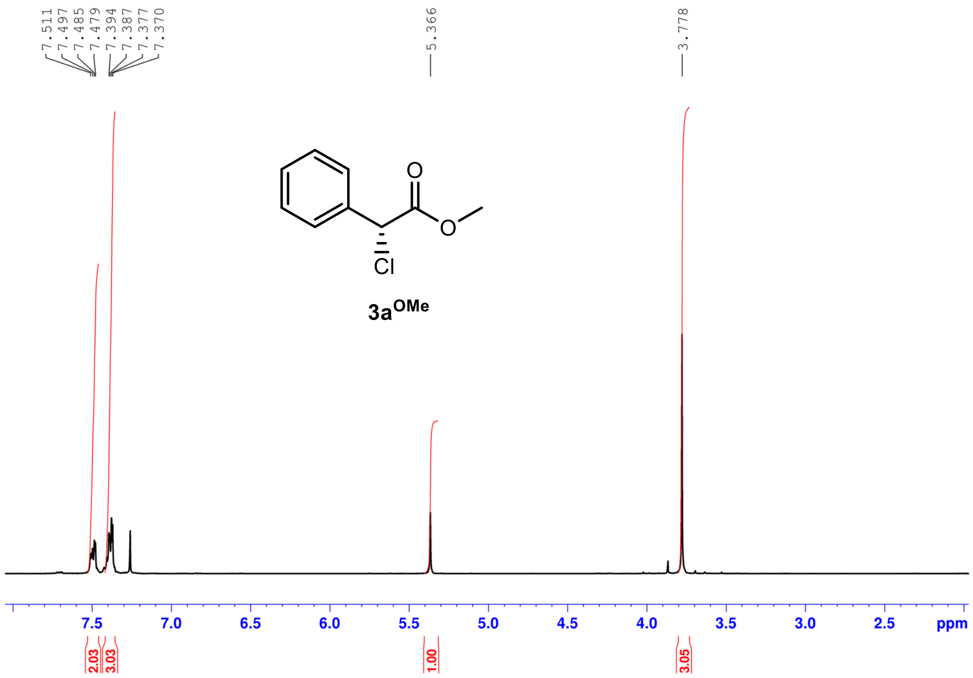

^{13}C-NMR of **3a**$^{\mathbf{OMe}}$ (300 MHz, $CDCl_3$, 298 K):

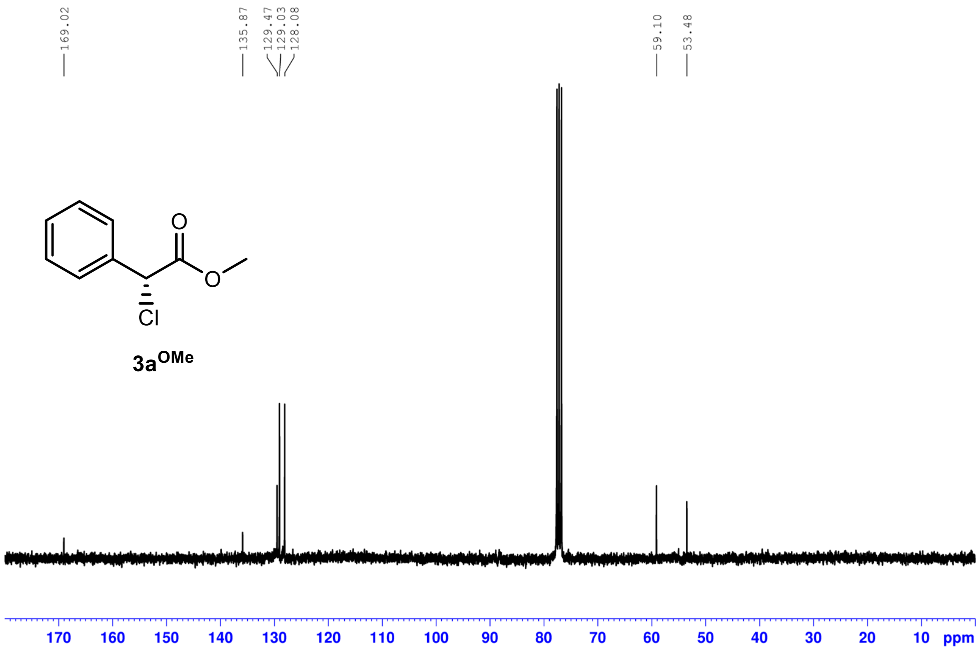

^{1}H-NMR of **3b^{OMe}** (300 MHz, $CDCl_3$, 298 K):

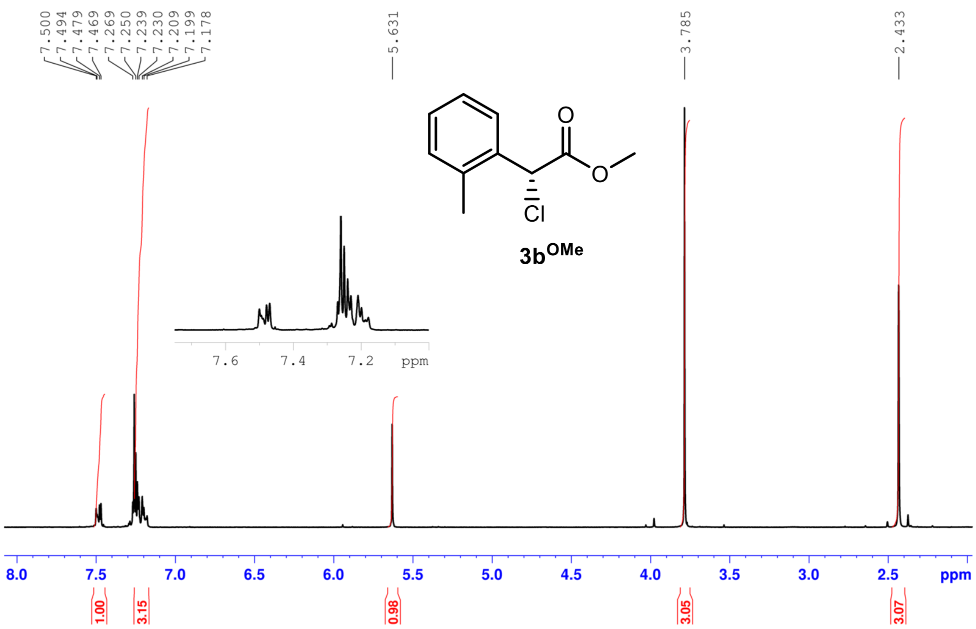

^{13}C-NMR of **3b^{OMe}** (300 MHz, $CDCl_3$, 298 K):

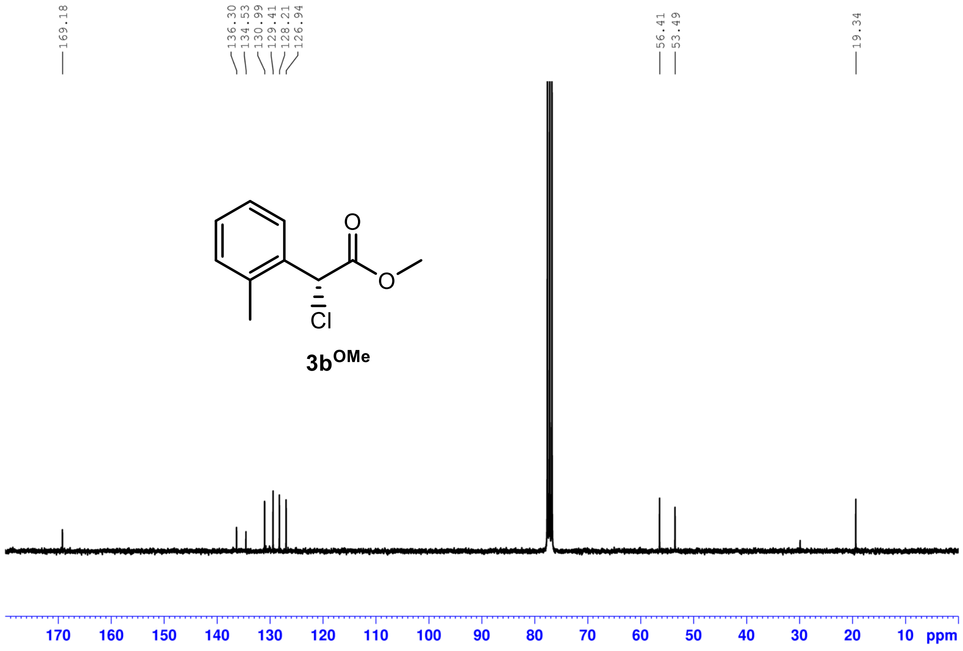

^{1}H-NMR of **3c^{OMe}** (300 MHz, $CDCl_3$, 298 K):

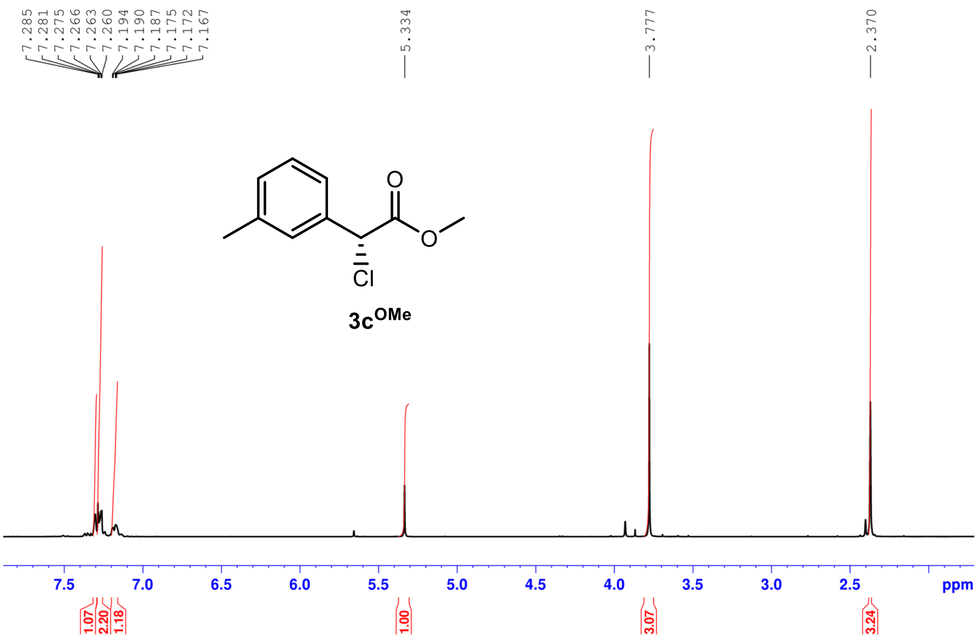

^{13}C-NMR of **3c^{OMe}** (300 MHz, $CDCl_3$, 298 K):

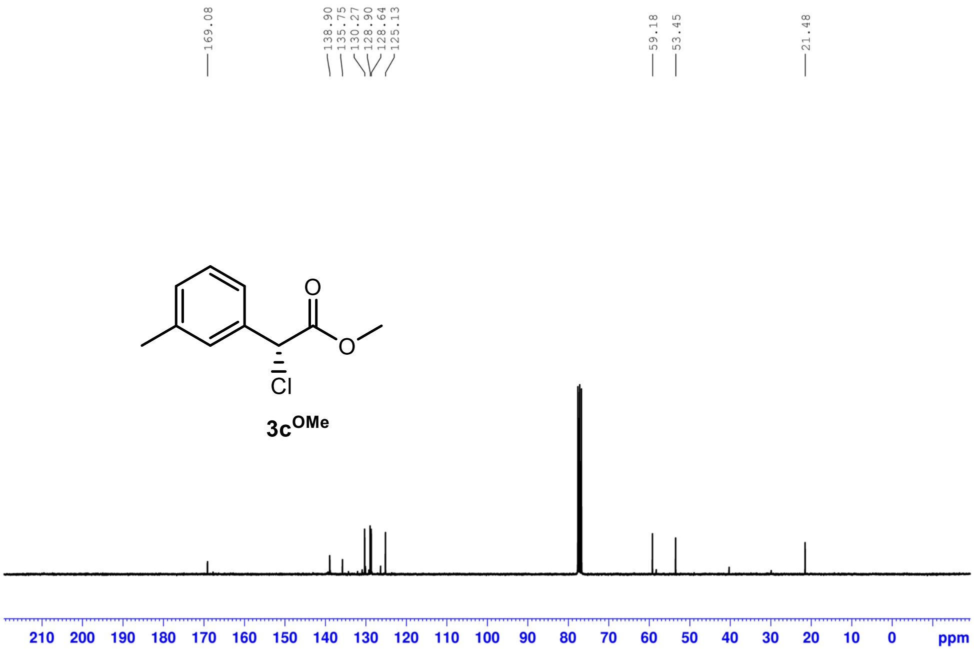

^{1}H-NMR of **3d^{OMe}** (300 MHz, $CDCl_3$, 298 K):

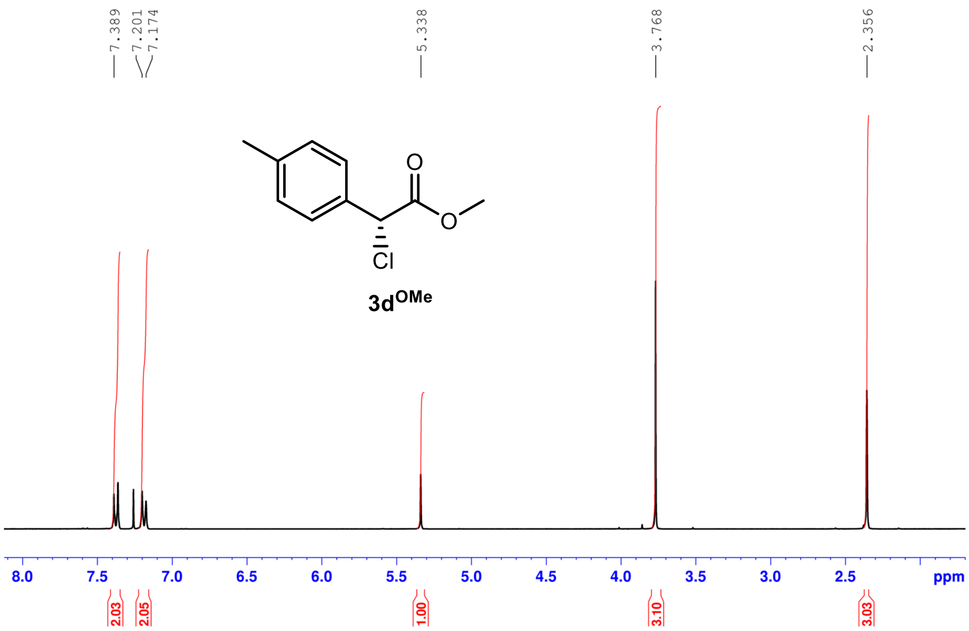

^{13}C-NMR of **3d^{OMe}** (300 MHz, $CDCl_3$, 298 K):

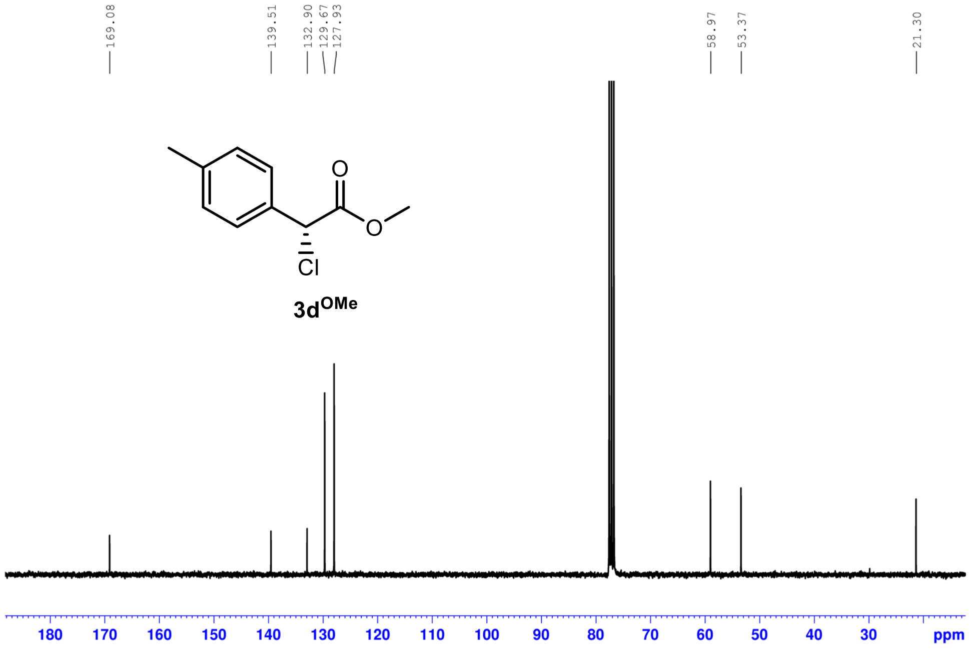

^{1}H-NMR of **3e^{OMe}** (300 MHz, $CDCl_3$, 298 K):

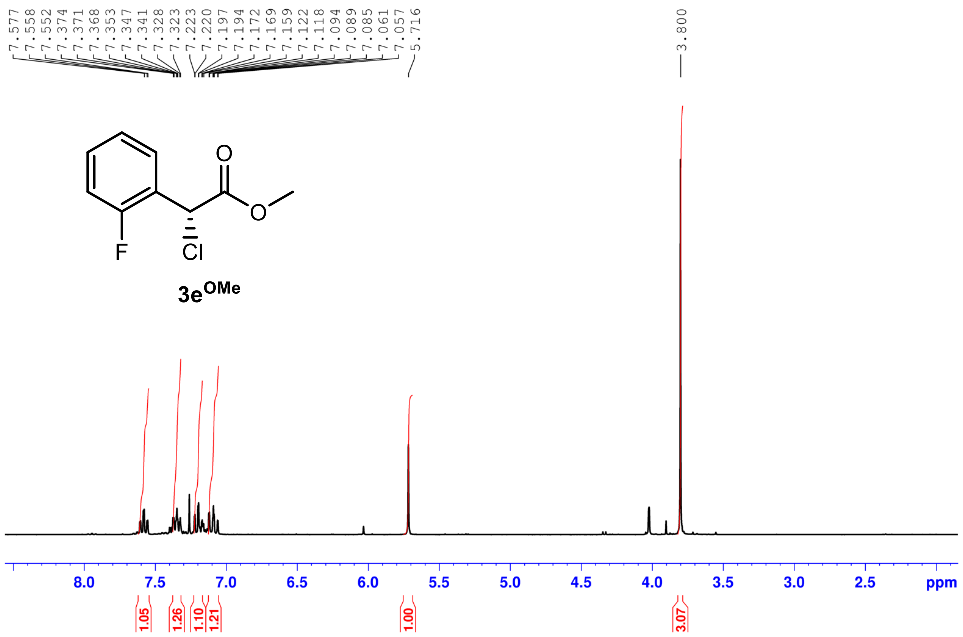

^{13}C-NMR of **3e^{OMe}** (300 MHz, $CDCl_3$, 298 K):

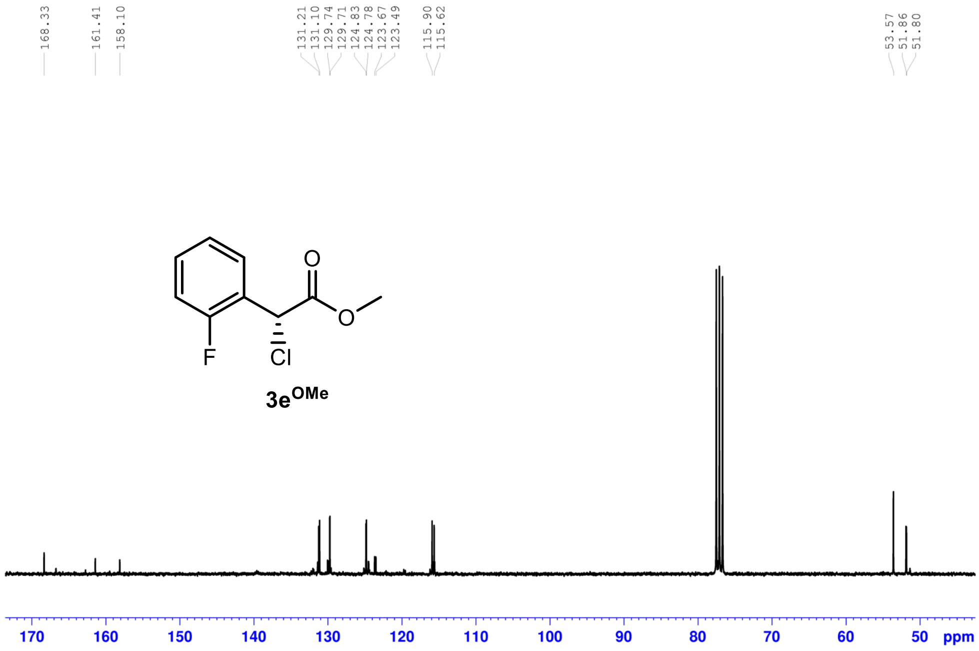

^{1}H-NMR of **3f**[OMe] (300 MHz, $CDCl_3$, 298 K):

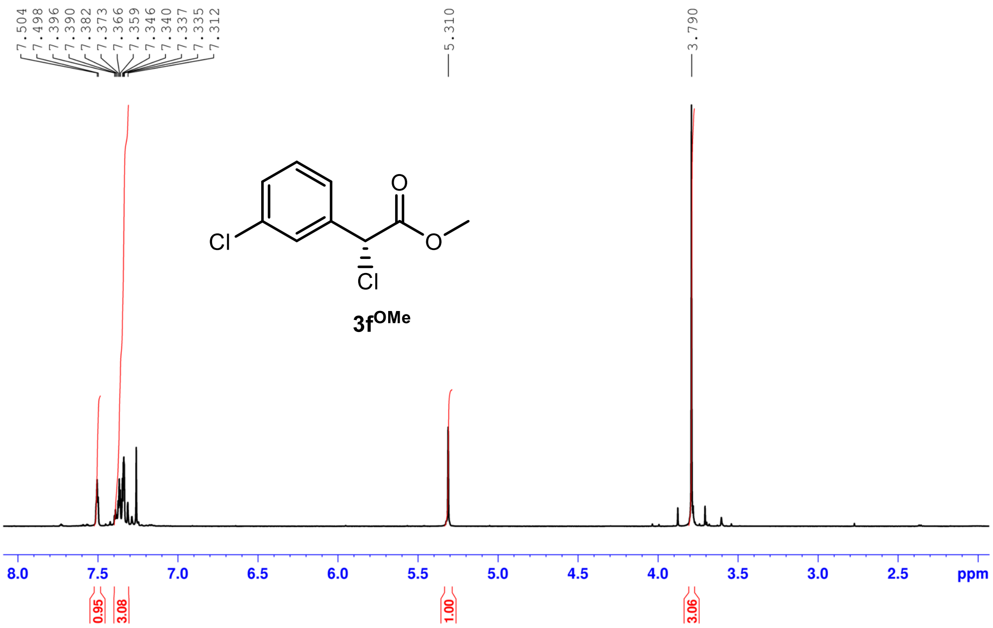

^{13}C-NMR of **3f**[OMe] (300 MHz, $CDCl_3$, 298 K):

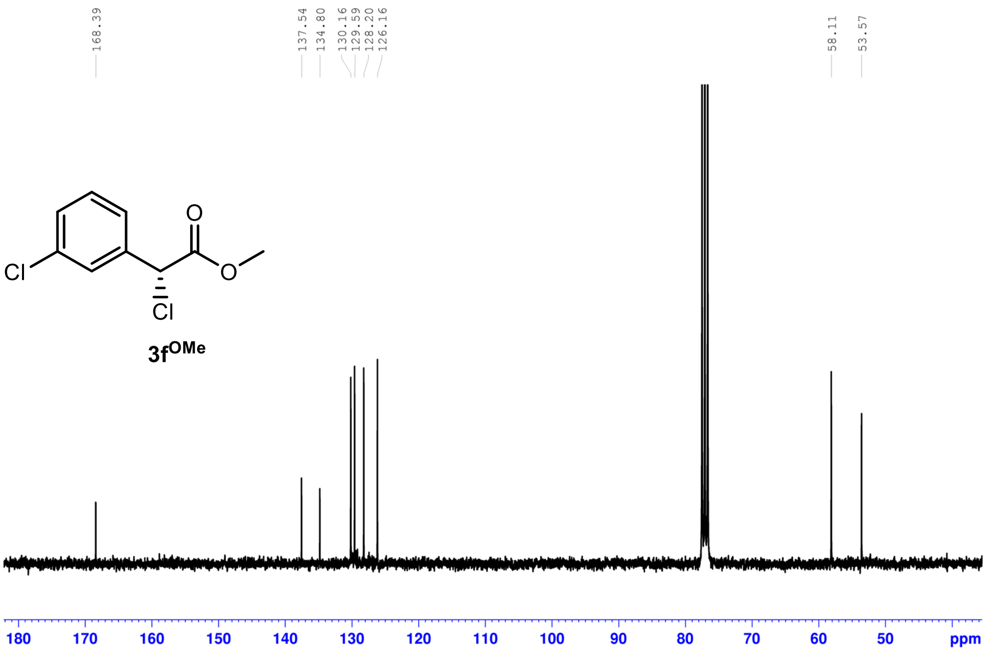

^{1}H-NMR of **3g^{OMe}** (300 MHz, $CDCl_3$, 298 K):

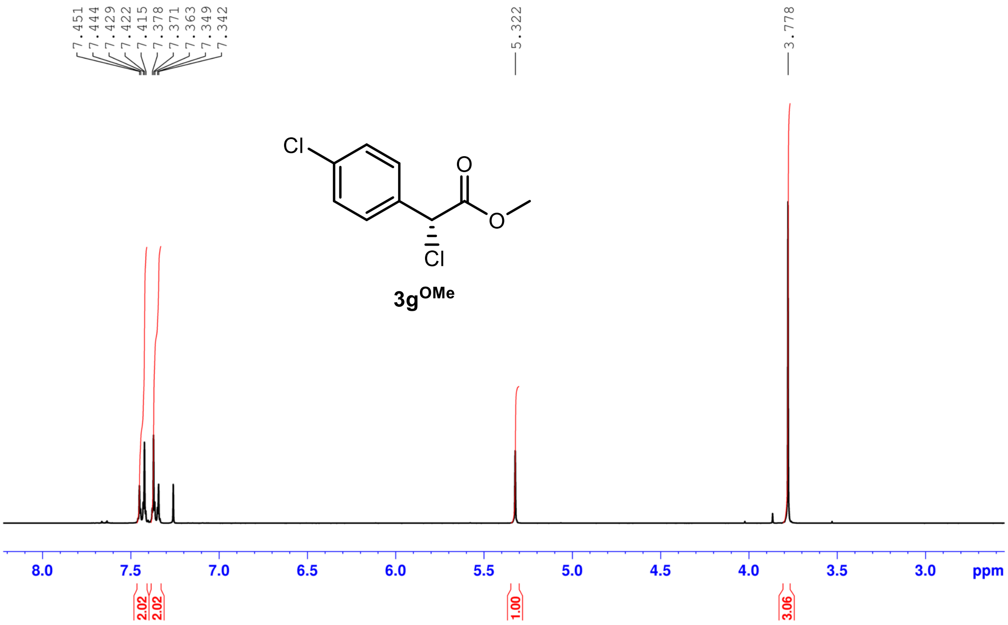

^{13}C-NMR of **3g^{OMe}** (300 MHz, $CDCl_3$, 298 K):

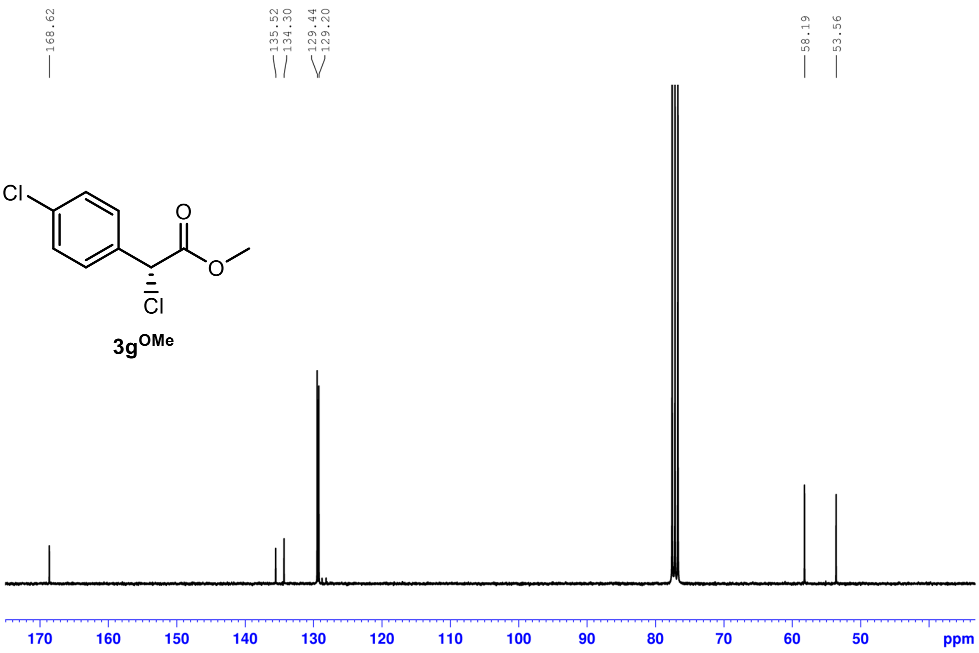

^{1}H-NMR of **3h^{OMe}** (300 MHz, $CDCl_3$, 298 K):

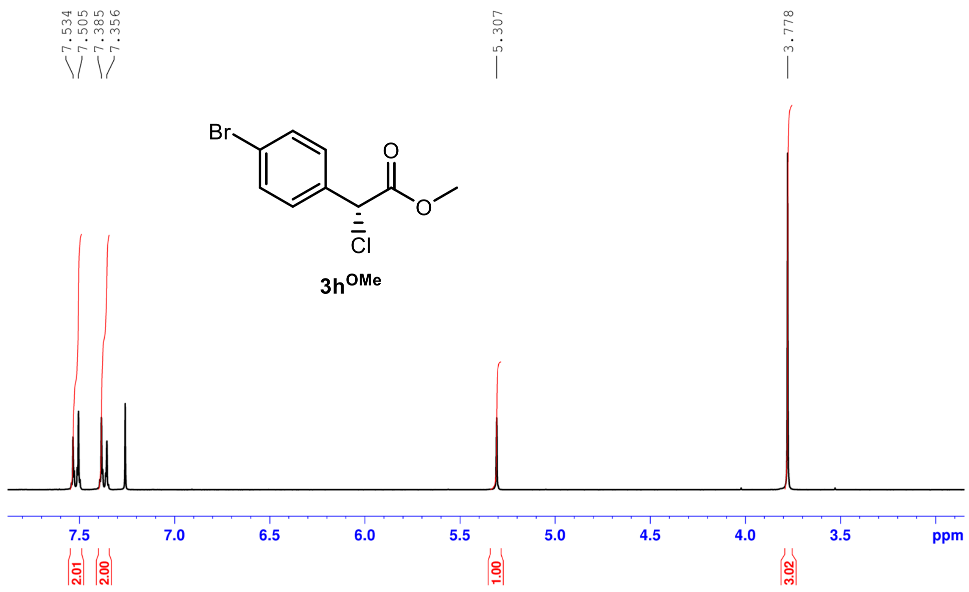

^{13}C-NMR of **3h^{OMe}** (300 MHz, $CDCl_3$, 298 K):

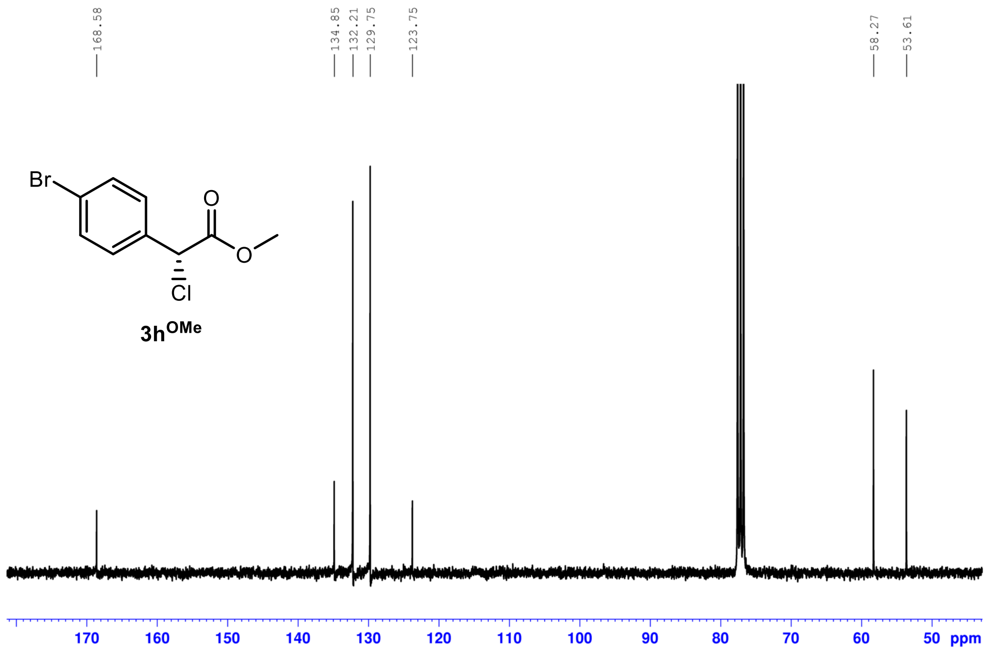

^{1}H-NMR of **3i**^{OMe} (300 MHz, $CDCl_3$, 298 K):

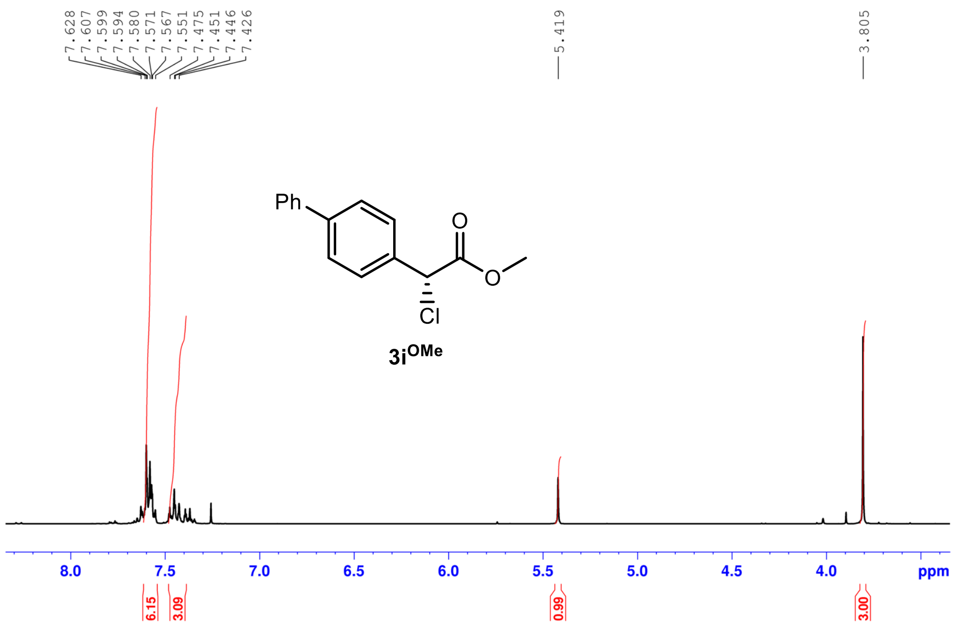

^{13}C-NMR of **3i**^{OMe} (300 MHz, $CDCl_3$, 298 K):

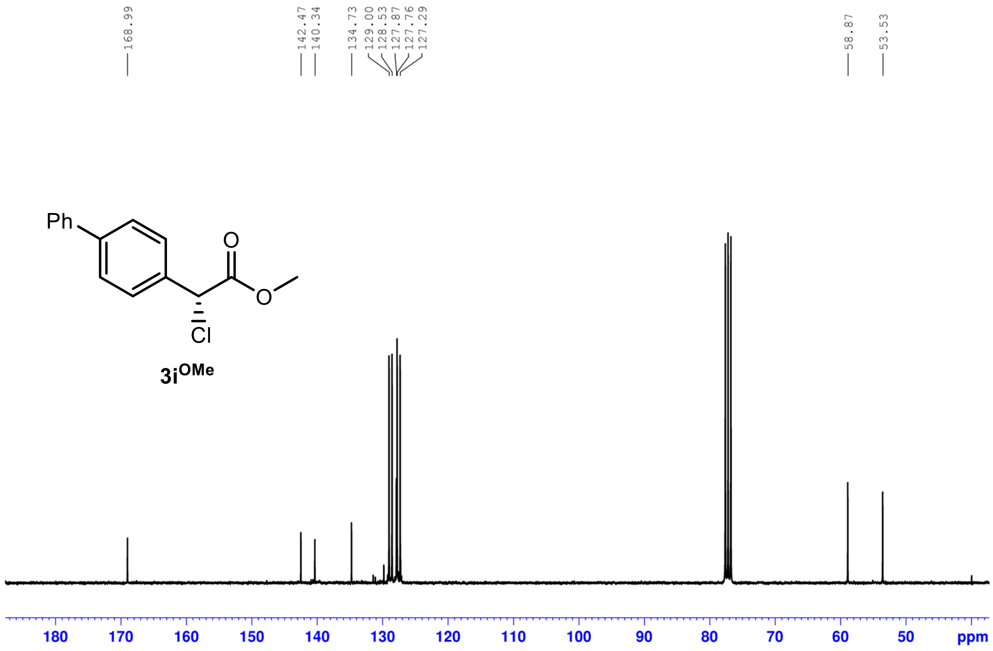

^{1}H-NMR of **3j^{OMe}** (300 MHz, $CDCl_3$, 298 K):

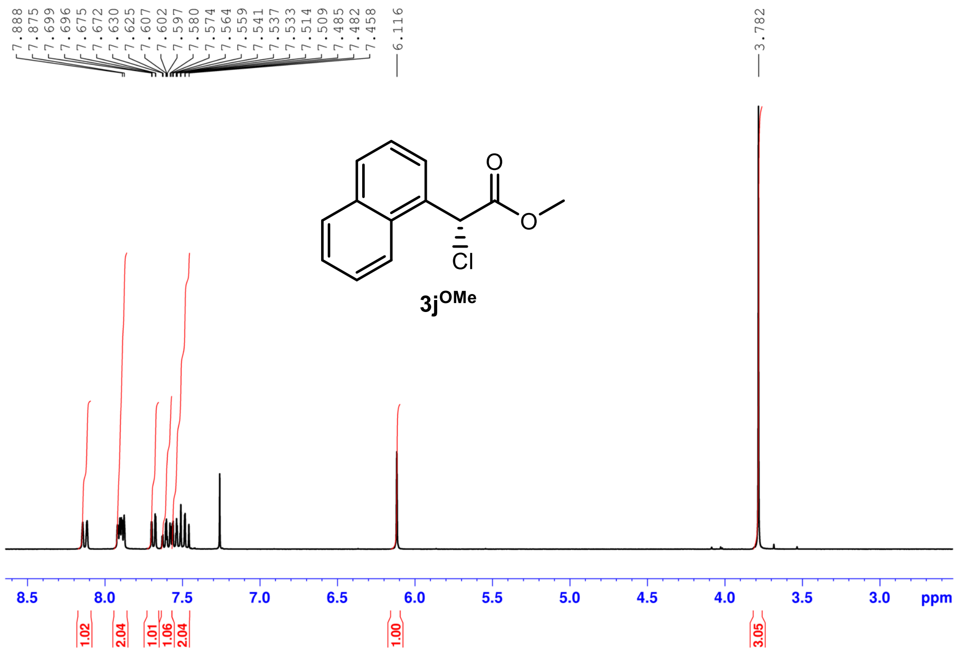

^{13}C-NMR of **3j^{OMe}** (300 MHz, $CDCl_3$, 298 K):

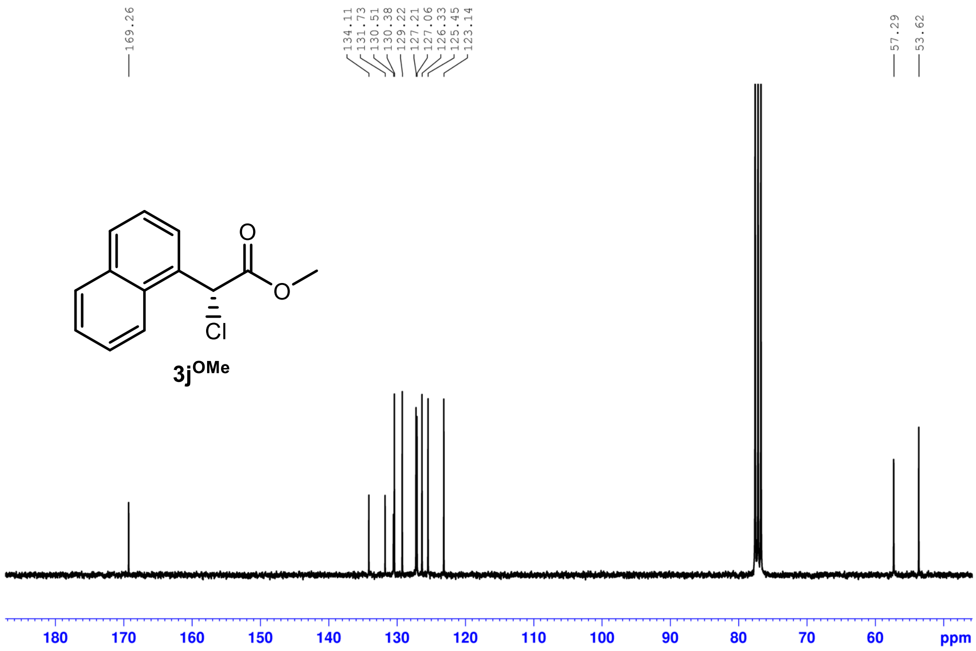

^{1}H-NMR of **3k^{OMe}** (300 MHz, $CDCl_3$, 298 K):

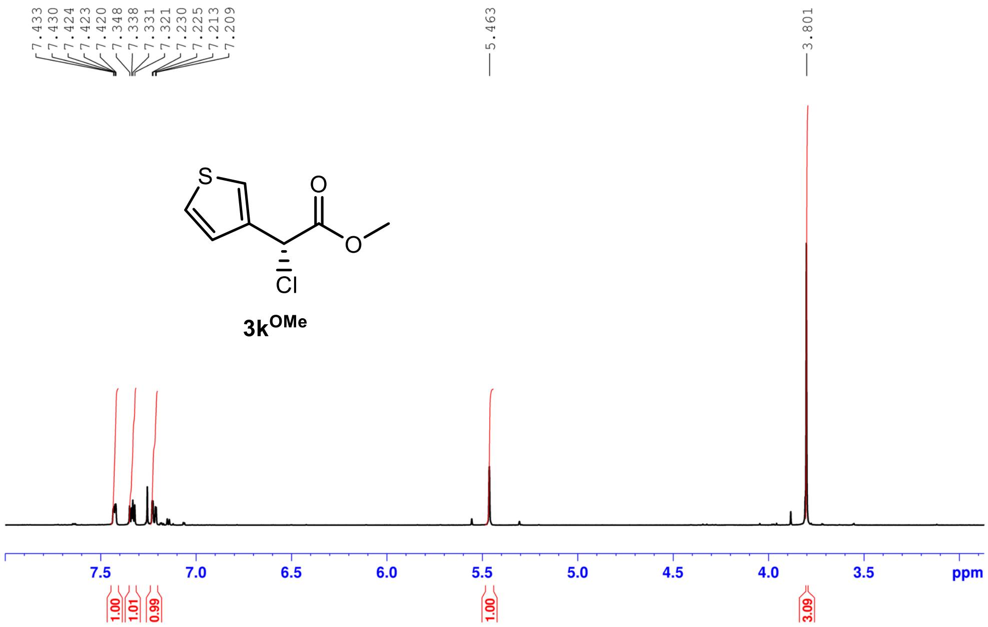

^{13}C-NMR of **3k^{OMe}** (300 MHz, $CDCl_3$, 298 K):

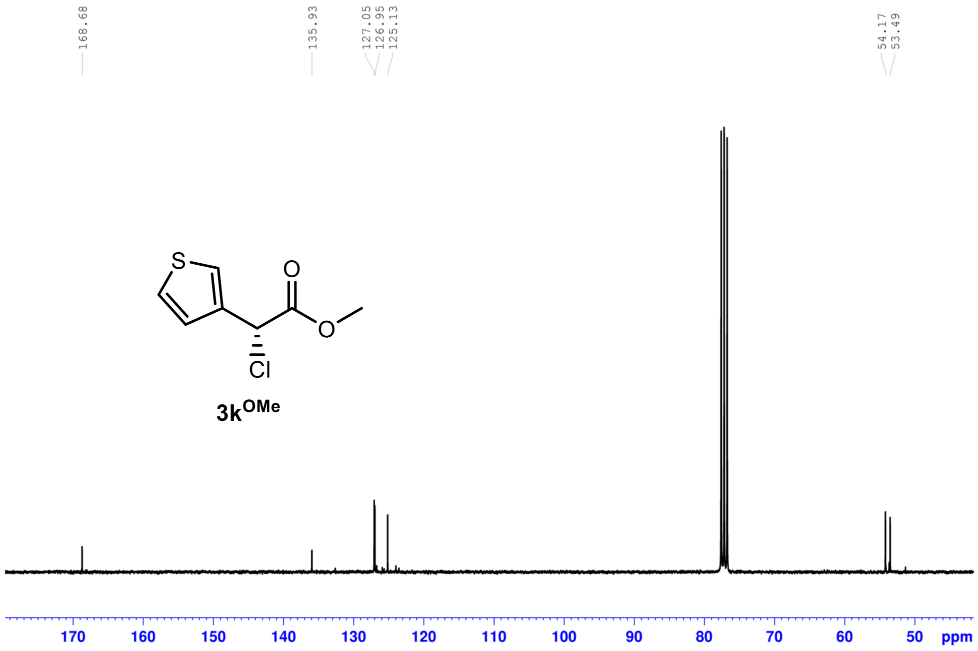

^{1}H-NMR of **3m$^{\mathbf{OMe}}$** (300 MHz, $CDCl_3$, 298 K):

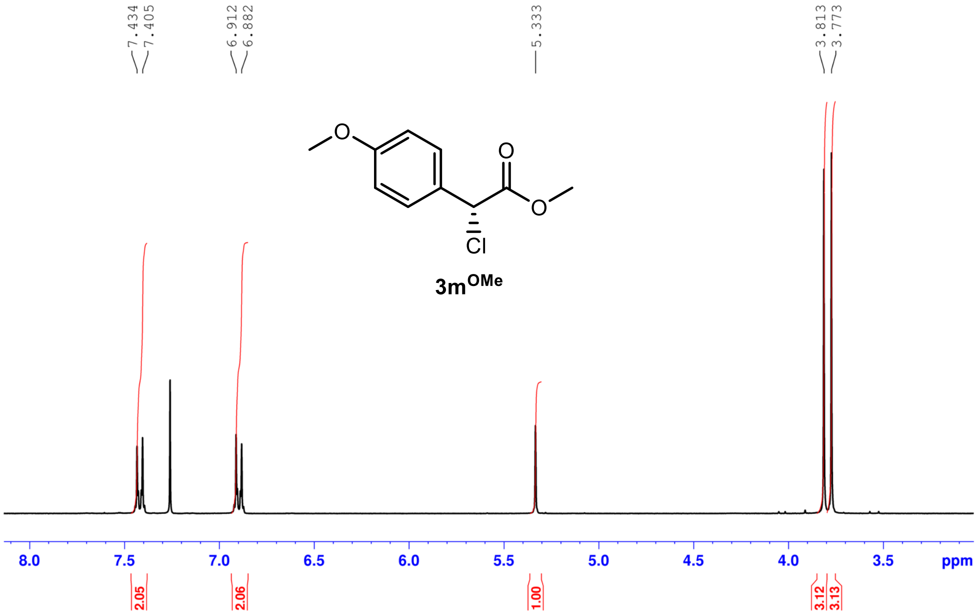

^{13}C-NMR of **3m$^{\mathbf{OMe}}$** (300 MHz, $CDCl_3$, 298 K):

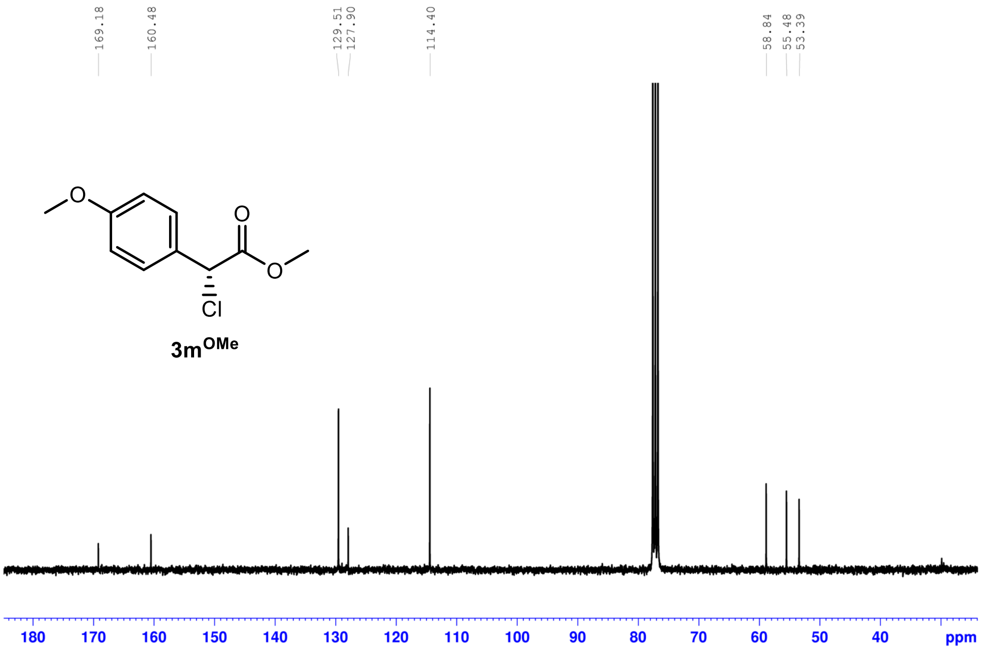

^{1}H-NMR of **3a**^{pfp} (300 MHz, $CDCl_3$, 298 K):

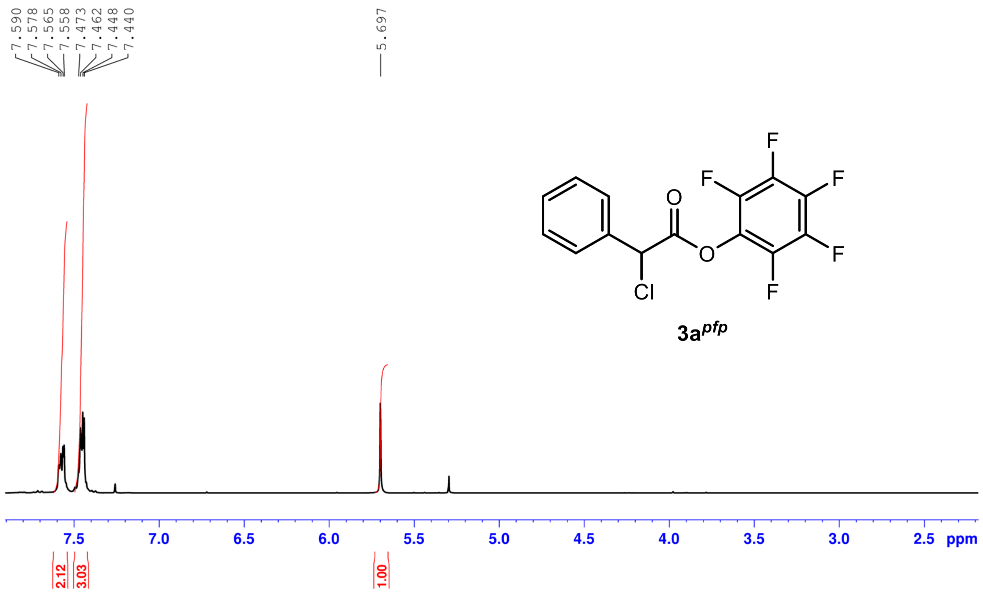

^{13}C-NMR of **3a**^{pfp} (300 MHz, $CDCl_3$, 298 K):

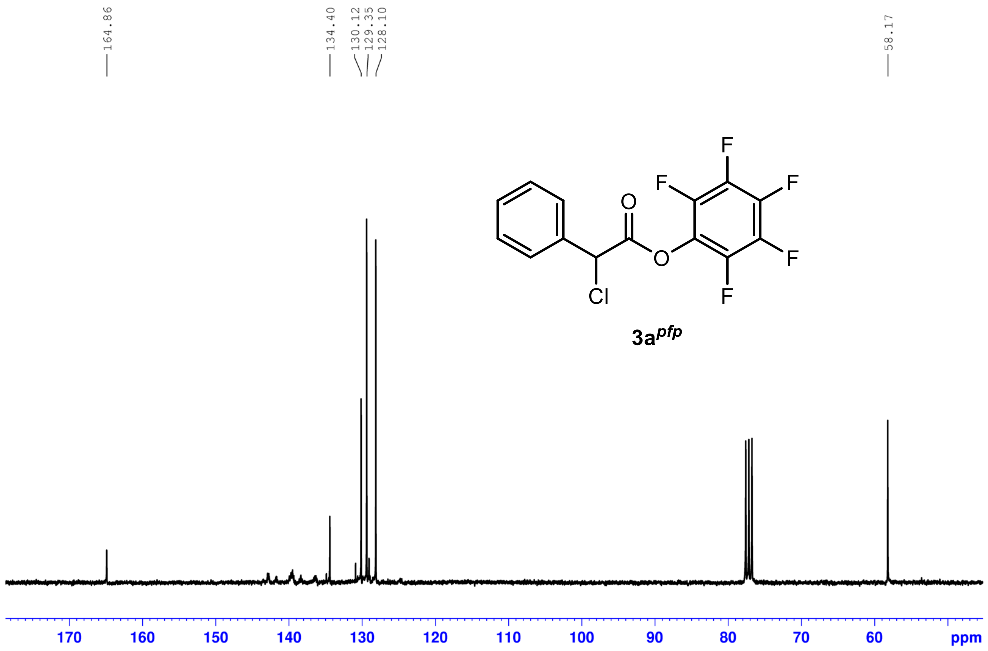

^{1}H-NMR of **3a^{OEt}** (300 MHz, $CDCl_3$, 298 K):

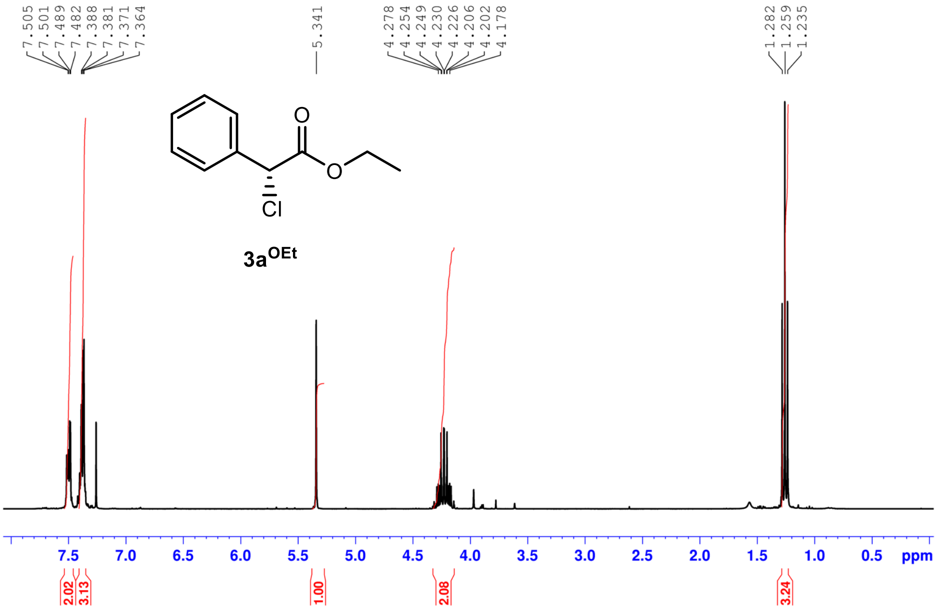

^{13}C-NMR of **3a^{OEt}** (300 MHz, $CDCl_3$, 298 K):

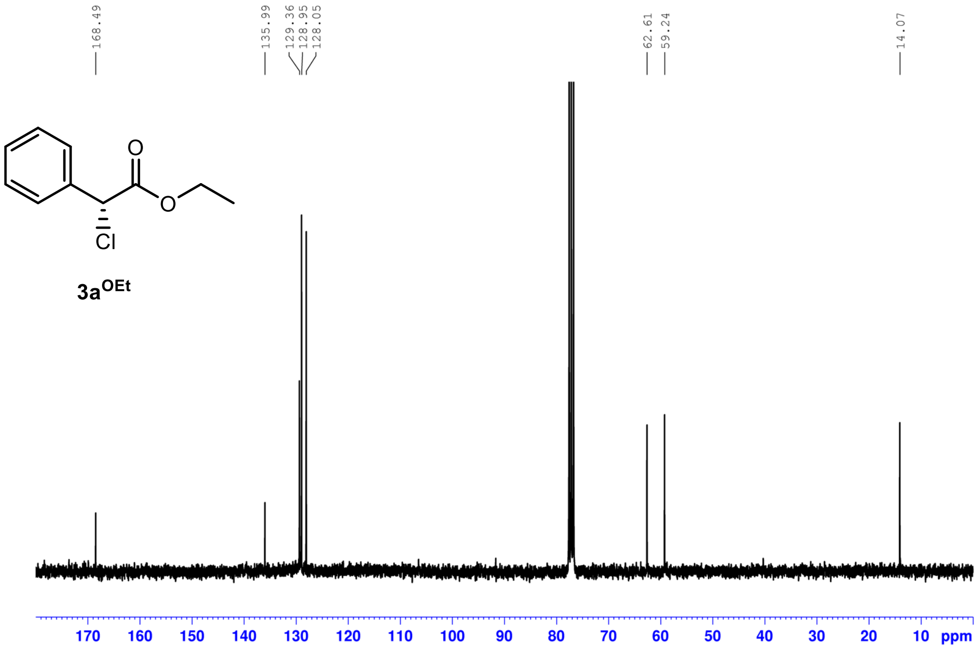

^{1}H-NMR of **3a^{OiPr}** (300 MHz, $CDCl_3$, 298 K):

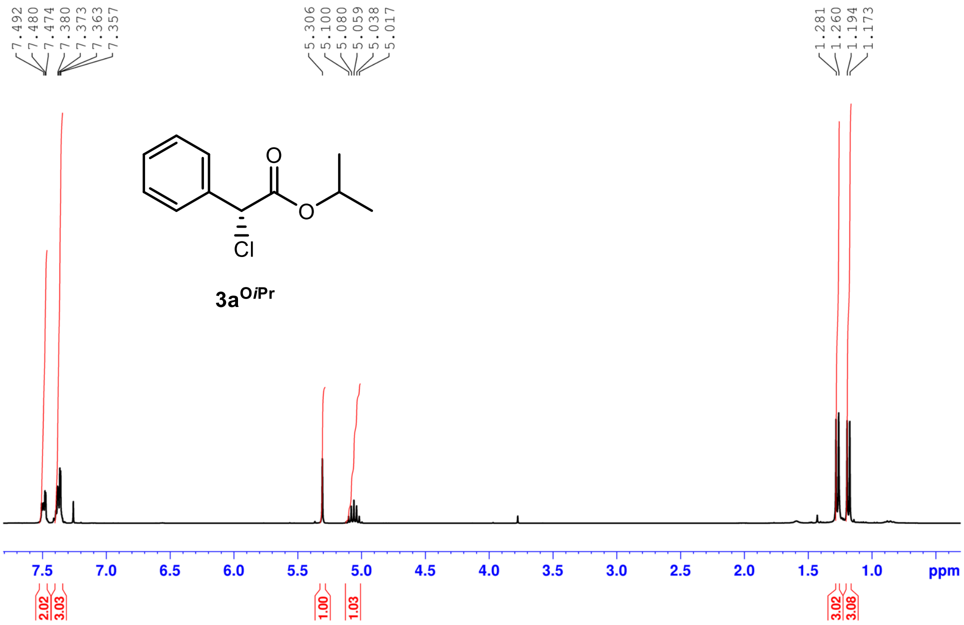

^{13}C-NMR of **3a^{OiPr}** (300 MHz, $CDCl_3$, 298 K):

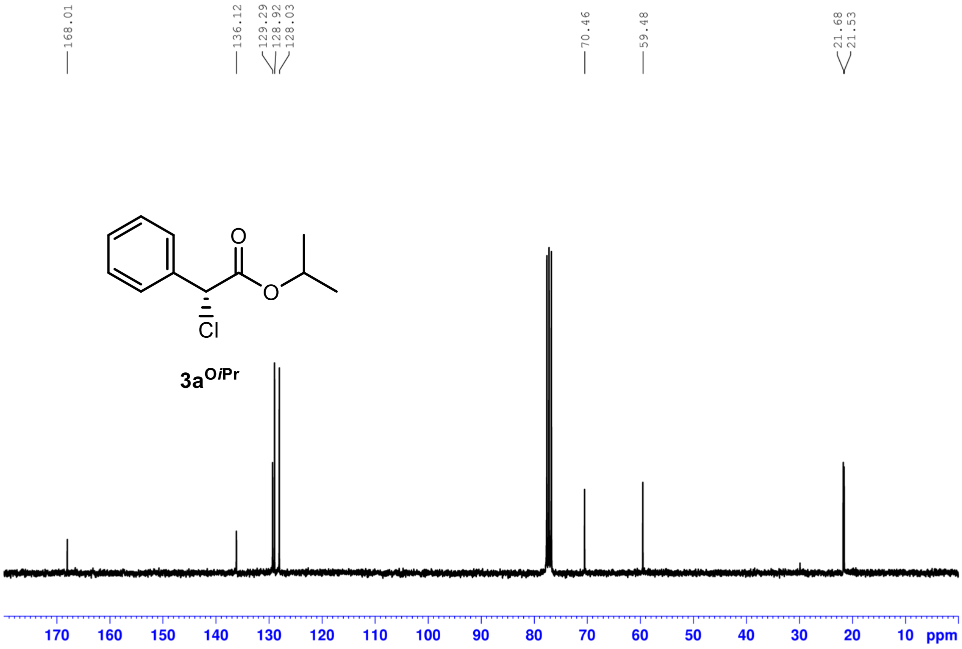

^{1}H-NMR of **3a**$^{\mathbf{Morpholine}}$ (300 MHz, $CDCl_3$, 298 K):

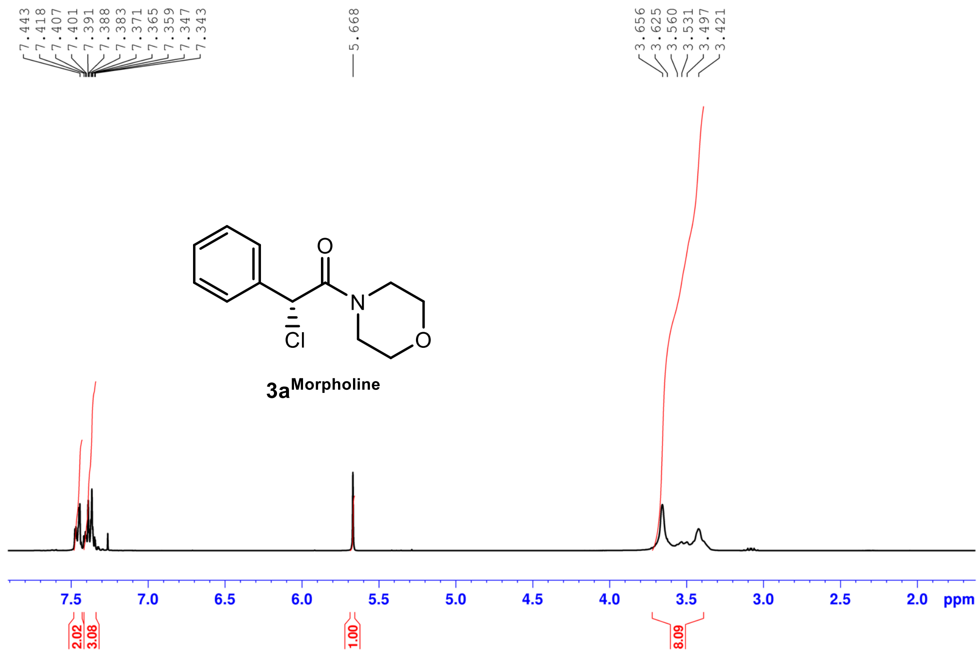

^{13}C-NMR of **3a**$^{\mathbf{Morpholine}}$ (300 MHz, $CDCl_3$, 298 K):

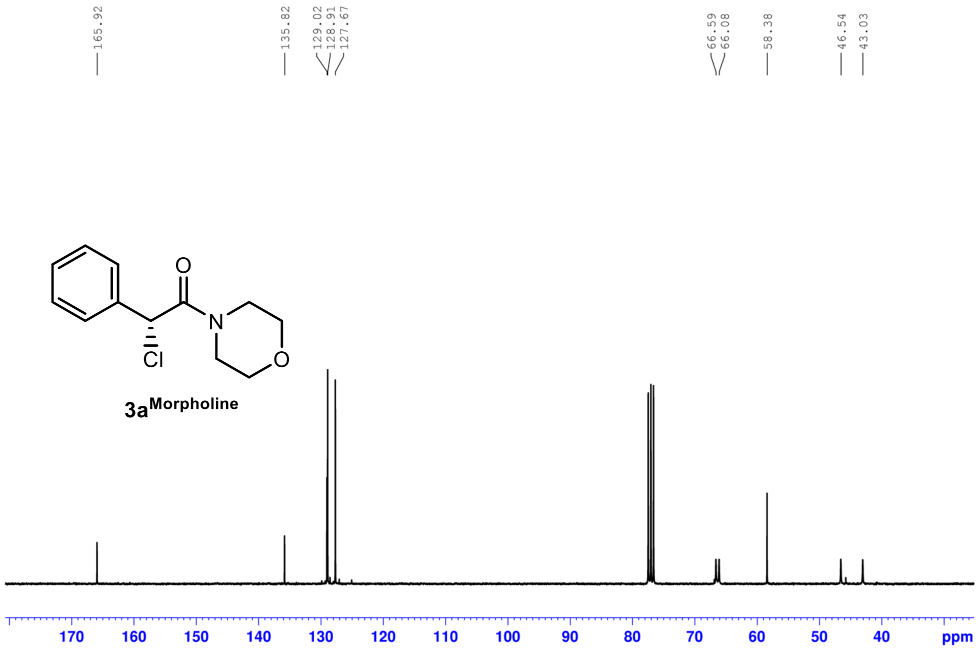

^{1}H-NMR of **3a**$^{\mathbf{NHBn}}$ (300 MHz, $CDCl_3$, 298 K):

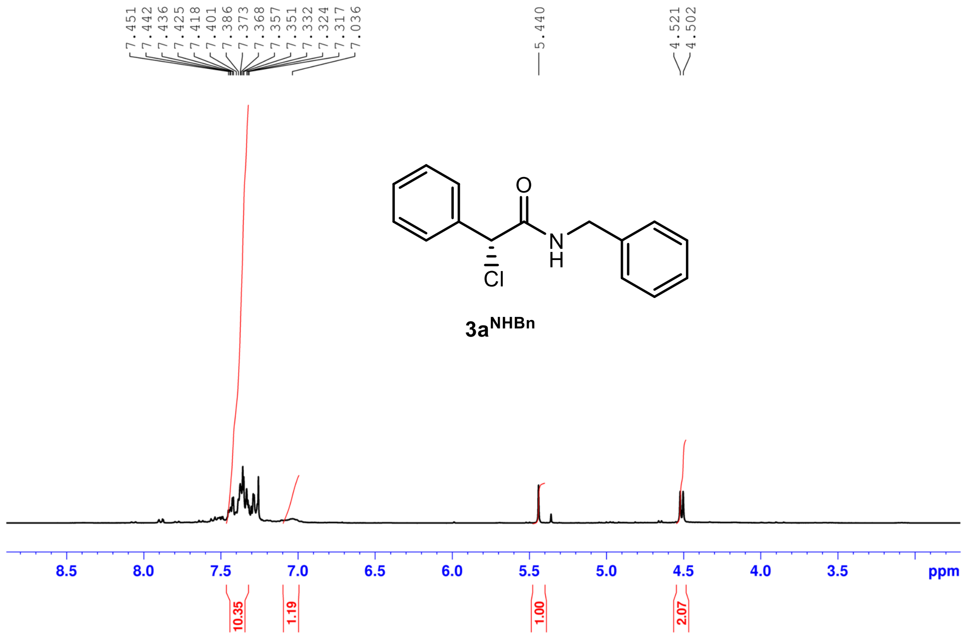

^{13}C-NMR of **3a**$^{\mathbf{NHBn}}$ (300 MHz, $CDCl_3$, 298 K):

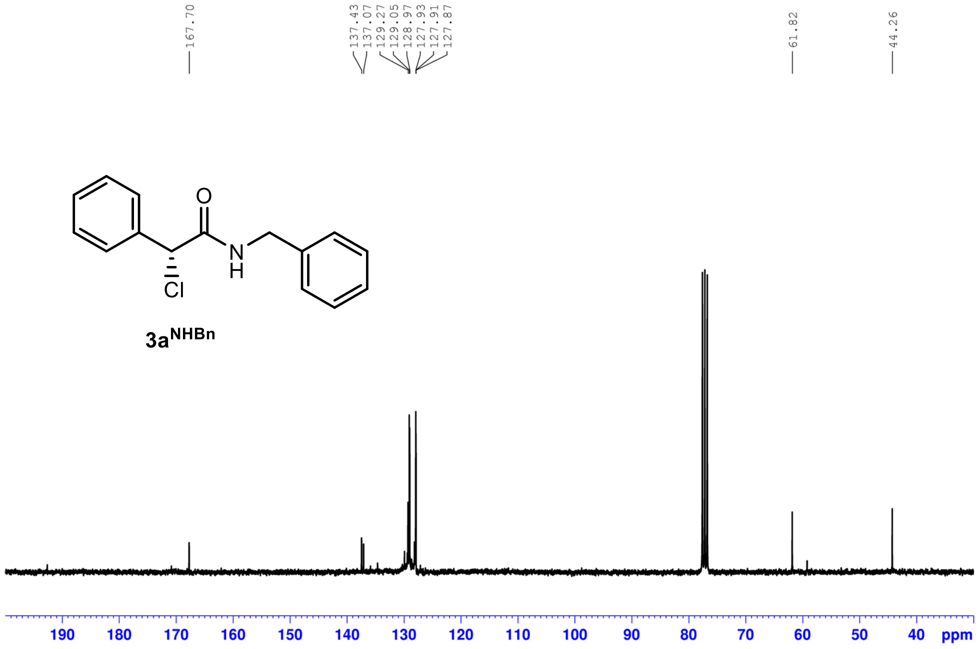

6. Copies of HPLC Chromatograms:

HPLC chromatogram of (rac)-**3a**$^{\mathbf{OMe}}$

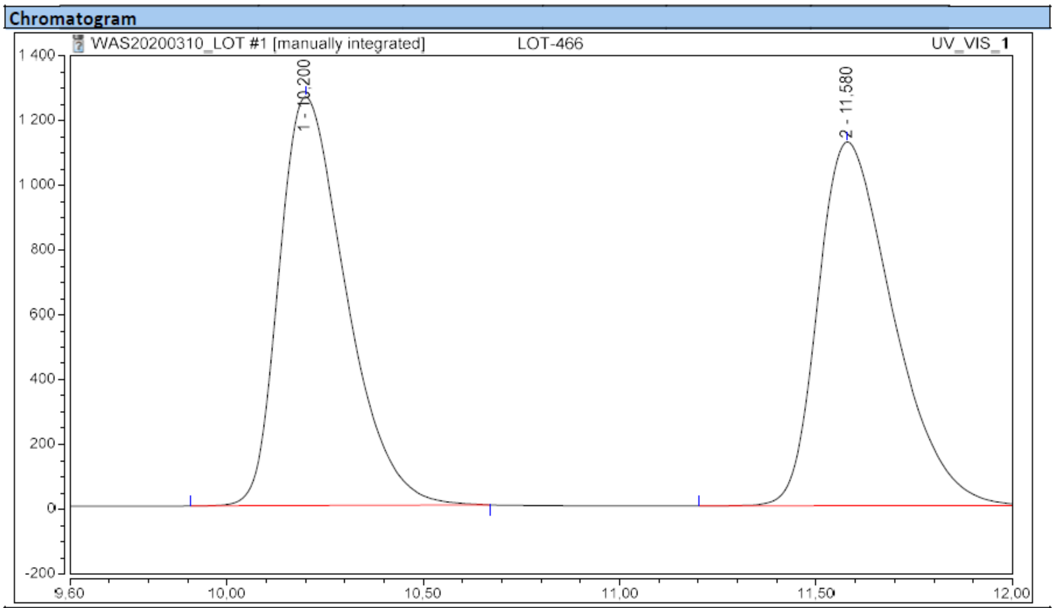

Integration Results

No.	Peak Name	Retention Time min	Area mAU*min	Height mAU	Relative Area %	Relative Height %	Amount n.a.
1		10,200	248,855	1262,767	49,94	52,92	n.a.
2		11,580	249,488	1123,428	50,06	47,08	n.a.
**Total:**			**498,342**	**2386,195**	**100,00**	**100,00**	

HPLC chromatogram of enantioenriched **3a**$^{\mathbf{OMe}}$

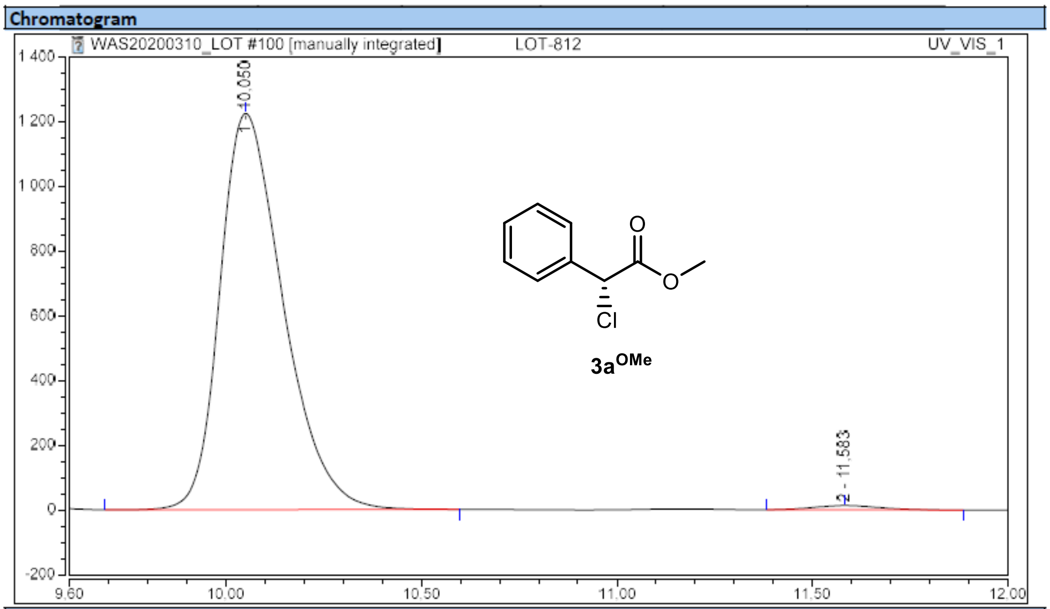

Integration Results

No.	Peak Name	Retention Time min	Area mAU*min	Height mAU	Relative Area %	Relative Height %	Amount n.a.
1		10,050	229,548	1224,391	98,91	98,94	n.a.
2		11,583	2,541	13,175	1,09	1,06	n.a.
**Total:**			**232,088**	**1237,566**	**100,00**	**100,00**	

HPLC chromatogram of (rac)-**3b$^{\mathbf{OMe}}$**

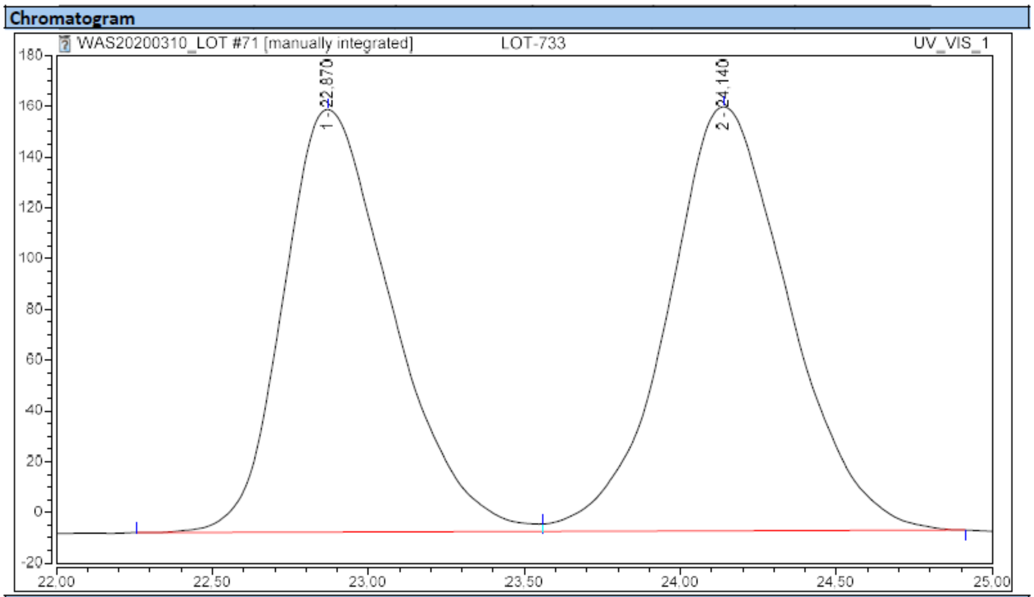

Integration Results

No.	Peak Name	Retention Time min	Area mAU*min	Height mAU	Relative Area %	Relative Height %	Amount n.a.
1		22,870	67,272	166,438	47,11	49,92	n.a.
2		24,140	75,515	166,980	52,89	50,08	n.a.
**Total:**			**142,787**	**333,418**	**100,00**	**100,00**	

HPLC chromatogram of enantioenriched **3b$^{\mathbf{OMe}}$**

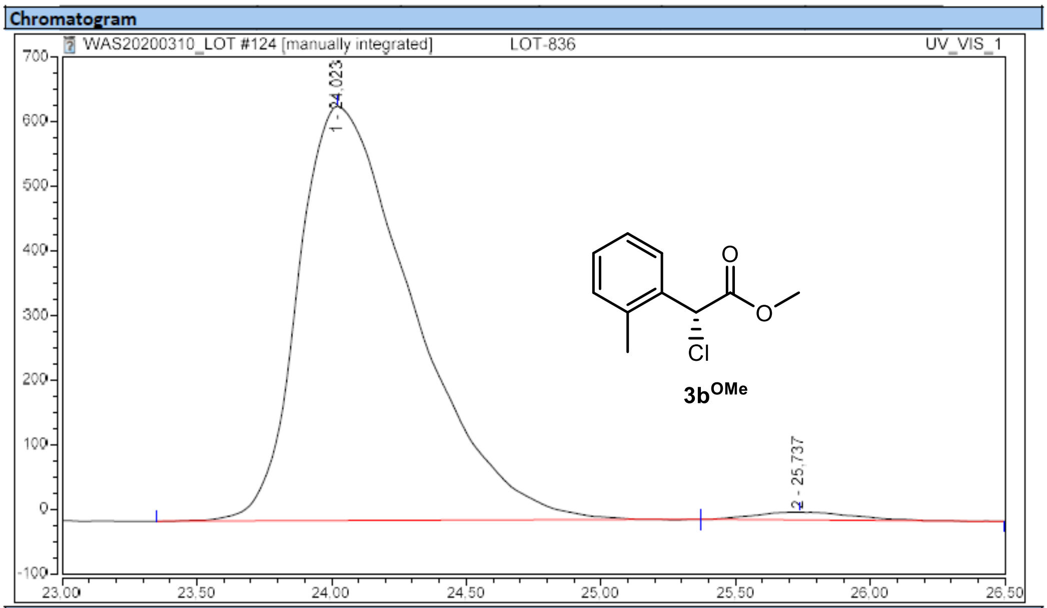

Integration Results

No.	Peak Name	Retention Time min	Area mAU*min	Height mAU	Relative Area %	Relative Height %	Amount n.a.
1		24,023	317,456	640,810	98,40	98,20	n.a.
2		25,737	5,171	11,772	1,60	1,80	n.a.
**Total:**			**322,626**	**652,582**	**100,00**	**100,00**	

HPLC chromatogram of (rac)-**3c**$^{\mathbf{OMe}}$

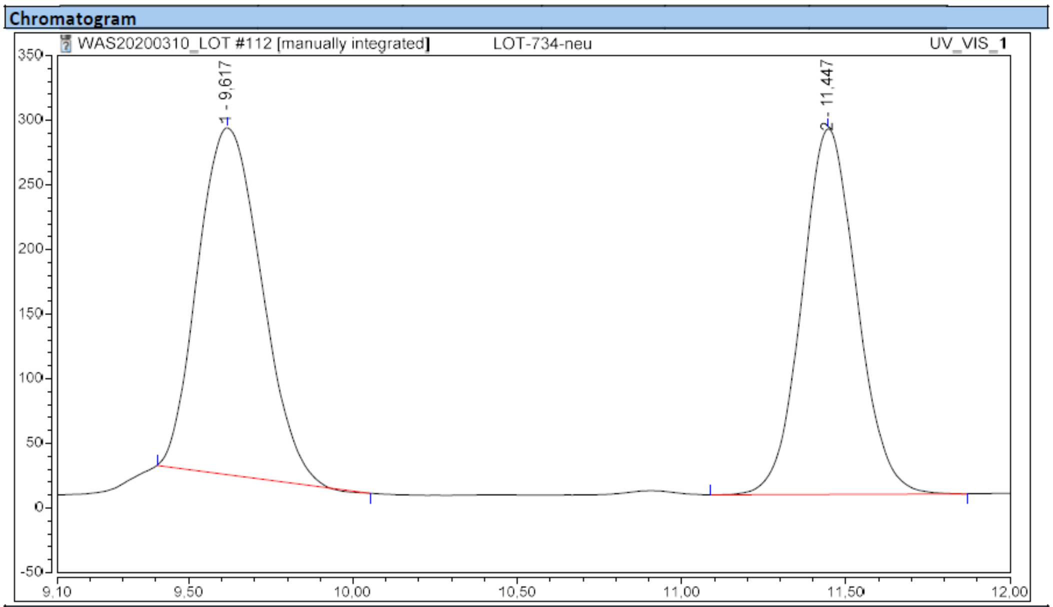

Integration Results

No.	Peak Name	Retention Time min	Area mAU*min	Height mAU	Relative Area %	Relative Height %	Amount n.a.
1		9,617	60,275	268,384	52,32	48,68	n.a.
2		11,447	54,920	282,896	47,68	51,32	n.a.
**Total:**			**115,195**	**551,280**	**100,00**	**100,00**	

HPLC chromatogram of enantioenriched **3c**$^{\mathbf{OMe}}$

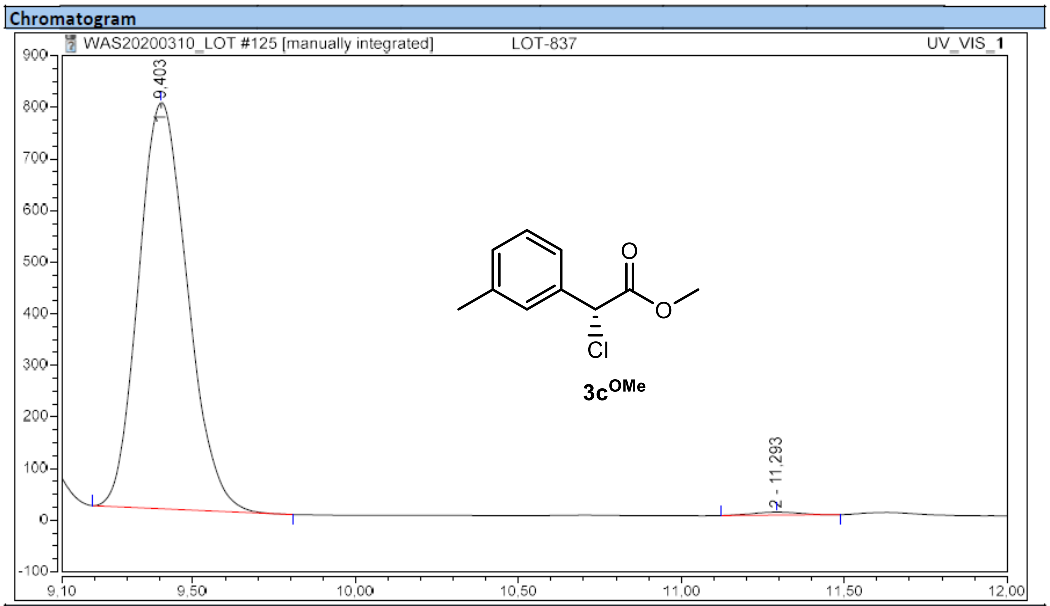

Integration Results

No.	Peak Name	Retention Time min	Area mAU*min	Height mAU	Relative Area %	Relative Height %	Amount n.a.
1		9,403	137,269	787,331	99,34	99,29	n.a.
2		11,293	0,911	5,605	0,66	0,71	n.a.
**Total:**			**138,180**	**792,936**	**100,00**	**100,00**	

HPLC chromatogram of (rac)-**3d** $^{\mathbf{OMe}}$

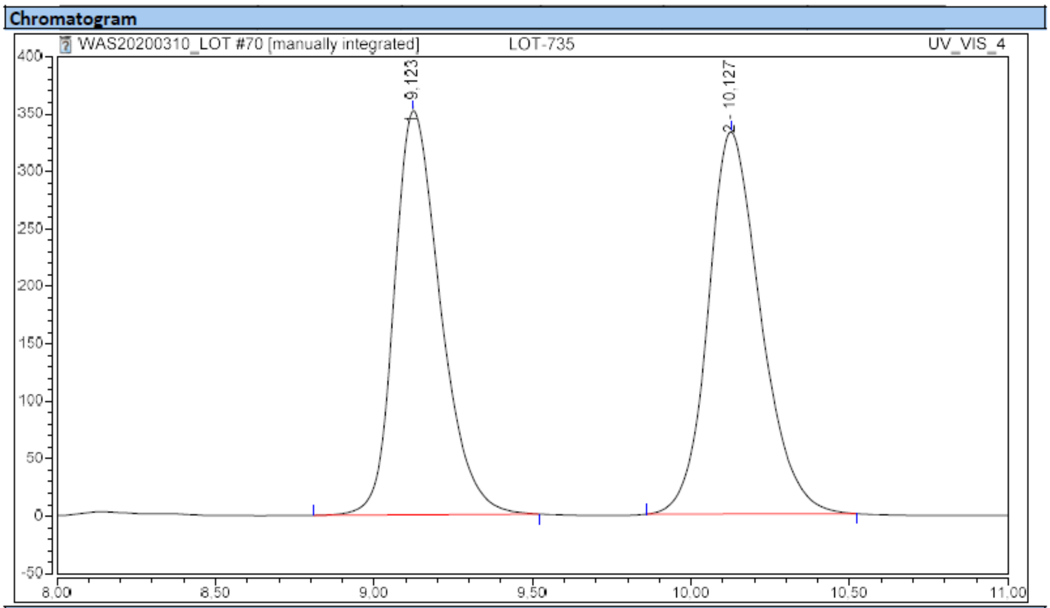

Integration Results							
No.	Peak Name	Retention Time min	Area mAU*min	Height mAU	Relative Area %	Relative Height %	Amount n.a.
1		9,123	59,475	351,742	48,19	51,41	n.a.
2		10,127	63,940	332,481	51,81	48,59	n.a.
Total:			**123,415**	**684,224**	**100,00**	**100,00**	

HPLC chromatogram of enantioenriched **3d**$^{\mathbf{OMe}}$

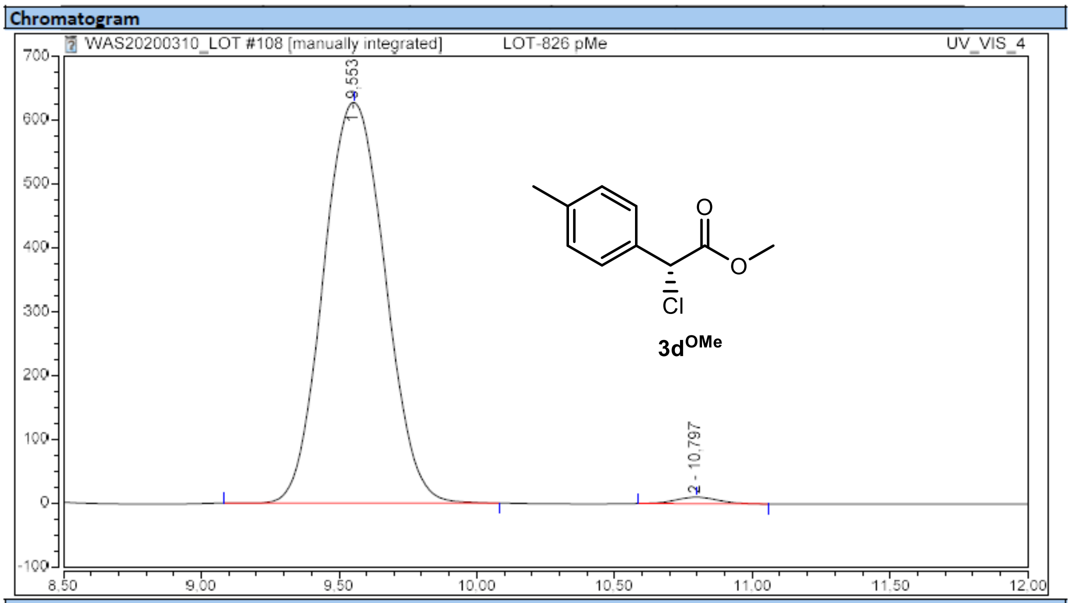

Integration Results							
No.	Peak Name	Retention Time min	Area mAU*min	Height mAU	Relative Area %	Relative Height %	Amount n.a.
1		9,553	162,561	627,120	98,90	98,38	n.a.
2		10,797	1,811	10,322	1,10	1,62	n.a.
Total:			**164,373**	**637,442**	**100,00**	**100,00**	

HPLC chromatogram of (rac)-**3e** $^{\mathbf{OMe}}$

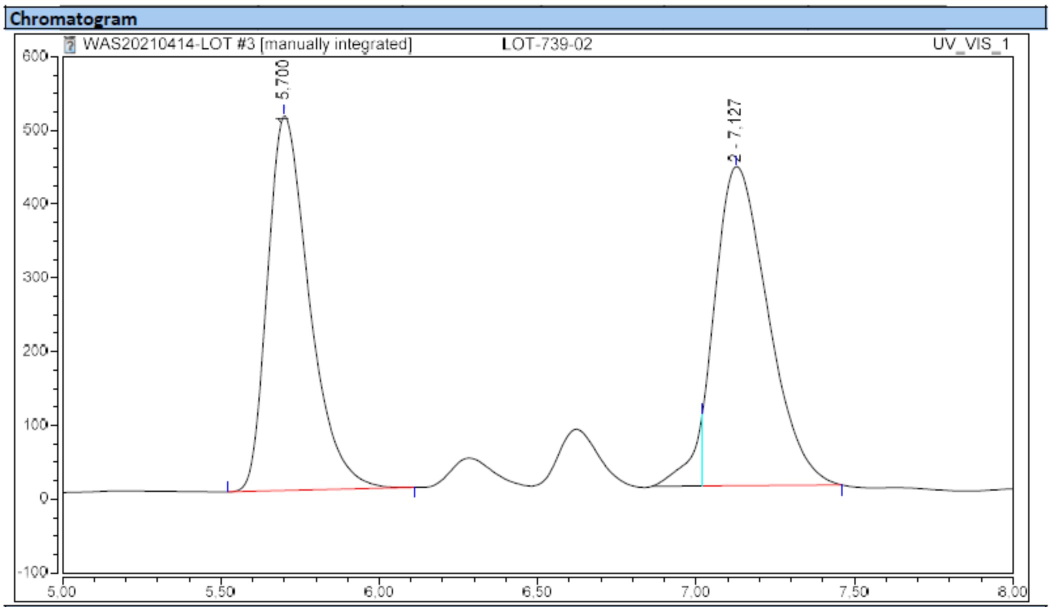

Integration Results

No.	Peak Name	Retention Time min	Area mAU*min	Height mAU	Relative Area %	Relative Height %	Amount n.a.
1		5,700	77,575	508,115	48,43	54,01	n.a.
2		7,127	82,606	432,715	51,57	45,99	n.a.
**Total:**			**160,181**	**940,830**	**100,00**	**100,00**	

HPLC chromatogram of enantioenriched **3e**$^{\mathbf{OMe}}$

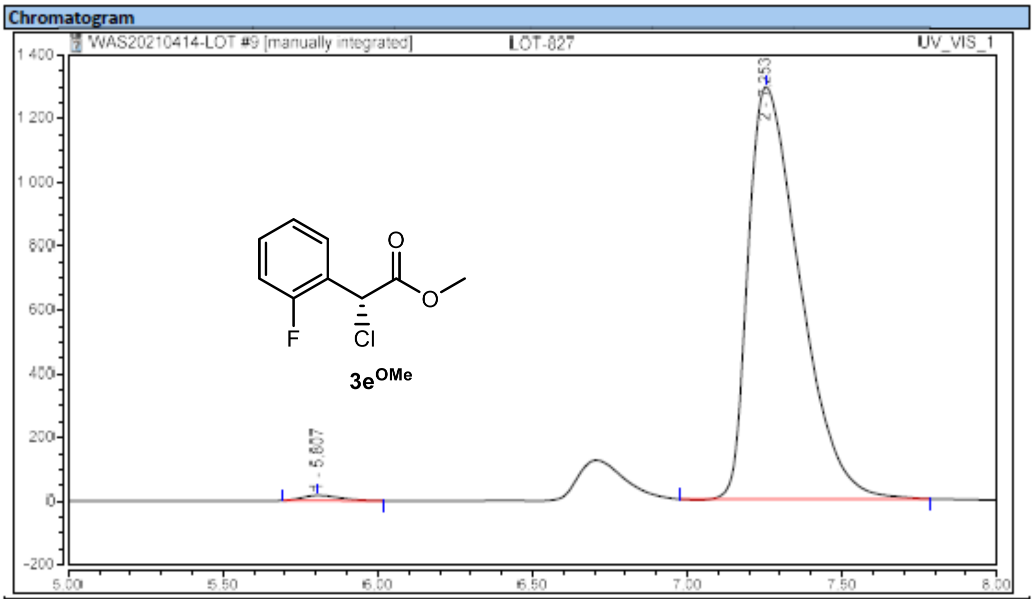

Integration Results

No.	Peak Name	Retention Time min	Area mAU*min	Height mAU	Relative Area %	Relative Height %	Amount n.a.
1		5,807	2,275	16,258	0,90	1,24	n.a.
2		7,253	250,379	1293,506	99,10	98,76	n.a.
**Total:**			**252,654**	**1309,764**	**100,00**	**100,00**	

HPLC chromatogram of (rac)-**3f** $^{\mathbf{OMe}}$

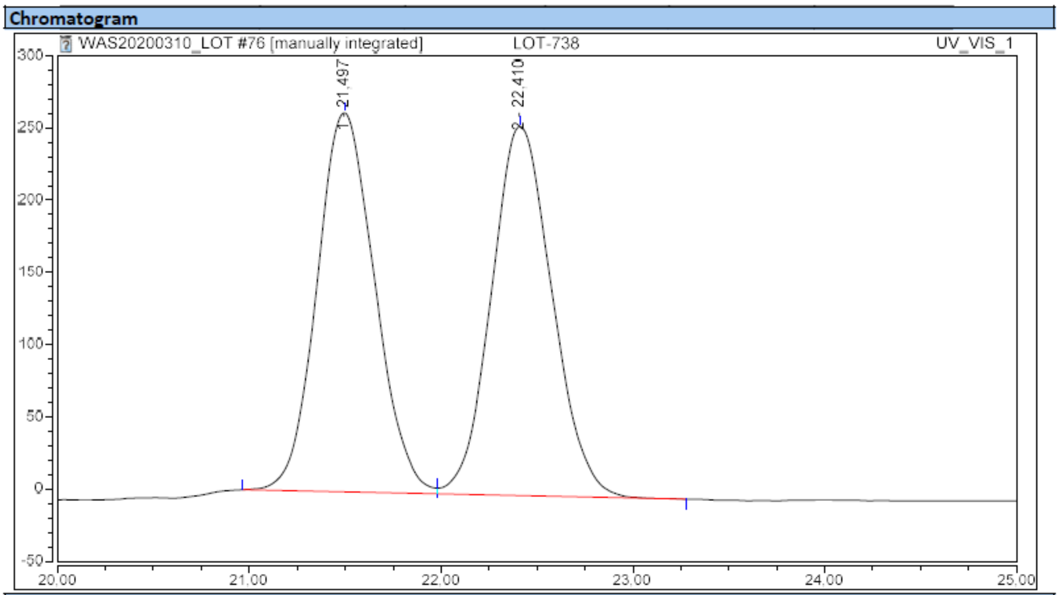

Integration Results

No.	Peak Name	Retention Time min	Area mAU*min	Height mAU	Relative Area %	Relative Height %	Amount n.a.
1		21,497	91,590	262,318	49,43	50,66	n.a.
2		22,410	93,705	255,451	50,57	49,34	n.a.
**Total:**			**185,294**	**517,769**	**100,00**	**100,00**	

HPLC chromatogram of enantioenriched **3f** $^{\mathbf{OMe}}$

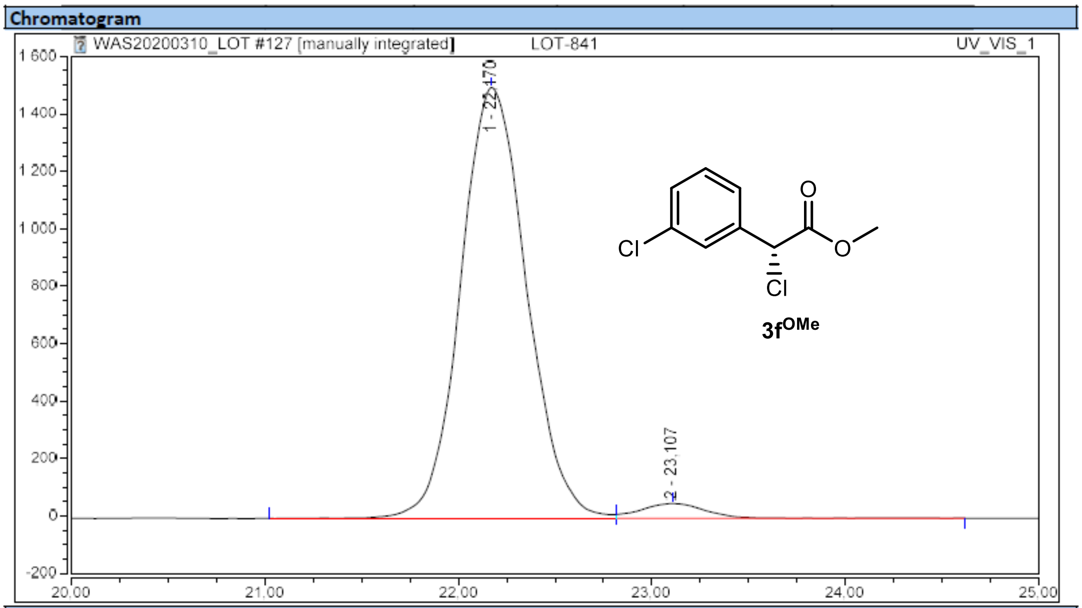

Integration Results

No.	Peak Name	Retention Time min	Area mAU*min	Height mAU	Relative Area %	Relative Height %	Amount n.a.
1		22,170	591,899	1500,261	96,72	96,68	n.a.
2		23,107	20,042	51,552	3,28	3,32	n.a.
**Total:**			**611,940**	**1551,813**	**100,00**	**100,00**	

HPLC chromatogram of (rac)-**3g** $^{\mathbf{OMe}}$

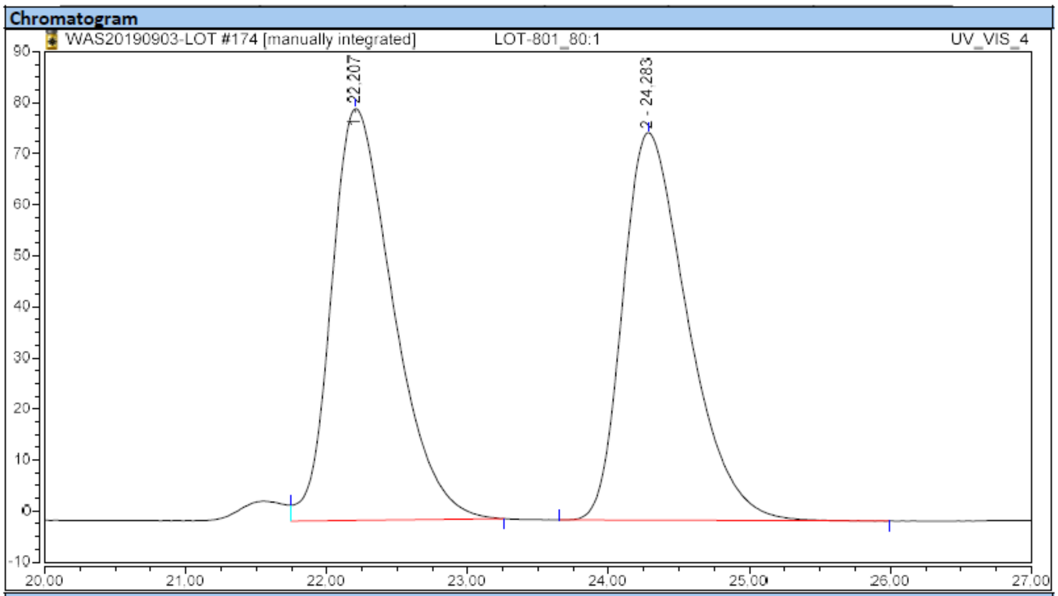

Integration Results

No.	Peak Name	Retention Time min	Area mAU*min	Height mAU	Relative Area %	Relative Height %	Amount n.a.
1		22,207	40,912	80,650	50,33	51,51	n.a.
2		24,283	40,379	75,919	49,67	48,49	n.a.
Total:			81,291	156,569	100,00	100,00	

HPLC chromatogram of enantioenriched **3g**$^{\mathbf{OMe}}$

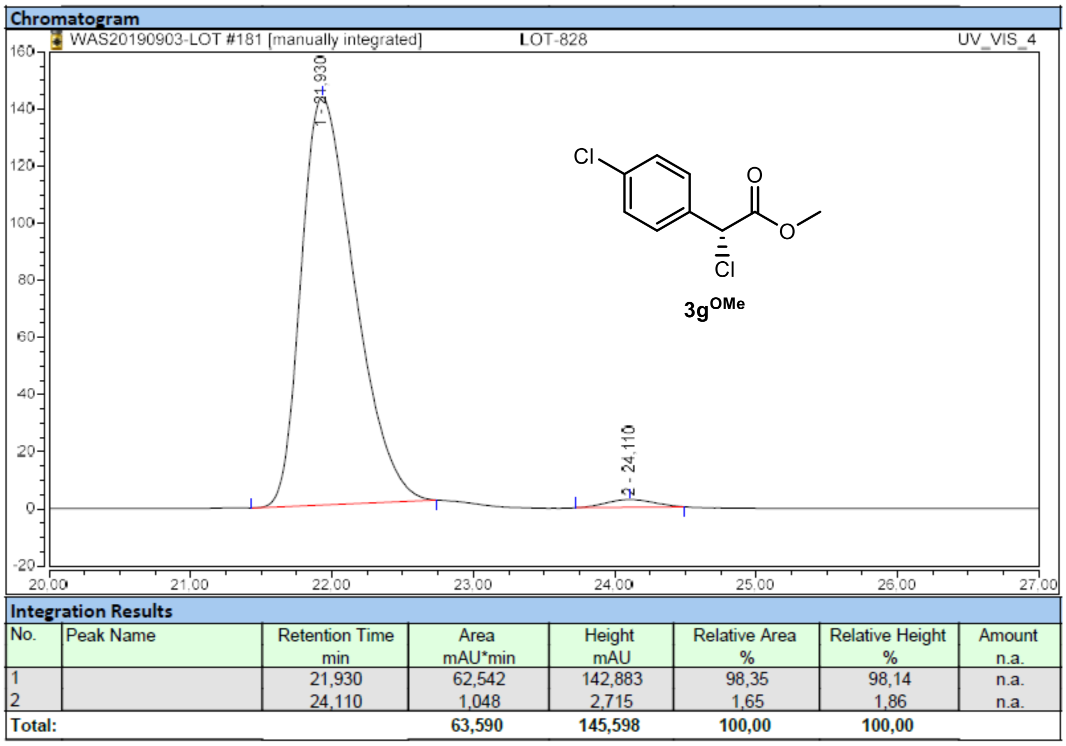

Integration Results

No.	Peak Name	Retention Time min	Area mAU*min	Height mAU	Relative Area %	Relative Height %	Amount n.a.
1		21,930	62,542	142,883	98,35	98,14	n.a.
2		24,110	1,048	2,715	1,65	1,86	n.a.
Total:			63,590	145,598	100,00	100,00	

HPLC chromatogram of (rac)-**3h^{OMe}**

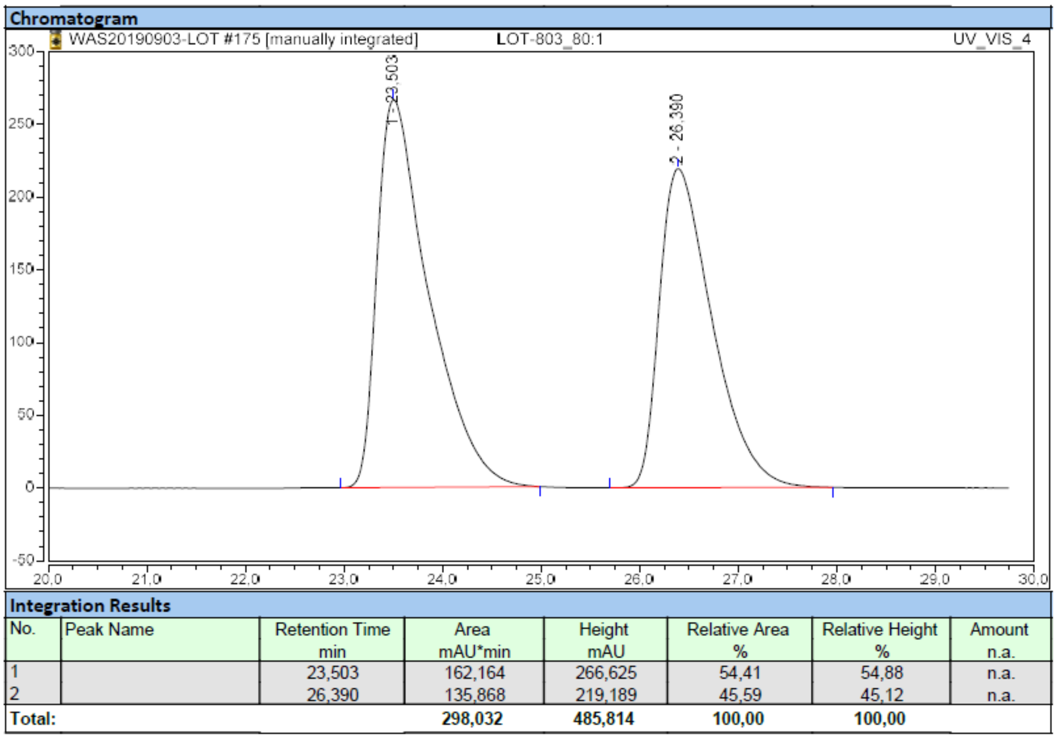

Integration Results							
No.	Peak Name	Retention Time min	Area mAU*min	Height mAU	Relative Area %	Relative Height %	Amount n.a.
1		23,503	162,164	266,625	54,41	54,88	n.a.
2		26,390	135,868	219,189	45,59	45,12	n.a.
**Total:**			**298,032**	**485,814**	**100,00**	**100,00**	

HPLC chromatogram of enantioenriched **3h^{OMe}**

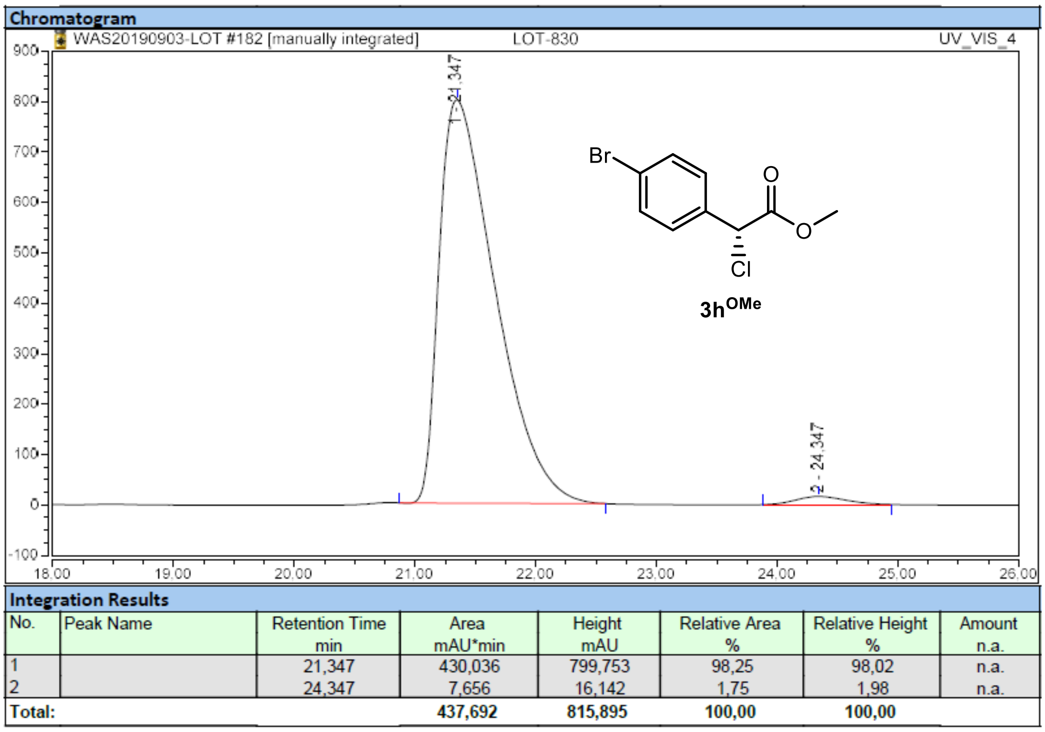

Integration Results							
No.	Peak Name	Retention Time min	Area mAU*min	Height mAU	Relative Area %	Relative Height %	Amount n.a.
1		21,347	430,036	799,753	98,25	98,02	n.a.
2		24,347	7,656	16,142	1,75	1,98	n.a.
**Total:**			**437,692**	**815,895**	**100,00**	**100,00**	

HPLC chromatogram of (rac)-**3i**$^{\mathbf{OMe}}$

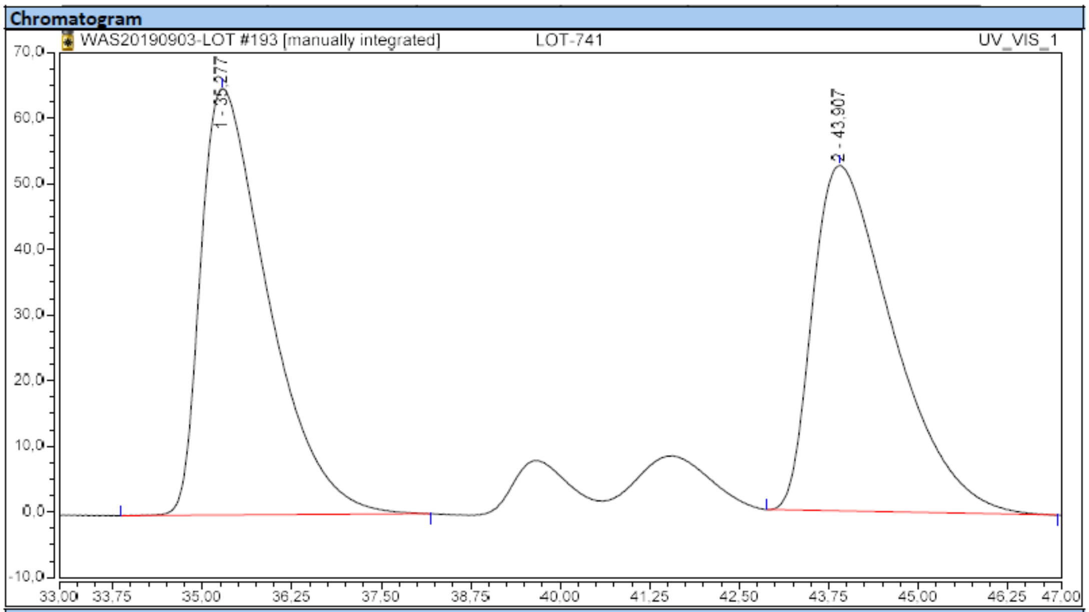

Integration Results

No.	Peak Name	Retention Time min	Area mAU*min	Height mAU	Relative Area %	Relative Height %	Amount n.a.
1		35,277	70,864	65,006	50,75	55,25	n.a.
2		43,907	68,782	52,662	49,25	44,75	n.a.
**Total:**			**139,646**	**117,668**	**100,00**	**100,00**	

HPLC chromatogram of enantioenriched **3i**$^{\mathbf{OMe}}$

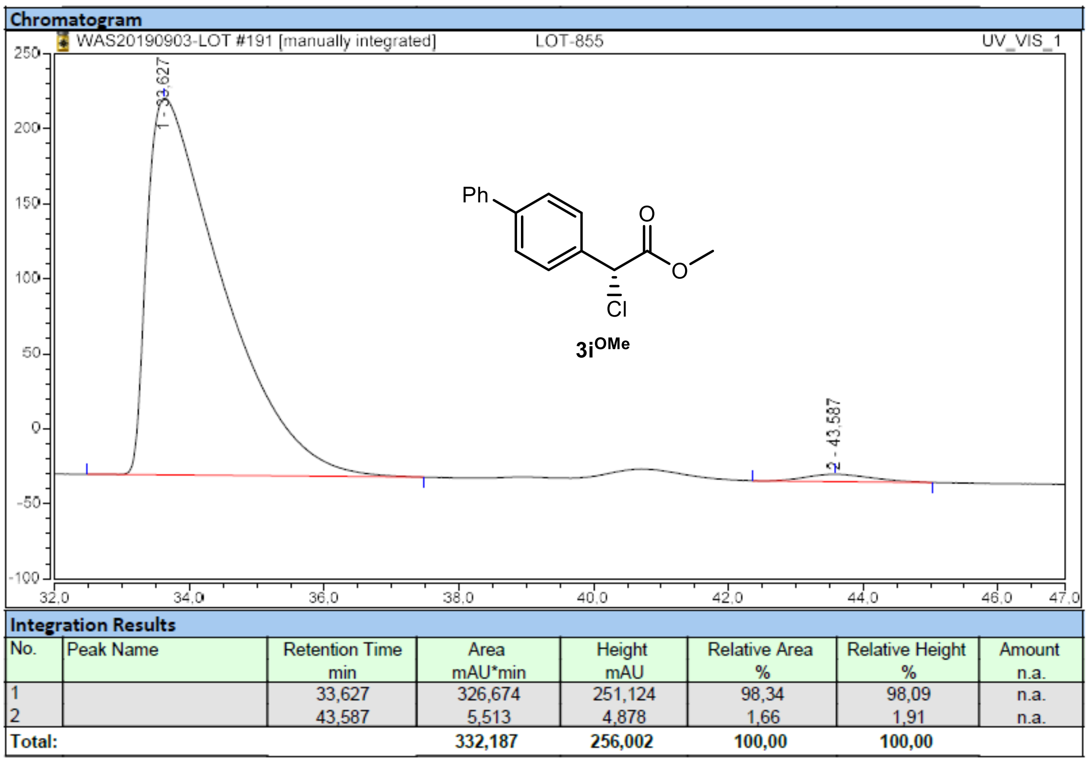

Integration Results

No.	Peak Name	Retention Time min	Area mAU*min	Height mAU	Relative Area %	Relative Height %	Amount n.a.
1		33,627	326,674	251,124	98,34	98,09	n.a.
2		43,587	5,513	4,878	1,66	1,91	n.a.
**Total:**			**332,187**	**256,002**	**100,00**	**100,00**	

HPLC chromatogram of (rac)-**3j^{OMe}**

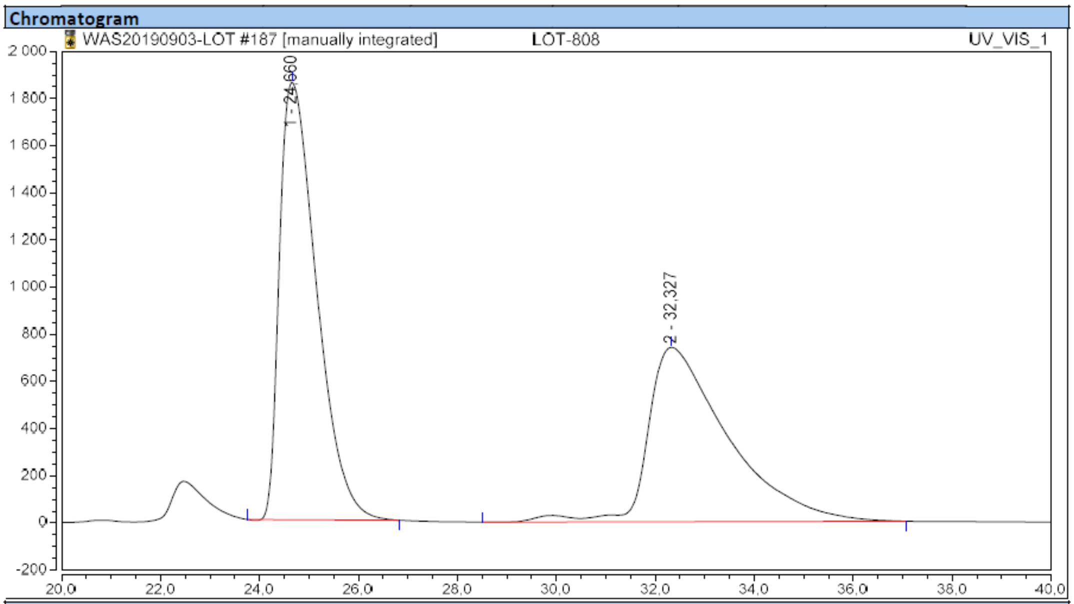

Integration Results

No.	Peak Name	Retention Time min	Area mAU*min	Height mAU	Relative Area %	Relative Height %	Amount n.a.
1		24,660	1612,548	1856,981	53,81	71,50	n.a.
2		32,327	1384,356	740,023	46,19	28,50	n.a.
**Total:**			**2996,904**	**2597,003**	**100,00**	**100,00**	

HPLC chromatogram of enantioenriched **3j^{OMe}**

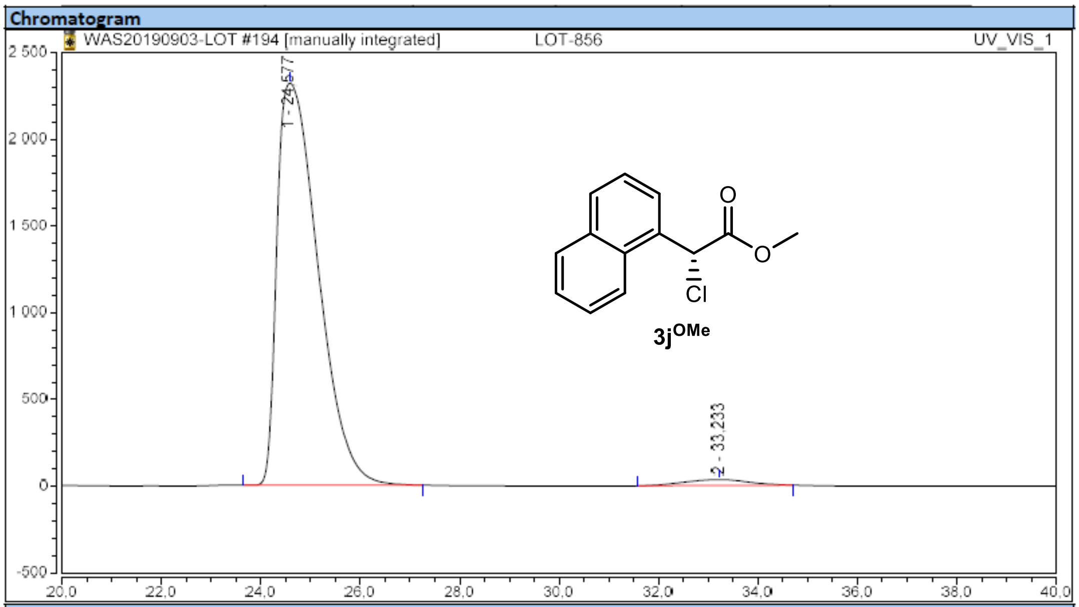

Integration Results

No.	Peak Name	Retention Time min	Area mAU*min	Height mAU	Relative Area %	Relative Height %	Amount n.a.
1		24,577	2215,429	2317,613	97,76	98,53	n.a.
2		33,233	50,733	34,577	2,24	1,47	n.a.
**Total:**			**2266,162**	**2352,190**	**100,00**	**100,00**	

HPLC chromatogram of (rac)-**3k$^{\mathbf{OMe}}$**

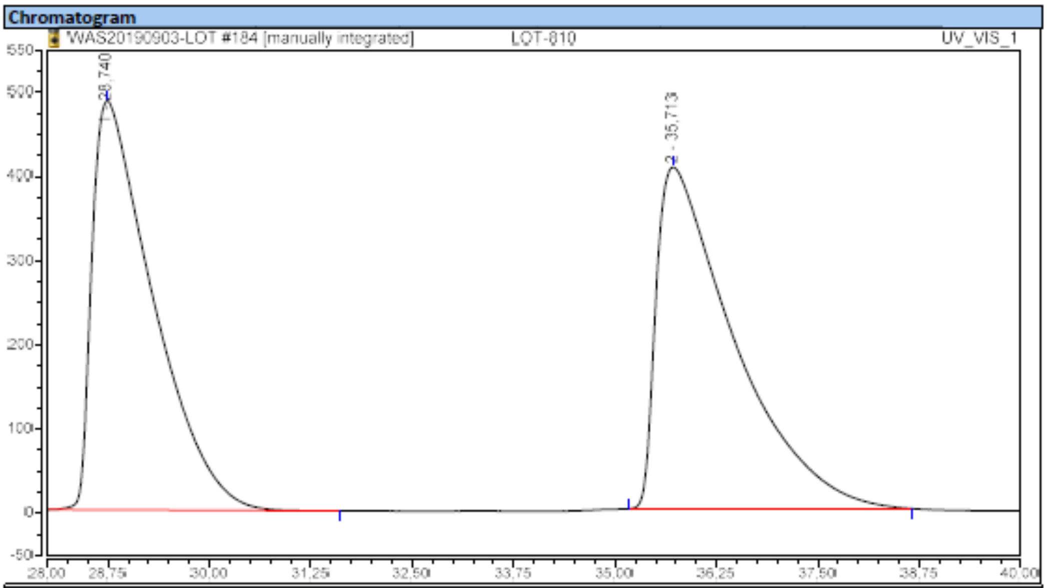

Integration Results

No.	Peak Name	Retention Time min	Area mAU*min	Height mAU	Relative Area %	Relative Height %	Amount n.a.
1		28,740	422,589	485,561	48,38	54,47	n.a.
2		35,713	450,815	405,853	51,62	45,53	n.a.
**Total:**			**873,404**	**891,414**	**100,00**	**100,00**	

HPLC chromatogram of enantioenriched **3j$^{\mathbf{OMe}}$**

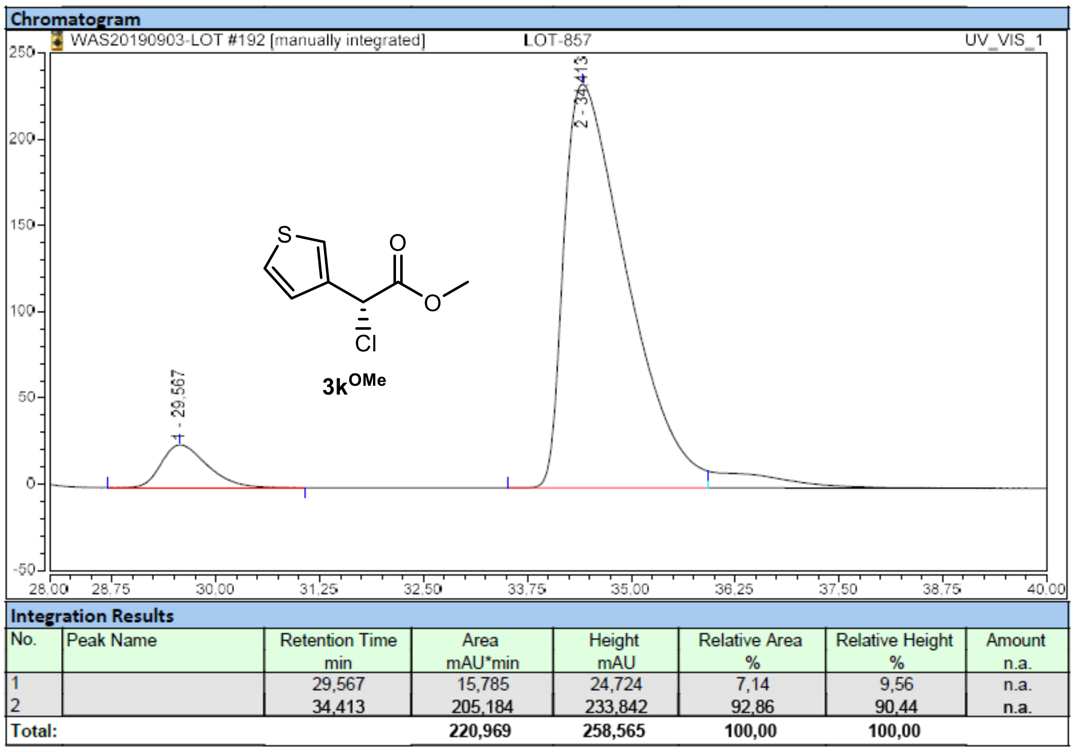

Integration Results

No.	Peak Name	Retention Time min	Area mAU*min	Height mAU	Relative Area %	Relative Height %	Amount n.a.
1		29,567	15,785	24,724	7,14	9,56	n.a.
2		34,413	205,184	233,842	92,86	90,44	n.a.
**Total:**			**220,969**	**258,565**	**100,00**	**100,00**	

HPLC chromatogram of (rac)-**3m**$^{\mathbf{OMe}}$

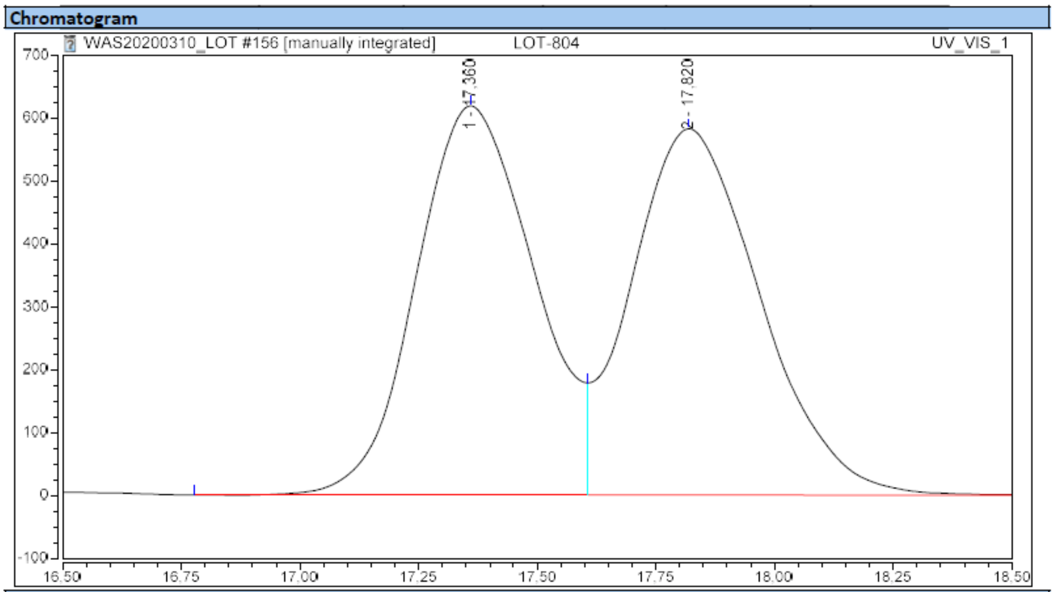

Integration Results

No.	Peak Name	Retention Time min	Area mAU*min	Height mAU	Relative Area %	Relative Height %	Amount n.a.
1		17,360	177,807	618,566	49,23	51,51	n.a.
2		17,820	183,376	582,287	50,77	48,49	n.a.
**Total:**			**361,183**	**1200,853**	**100,00**	**100,00**	

HPLC chromatogram of enantioenriched **3m**$^{\mathbf{OMe}}$

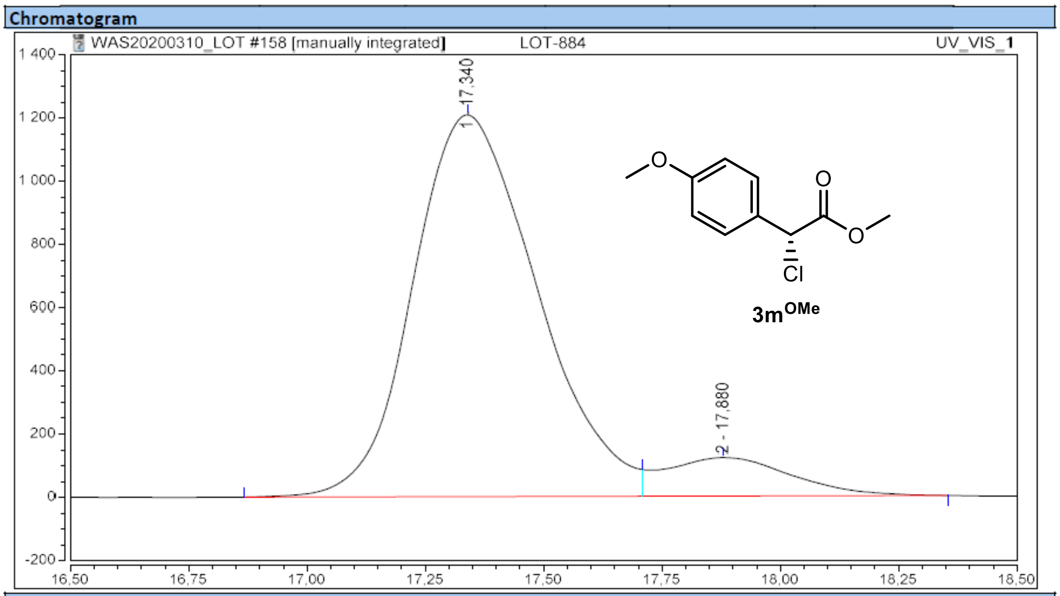

Integration Results

No.	Peak Name	Retention Time min	Area mAU*min	Height mAU	Relative Area %	Relative Height %	Amount n.a.
1		17,340	365,731	1207,371	90,73	90,87	n.a.
2		17,880	37,376	121,278	9,27	9,13	n.a.
**Total:**			**403,106**	**1328,649**	**100,00**	**100,00**	

HPLC chromatogram of (rac)-**3a**$^{\mathbf{OEt}}$

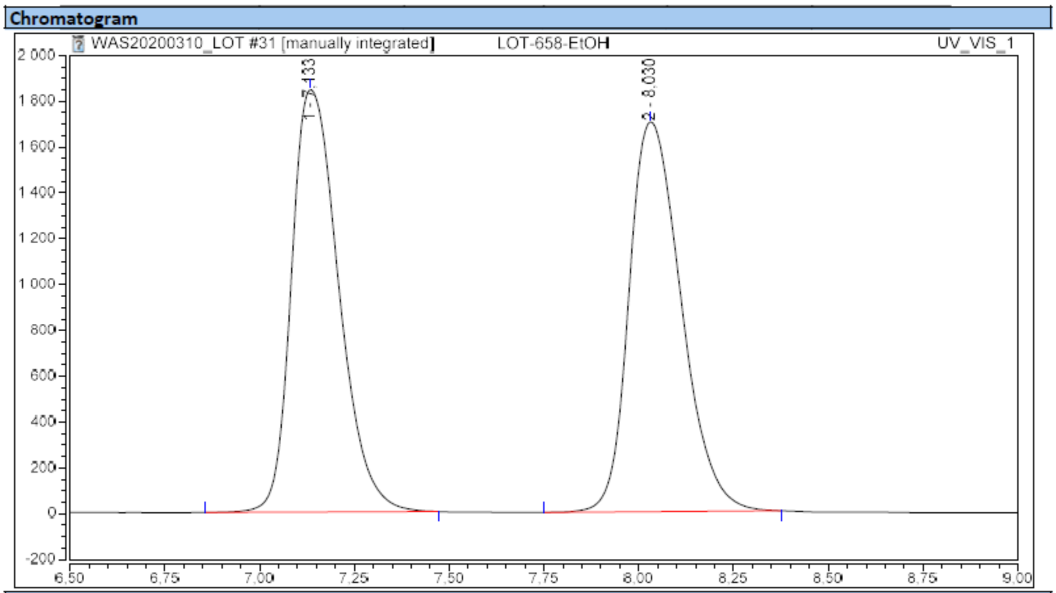

Integration Results							
No.	Peak Name	Retention Time min	Area mAU*min	Height mAU	Relative Area %	Relative Height %	Amount n.a.
1		7,133	266,345	1843,189	49,57	52,00	n.a.
2		8,030	271,013	1701,230	50,43	48,00	n.a.
Total:			537,358	3544,419	100,00	100,00	

HPLC chromatogram of enantioenriched **3a**$^{\mathbf{OMe}}$

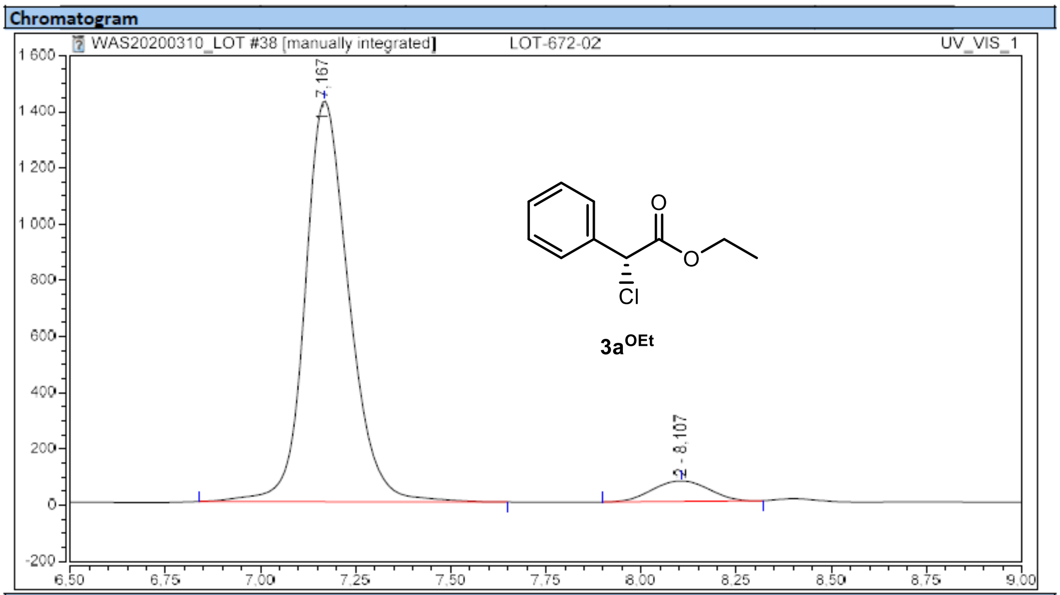

Integration Results							
No.	Peak Name	Retention Time min	Area mAU*min	Height mAU	Relative Area %	Relative Height %	Amount n.a.
1		7,167	190,859	1426,360	93,82	95,13	n.a.
2		8,107	12,568	72,987	6,18	4,87	n.a.
Total:			203,428	1499,347	100,00	100,00	

HPLC chromatogram of (rac)-**3a**$^{\mathbf{O}i\mathbf{Pr}}$

Chromatogram

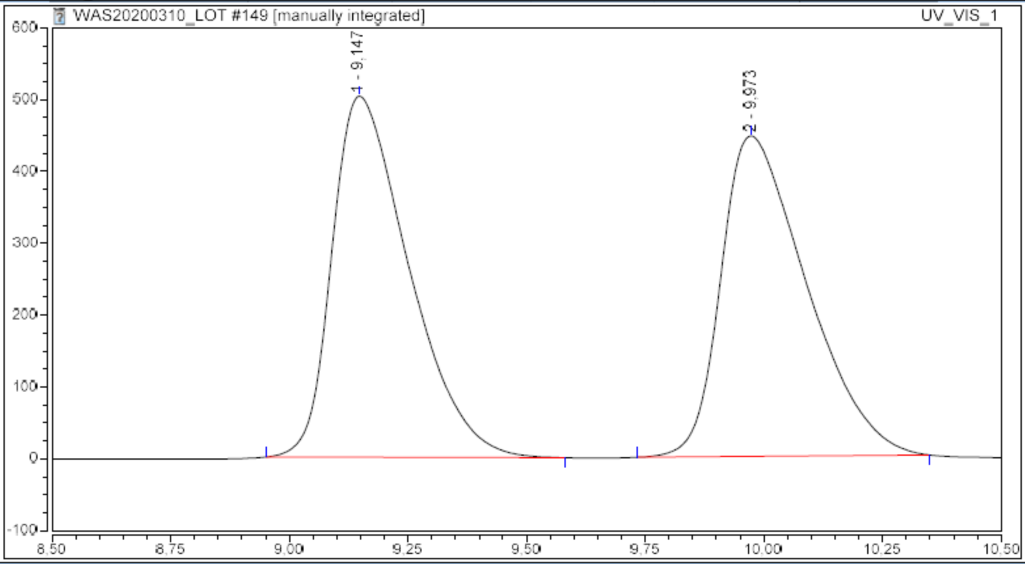

Integration Results

No.	Peak Name	Retention Time min	Area mAU*min	Height mAU	Relative Area %	Relative Height %	Amount n.a.
1		9,147	94,294	502,424	50,25	52,98	n.a.
2		9,973	93,339	445,924	49,75	47,02	n.a.
**Total:**			**187,633**	**948,348**	**100,00**	**100,00**	

HPLC chromatogram of enantioenriched **3a**$^{\mathbf{O}i\mathbf{Pr}}$

Chromatogram

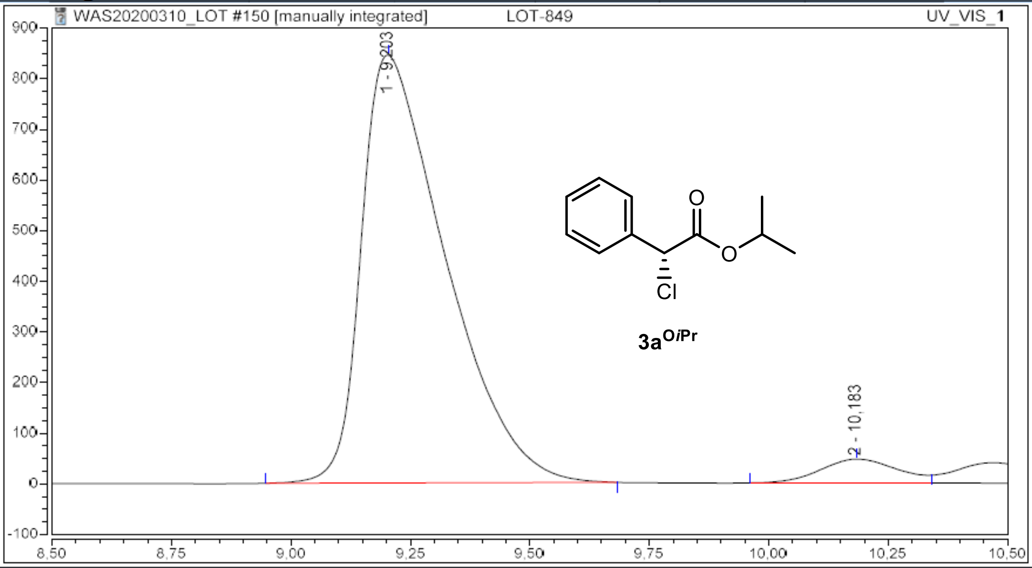

Integration Results

No.	Peak Name	Retention Time min	Area mAU*min	Height mAU	Relative Area %	Relative Height %	Amount n.a.
1		9,203	172,376	844,592	95,12	94,71	n.a.
2		10,183	8,853	47,185	4,88	5,29	n.a.
**Total:**			**181,229**	**891,776**	**100,00**	**100,00**	

HPLC chromatogram of (rac)-**3a**$^{\mathbf{NHBn}}$

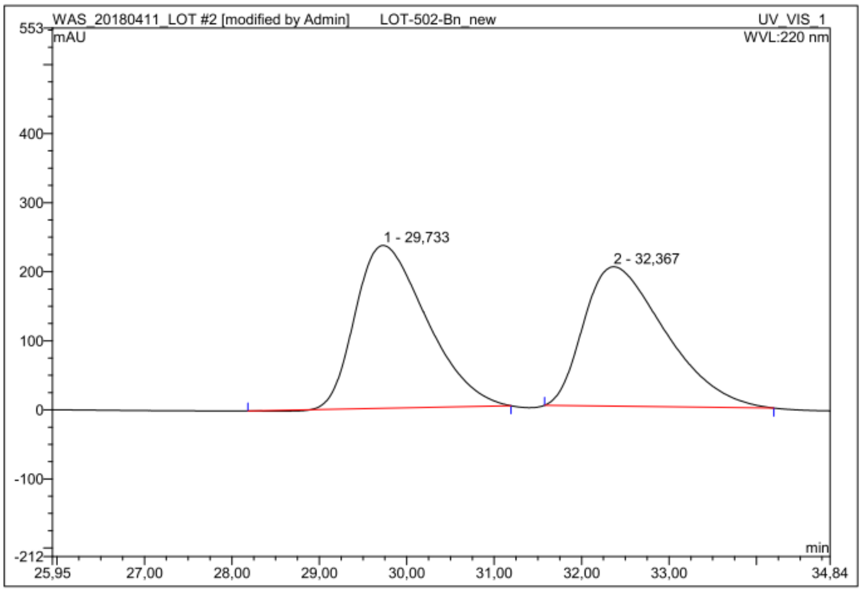

No.	Ret.Time min	Peak Name	Height mAU	Area mAU*min	Rel.Area %	Amount	Type
1	29,73	n.a.	235,860	222,676	50,45	n.a.	BMB*
2	32,37	n.a.	201,841	218,717	49,55	n.a.	BMB*
Total:			437,702	441,393	100,00	0,000	

HPLC chromatogram of enantioenriched **3a**$^{\mathbf{NHBn}}$

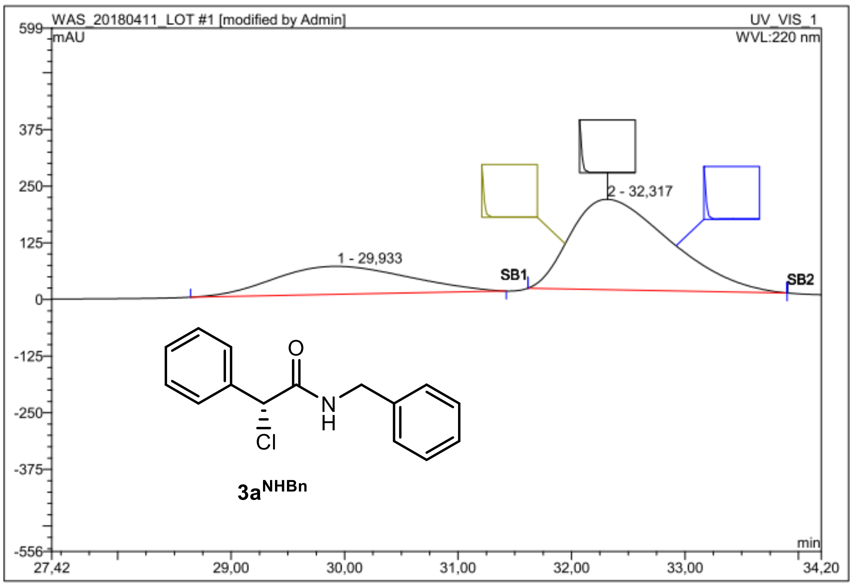

No.	Ret.Time min	Peak Name	Height mAU	Area mAU*min	Rel.Area %	Amount	Type
1	29,93	n.a.	61,866	84,466	29,33	n.a.	BMB*
2	32,32	n.a.	199,280	203,516	70,67	n.a.	BMB*
Total:			261,146	287,983	100,00	0,000	

HPLC chromatogram of (rac)-**3a**$^{\mathbf{Morpholine}}$

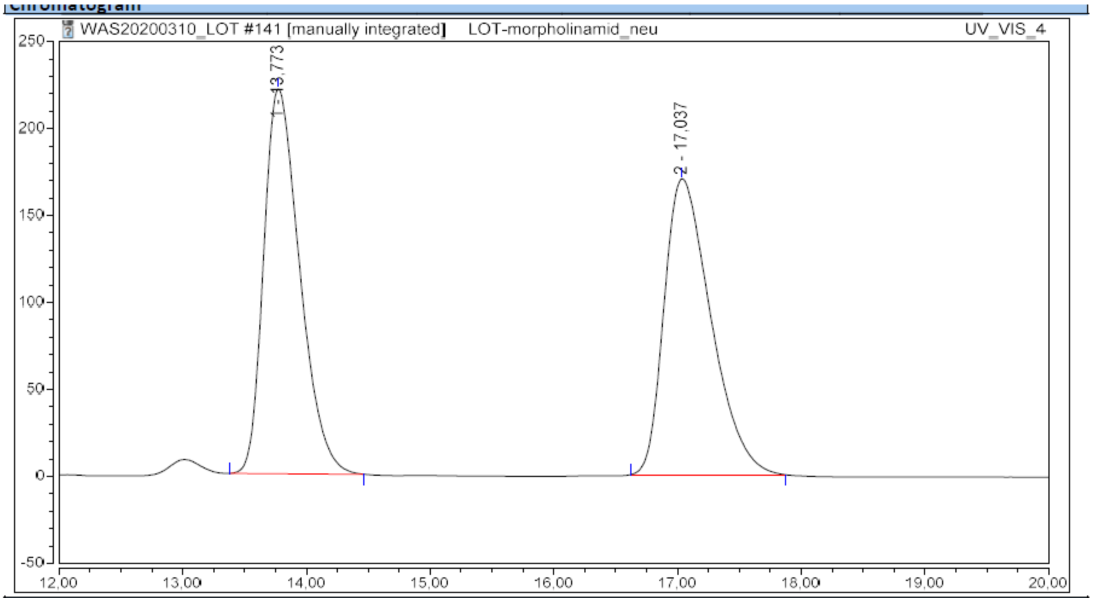

Integration Results							
No.	Peak Name	Retention Time min	Area mAU*min	Height mAU	Relative Area %	Relative Height %	Amount n.a.
1		13,773	74,703	221,220	50,11	56,53	n.a.
2		17,037	74,389	170,096	49,89	43,47	n.a.
**Total:**			**149,092**	**391,316**	**100,00**	**100,00**	

HPLC chromatogram of enantioenriched **3a**$^{\mathbf{Morpholine}}$

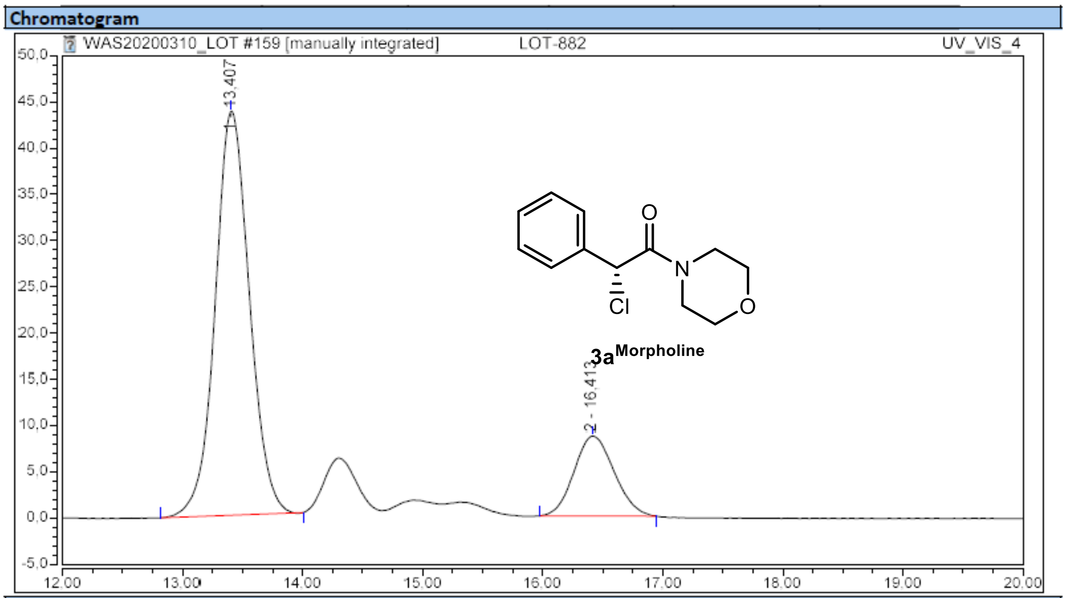

Integration Results							
No.	Peak Name	Retention Time min	Area mAU*min	Height mAU	Relative Area %	Relative Height %	Amount n.a.
1		13,407	14,431	43,742	81,18	83,50	n.a.
2		16,413	3,346	8,644	18,82	16,50	n.a.
**Total:**			**17,777**	**52,386**	**100,00**	**100,00**	
